# Template‐Controlled Synthesis of Polyimidazolium Salts by Multiple [2+2] Cycloaddition Reactions

**DOI:** 10.1002/chem.202001515

**Published:** 2020-08-06

**Authors:** Christian B. Dobbe, Ana Gutiérrez‐Blanco, Tristan T. Y. Tan, Alexander Hepp, Macarena Poyatos, Eduardo Peris, F. Ekkehardt Hahn

**Affiliations:** ^1^ Institut für Anorganische und Analytische Chemie Westfälische Wilhelms-Universität Münster Corrensstraße 30 48149 Münster Germany; ^2^ Institute of Advanced Materials (INAM) Universitat Jaume I Avda. Vicente Sos Baynat s/n Castellon 12071 Spain

**Keywords:** anion recognition, N-heterocyclic carbene, polyimidazolium salts, postsynthetic photochemical modification, silver complexes

## Abstract

The tetrakisimidazolium salt H_4_‐**2**(Br)_4_, featuring a central benzene linker and 1,2,4,5‐(*n*Bu‐imidazolium‐Ph‐CH=CH‐) substituents reacts with Ag_2_O in the presence of AgBF_4_ to yield the tetranuclear, oktakis‐NHC assembly [**3**](BF_4_)_4_. Cation [**3**]^4+^ features four pairs of olefins from the two tetrakis‐NHC ligands perfectly arranged for a subsequent [2+2] cycloaddition. Irradiation of [**3**](BF_4_)_4_ with a high pressure Hg lamp connects the two tetra‐NHC ligands through four cyclobutane linkers to give compound [**4**](BF_4_)_4_. Removal of the template metals yields the novel oktakisimidazolium salt H_8_‐**5**(BF_4_)_8_. The tetrakisimidazolium salt H_4_‐**2**(BF_4_)_4_ and the oktakisimidazolium salt H_8_‐**5**(BF_4_)_8_ have been used as multivalent anion receptors and their anion binding properties towards six different anions have been compared.

## Graphical abstract

**Polyimidazolium cations**: The octakisimidazolium salt H_8_‐**5**(BF_4_)_8_ has been obtained from two equivalents of tetrakisimidazolium salt H_4_‐**2**(BF_4_)_4_ in a template synthesis via the tetranuclear octakis‐NHC complex [Ag_4_(**2**)_2_] followed by postsynthetic irradiation and formation of four cyclobutane linkers between the two tetra‐NHC ligands and demetallation. The binding of selected anions by H_8_‐**5**(BF_4_)_4_ has been studied.

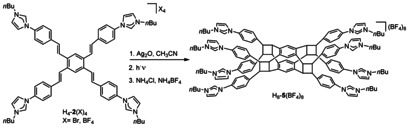

## Introduction

Coordination‐driven self‐assembly is currently one of the most effective strategies for the rational construction of discrete supramolecular coordination complexes (SSCs).^1^ This synthetic method benefits from the rich chemistry provided by transition metals and from the extensive library of organic ligands, which properly combined can produce two‐ and three‐dimensional metallosupramolecular structures for advanced applications. In most supramolecular compounds, the donor groups are restricted to those brought in by the original constituent ligands. However, there are metallosupramolecular systems that are amenable to covalent post‐assembly modification (PAM) reactions, facilitating the modification of the ligands that determine the architecture of the metallosupramolecular assembly after it has formed.^2^ PAM strategies applied to metallosupramolecular assemblies offer new pathways for the generation of supramolecular architectures with tailored functionalities. However, this strategy is underexplored compared to the widespread use of post‐synthetic modification methods that are currently known for the tailoring of metal–organic frameworks (MOFs).2b, 3 The relative lack of attention to PAM methods for discrete metallosupramolecular structures is arguably due to the more fragile and dynamic nature of self‐assembled coordination complexes,2a which hampers access to effective covalent bond formation within the assemblies. For a PAM reaction to be useful it must preserve the metallosupramolecular structure and should not interfere with the metal‐ligand bonds. Recently, ligands featuring various *N*‐heterocyclic carbene (NHC) donors have appeared as promising ligands for the design of metallosupramolecular assemblies that can be utilized for PAM processes. Due to the stability of the M−C_NHC_ bond, poly‐NHC‐derived metallosupramolecular architectures can be as stable or even more stable than supramolecular architectures derived from Werner‐type ligands.2b, 2c, 4 PAM methods applied to selected M‐poly‐NHC assemblies include oxidative C−C coupling reactions.2c In addition, photochemically induced [2+2] cycloaddition reactions using silver(I) or gold(I) complexes bearing terminal *N*‐olefin‐substituted poly‐NHCs have recently been developed as an effective PAM strategy for the connection of poly‐NHC ligands.^5^ This strategy enabled the synthesis of tetrakisimidazolium macrocycles5d, 5e from terminal olefin substituted bisimidazolium salts and of hexakis‐,5c nonakis‐ and dodekakisimidazolium5a cages from *C*_3_‐symmetric polyimidazolium precursors, respectively. The preparation of these cage‐type polyimidazolium salts is of particular interest, given that imidazolium salts are considered privileged anion receptors due to their strong affinity to anions through (C−H)^+^⋅⋅⋅X^−^ interactions, which combine hydrogen bonding with favourable electrostatic interactions.^6^

Multivalent binding interactions between a host/receptor and a guest requires that the receptor possesses more than one binding site connected through suitable spacers.^7^ Multivalency is used by nature to achieve strong, yet reversible interactions and normally enhances binding at the molecular scale. As a result, the design of multivalent scaffold architectures has enormous potential for the development of efficient receptors for various substrates. One of the advantages of synthetic multivalent receptors is that they possess a set number of defined binding sites so that a series of host‐guest complex interactions may be examined in detail.7d In this contribution we describe the preparation of a novel octakisimidazolium salt obtained in a template‐controlled synthesis starting from a tetrakisimidazolium salt. This offered the unique opportunity to compare the properties of the tetrakis‐ and octakisimidazolium salts as multivalent anion receptors.

## Results and Discussion

**Scheme 1:**
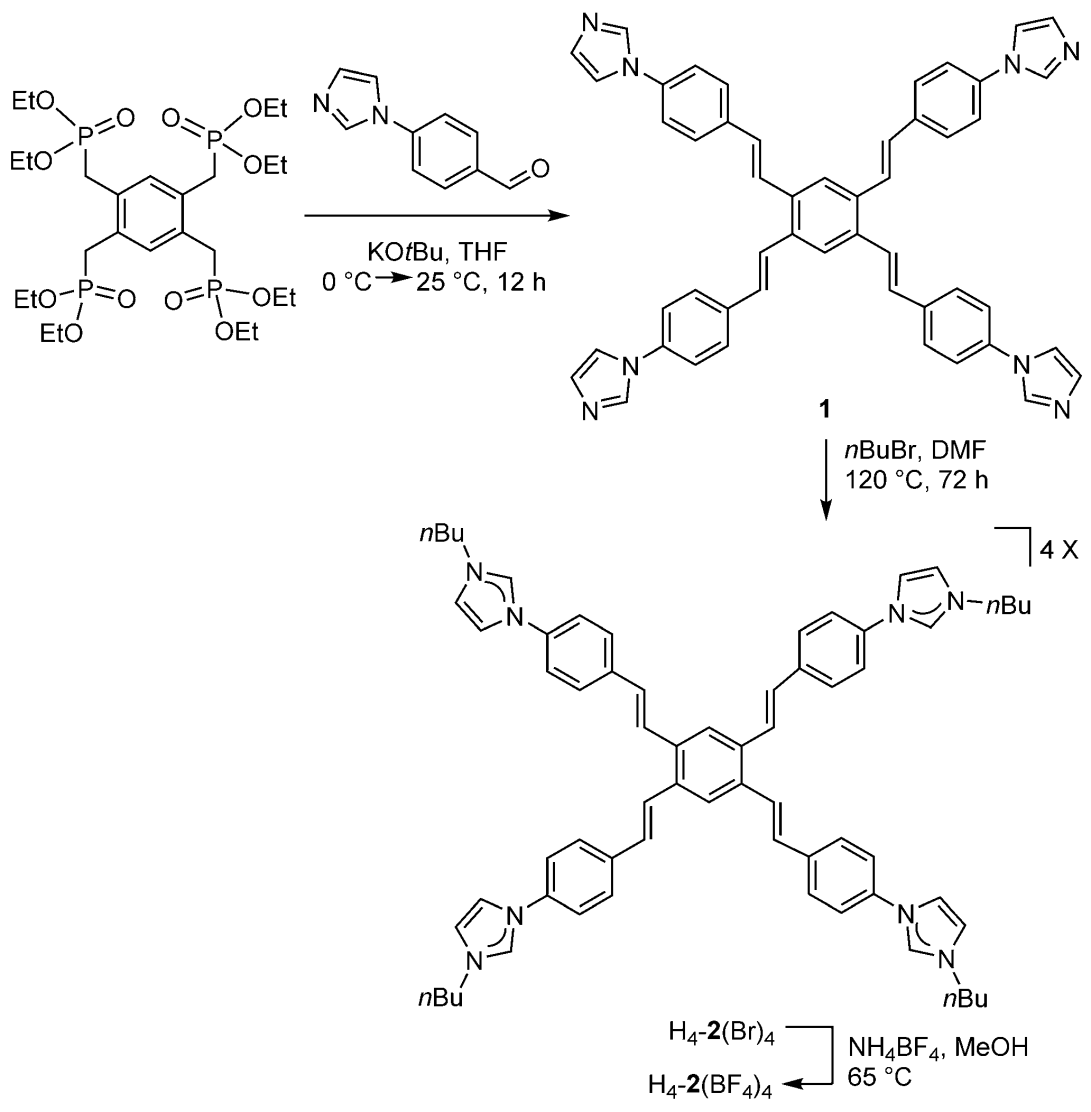
Preparation of the tetrakisimidazolium salts H_4_‐**2**(X)_4_ (X=Br, BF_4_).

For the preparation of tetrakisimidazolium salt H_4_‐**2**(BF_4_)_4_, 1,2,4,5‐tetrakis(diethoxyphosphinylmethyl)benzene^8^ and 4‐imidazol benzaldehyde^9^ were initially reacted in a Horner–Wadsworth–Emmons reaction to give compound **1** featuring four internal olefin groups. In a subsequent reaction, compound **1** was alkylated at the imidazole nitrogen atoms using 1‐bromobutane to give H_4_‐**2**(Br)_4_. Anion exchange yielded finally the tetrakisimidazolium salt H_4_‐**2**(BF_4_)_4_ (Scheme 1). Compounds H_4_‐**2**(X)_4_ (X=Br, BF_4_) were fully characterized by NMR spectroscopy, mass spectrometry and (for H_4_‐**2**(Br)_4_) by elemental analysis (see the Supporting Information).

**Scheme 2:**
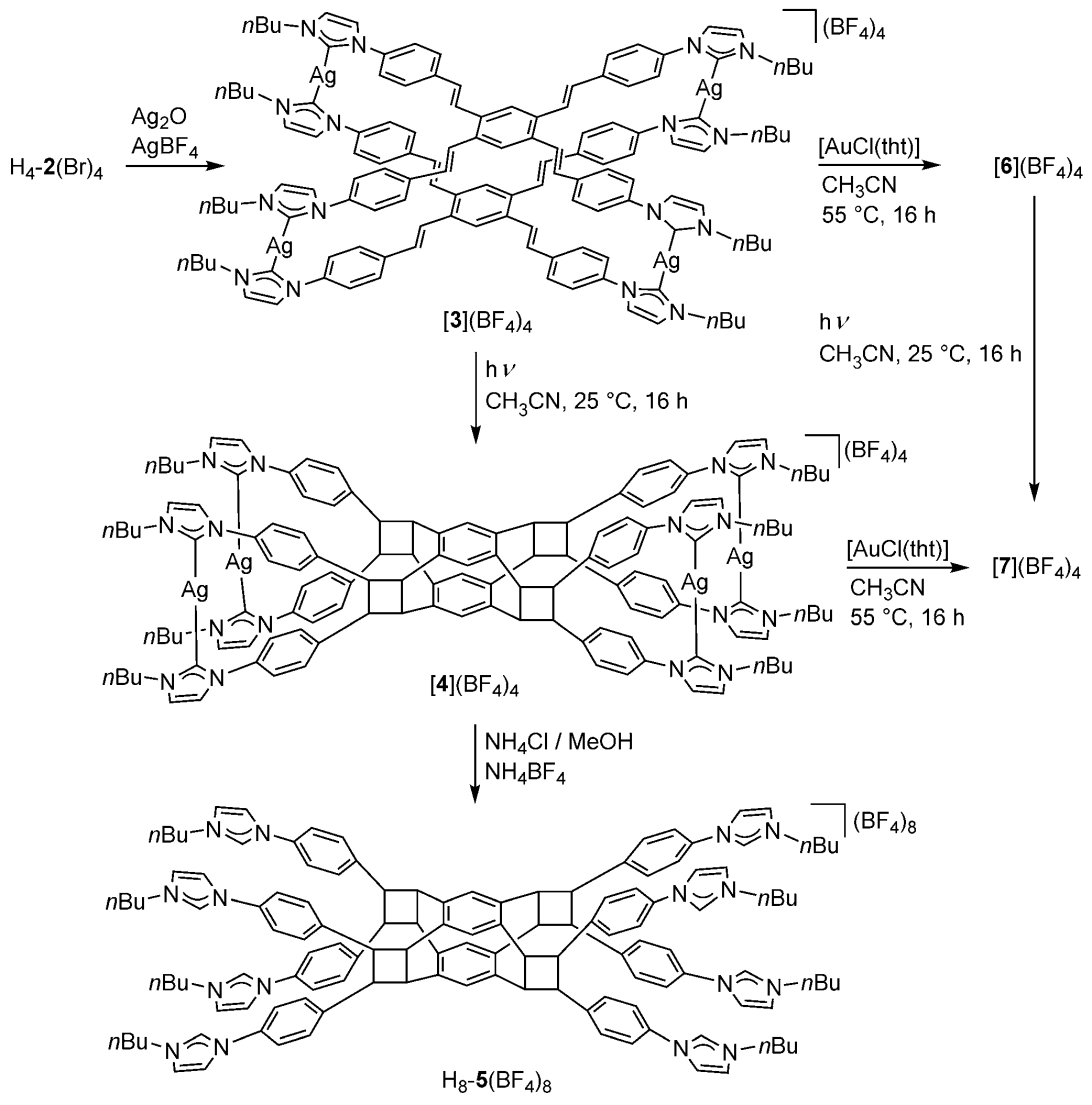
Synthesis of the tetranuclear assemblies [**3**](BF_4_)_4_, [**4**](BF_4_)_4_, [**6**](BF_4_)_4_ and of the octakisimidazolium salt H_8_‐**5**(BF_4_)_8_.

The reaction of tetrakisimidazolium salt H_4_‐**2**(Br)_4_ with a slight excess of Ag_2_O followed by addition of AgBF_4_ yielded the sandwich‐type assembly [**3**](BF_4_)_4_ in good yield of 86 % (Scheme 2). The formation of [**3**](BF_4_)_4_ was confirmed by ^1^H and ^13^C{^1^H} NMR spectroscopy. The resonances for the olefin protons were detected at *δ*=7.54 and 7.31 ppm (both doublets, ^3^ *J*_HH_=16.0 Hz) in the ^1^H NMR spectrum, only slightly shifted upfield compared to the equivalent resonances in H_4_‐**2**(BF_4_)_4_ (*δ*=7.99 and 7.59 ppm, ^3^ *J*_HH_=15.8 Hz). A characteristic doublet resonance for the C_NHC_ carbon atoms at *δ*=178.1 ppm (^1^ *J*_AgC_=149 Hz) was found in the ^13^C{^1^H} NMR spectrum. The strongest peak in the electrospray ionization mass spectrum (*m*/*z=*595.4418, calcd for [**3**]^4+^ 595.4422) also confirmed the formation of the cationic assembly [**3**]^4+^ (see the Supporting Information). Transmetallation of the tetrakis‐NHC ligand from [**3**](BF_4_)_4_ to gold(I) in [**6**](BF_4_)_4_ was achieved by reaction of [**3**](BF_4_)_4_ with [AuCl(tht)] (Scheme 2, tht=tetrahydrothiophene).

**Figure 1:**
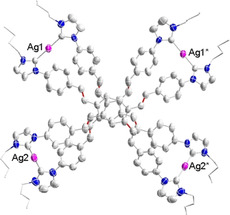
Molecular structure of cation [**3**]^4+^ in [**3**](BF_4_)_2_(BPh_4_)_2_ **⋅**4 CH_3_CN. Hydrogen atoms are omitted for clarity and the *N*‐*n*Bu groups are only depicted schematically.

Crystals of [**3**](BF_4_)_4_ suitable for an X‐ray diffraction study could not be obtained in spite of multiple attempts. However, stirring of an acetonitrile solution of [**3**](BF_4_)_4_ with four equiv of NaBPh_4_ in acetonitrile led to the isolation of the mixed anion salt [**3**](BF_4_)_2_(BPh_4_)_2_. Single crystals of [**3**](BF_4_)_2_(BPh_4_)_2_⋅4CH_3_CN were grown by slow diffusion of diethyl ether into a saturated solution of [**3**](BF_4_)_2_(BPh_4_)_2_ in acetonitrile. An X‐ray diffraction analysis with crystals [**3**](BF_4_)_2_(BPh_4_)_2_ **⋅**4 CH_3_CN confirmed the formation of the sandwich‐type complex cation [**3**]^4+^ (Figure 1). The tetracation resides on a twofold axis located in between the two central benzene rings arranged parallel to the planes of these rings. The Ag^+^ ions of [**3**]^4+^ form a rectangle with separations Ag1⋅⋅Ag2 12.400(2), Ag1⋅⋅⋅Ag1* 15.025(1) and Ag2⋅⋅⋅Ag2* 16.626(1) Å. The average Ag−C_NHC_ bond length of 2.048 Å and the average N‐C_NHC_‐N angle of 102.0° fall in the typical range for Ag‐NHC complexes.^5^ The separation between the two essentially coplanar aligned central benzene rings C_centroid_⋅⋅⋅C_centroid_ measures 3.519 Å. The olefin units of the two tetrakis‐NHC ligands are arranged in pairs with the double bonds aligned in an almost parallel fashion. The average distance of the midpoints of the C=C bonds in each pair measures 3.6 Å which is a perfect separation for a subsequent [2+2] cycloaddition reaction.^10^

**Figure 2:**
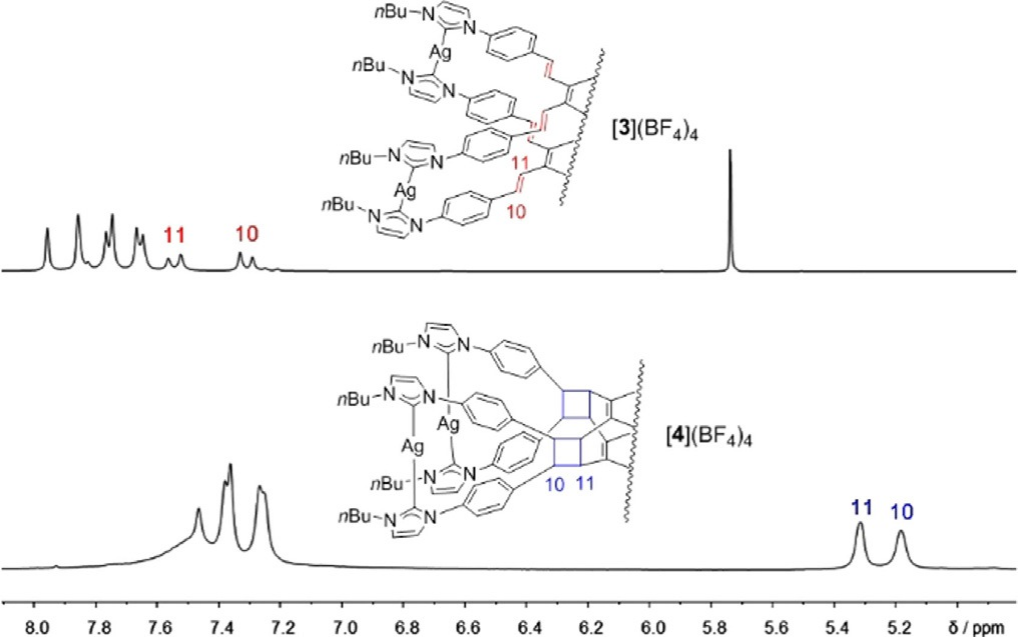
Sections of the ^1^H NMR spectra of silver(I) complexes [**3**](BF_4_)_4_ (top) and [**4**](BF_4_)_4_ (bottom).

Given the favourable orientation of the olefin groups in cation [**3**]^4+^, a post‐synthetic modification on the cation via a photochemically induced [2+2] cycloaddition reaction was investigated next. To this end, a sample of compound [**3**](BF_4_)_4_ was dissolved in acetonitrile. This solution was irradiated with a high pressure mercury lamp leading to conversion of [**3**](BF_4_)_4_ into complex [**4**](BF_4_)_4_ featuring four cyclobutane units as linkers between the two tetrakis‐NHC ligands (Scheme 2). The formation of [**4**](BF_4_)_4_ was quantitative and stereospecific as confirmed by ^1^H and ^13^C{^1^H} NMR spectroscopy (Figure 2, see also the Supporting Information). The ^1^H NMR spectrum of [**4**](BF_4_)_4_ features no resonances for the olefin protons anymore, but two new resonances at *δ*=5.32 and 5.18 ppm for the protons of the newly formed cyclobutane rings.

As was observed with salt [**3**](BF_4_)_4_, crystals for an X‐ray diffraction study of [**4**](BF_4_)_4_ could not be obtained. However, stirring of an acetonitrile solution of [**4**](BF_4_)_4_ with 4 equiv of NaBPh_4_ lead, after addition of THF and cooling, to the precipitation of crystals of composition [**4**](BPh_4_)_4_ which were suitable for an X‐ray diffraction analysis.

**Figure 3:**
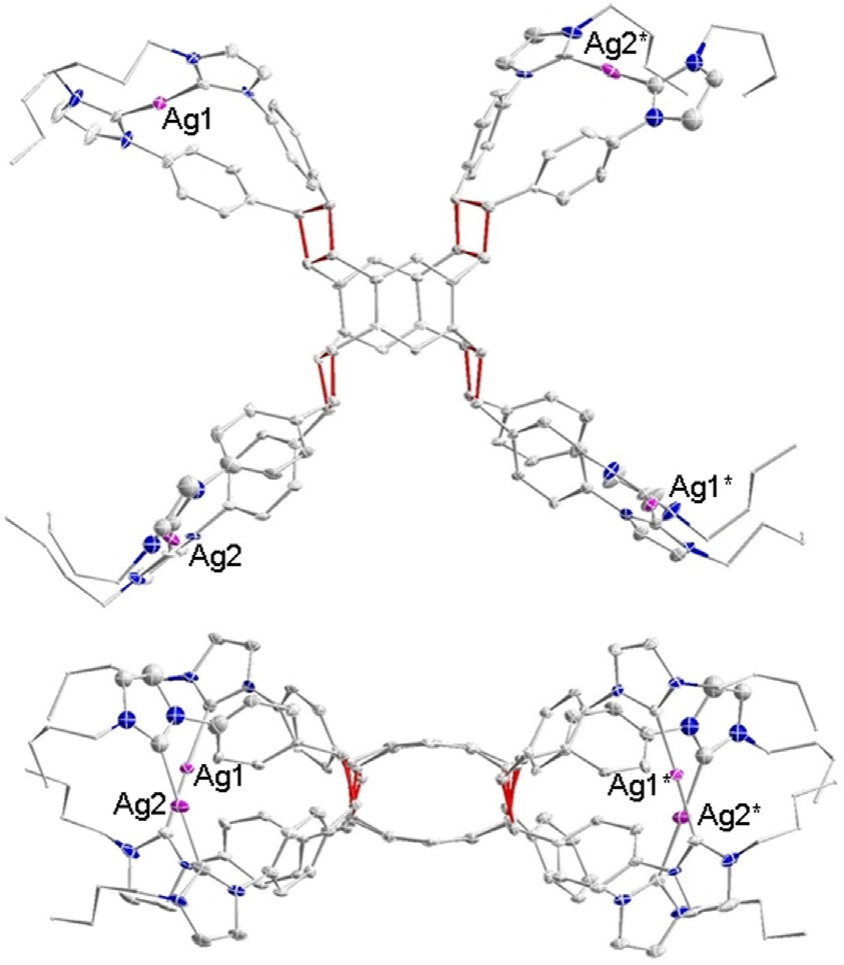
Two views of the molecular structure of cation [**4**]^4+^ in [**4**](BPh_4_)_4_. Hydrogen atoms are omitted for clarity and the *N*‐*n*Bu groups are only depicted schematically. The graphic at the bottom depicts the loss of planarity of the central benzene rings.

The diffraction analysis with crystals of [**4**](BPh_4_)_4_ (Figure 3, top) confirms the formation of the four cyclobutane linkers in tetracation [**4**]^4+^. As previously observed for [**3**]^4+^, tetracation [**4**]^4+^ also resides on a twofold axis passing through the midpoints of the two central benzene rings. The four silver atoms form a rectangle with Ag1⋅⋅⋅Ag2 and Ag1⋅⋅⋅Ag1* separations of 13.093(2) and 15.119(2) Å. The average Ag−C_NHC_ bond lengths and the average N‐C_NHC_‐N angle do not differ significantly from the equivalent parameters in [**3**]^4+^. The formation of the four cyclobutane rings in [**4**]^4+^ leads to a shortening of the distance between the midpoints of the two central benzene rings from 3.519 Å in [**3**]^4+^ to 2.837 Å in [**4**]^4+^. In addition, the central benzene rings are bent in a convex manner relative to the cyclobutane linkers (Figure 2, bottom). The average C−C separation within the cyclobutane rings measures 1.56 Å.

Addition of NH_4_Cl to a methanol solution of [**4**](BF_4_)_4_ generated a precipitate of AgCl and the octakisimidazolium salt H_8_‐**5**(Cl)_8_. Anion exchange with NH_4_BF_4_ yielded H_8_‐**5**(BF_4_)_8_ in an overall yield of 76 % (Scheme 2). Salt H_8_‐**5**(BF_4_)_8_ was characterized by ^1^H and ^13^C{^1^H} NMR spectroscopy and by mass spectrometry (see the Supporting Information). The resonances at *δ*=9.70 and at *δ*=134.7 ppm in the ^1^H and ^13^C{^1^H} NMR spectra, respectively, confirmed the demetallation and formation of the octakisimidazolium salt. The resonances for the imidazolium H2 and C2 atoms fall, as expected, in the range previously recorded for the related resonances in the tetrakisimidazolium salt H_4_‐**2**(BF_4_)_4_. The resonances for the cyclobutane protons in cation H_8_‐**5** ^8+^ were detected as two singlets at *δ*=5.10 and 5.19 ppm, only slightly shifted upfield from the equivalent resonances in [**4**](BF_4_)_4_ (*δ*=5.32 and 5.18 ppm). The ESI mass spectrum shows strong peaks for various ions [H_8_‐**5**+*n*BF_4_]^(8−n)+^ with the strongest peak (100 %) recorded at *m*/*z=*355.3715 (calcd for [H_8_‐**5**+2BF_4_]^2+^ 355.3697).

The tetranuclear silver(I) complex [**3**](BF_4_)_4_ undergoes a transmetallation reaction with [AuCl(tht)] to give the tetranuclear gold(I) complex [**6**](BF_4_)_4_ in 70 % yield (Scheme 2) similar to our previous observations with related tetranuclear silver(I)‐NHC complexes.^11^ The formation of complex [**6**](BF_4_)_4_ was confirmed by ^1^H and ^13^C{^1^H} NMR spectroscopy (*δ*(C_NHC_)=180.3 ppm, see the Supporting Information). The ESI mass spectrum shows the strongest peak (100 %) at *m*/*z=*684.5022 (calcd 684.5035 for [**6**]^4+^). Gold complex [**6**](BF_4_)_4_ also reacts quantitatively upon irradiation (high pressure mercury lamp) in a [2+2] cycloaddition reaction to give compound [**7**](BF_4_)_4_ featuring four cyclobutane linkers between the two tetra‐NHC ligands. Alternatively, compound [**7**](BF_4_)_4_ can be generated via a transmetallation reaction from silver complex [**4**](BF_4_)_4_ and [AuCl(tht)] in 59 % yield. Gold compound [**7**](BF_4_)_4_ was fully characterized by ^1^H and ^13^C{^1^H} NMR spectroscopy (*δ*(C_NHC_)=184.1 ppm) and by ESI mass spectrometry (see the Supporting Information). While gold complex [**7**](BF_4_)_4_ is easily accessible, it is a less suitable precursor for the liberation of the tetrakisimidazolium salt H_8_‐**5**(BF_4_)_8_ since the Au−C_NHC_ bonds are less labile compared to the Ag−C_NHC_ bonds. This causes problems in the liberation of the H_8_‐**5**(BF_4_)_8_ from [**7**](BF_4_)_4_.

The preparation of the polyimidazolium salts H_4_‐**2**(BF_4_)_4_ and H_8_‐**5**(BF_4_)_8_ offers a good opportunity to compare their capabilities as multivalent anion receptors. The recognition and binding properties of the anions in the two salts were studied by ^1^H NMR titration experiments monitoring selected proton signals of the receptors upon addition of the tetrabutylammonium salts of the investigated anions. All titrations studies were carried out in [D_6_]DMSO. For the study we selected six anions of different geometry and charge density, namely chloride, bromide, nitrate, benzoate, adenosine triphosphate (ATP^−^) and 2‐(4‐isobutylphenyl)propionate (IBF^−^). The last two anions were selected in an effort to demonstrate the relevance of the two polyazolium salts as receptors of anions with biological and medical interest (2‐(4‐isobutylphenyl)propionic acid is ibuprofen, H‐IBF). Generally, addition of solutions containing the anions induced significant perturbations in the ^1^H NMR spectra of the polyimidazolium hosts.

**Figure 4:**
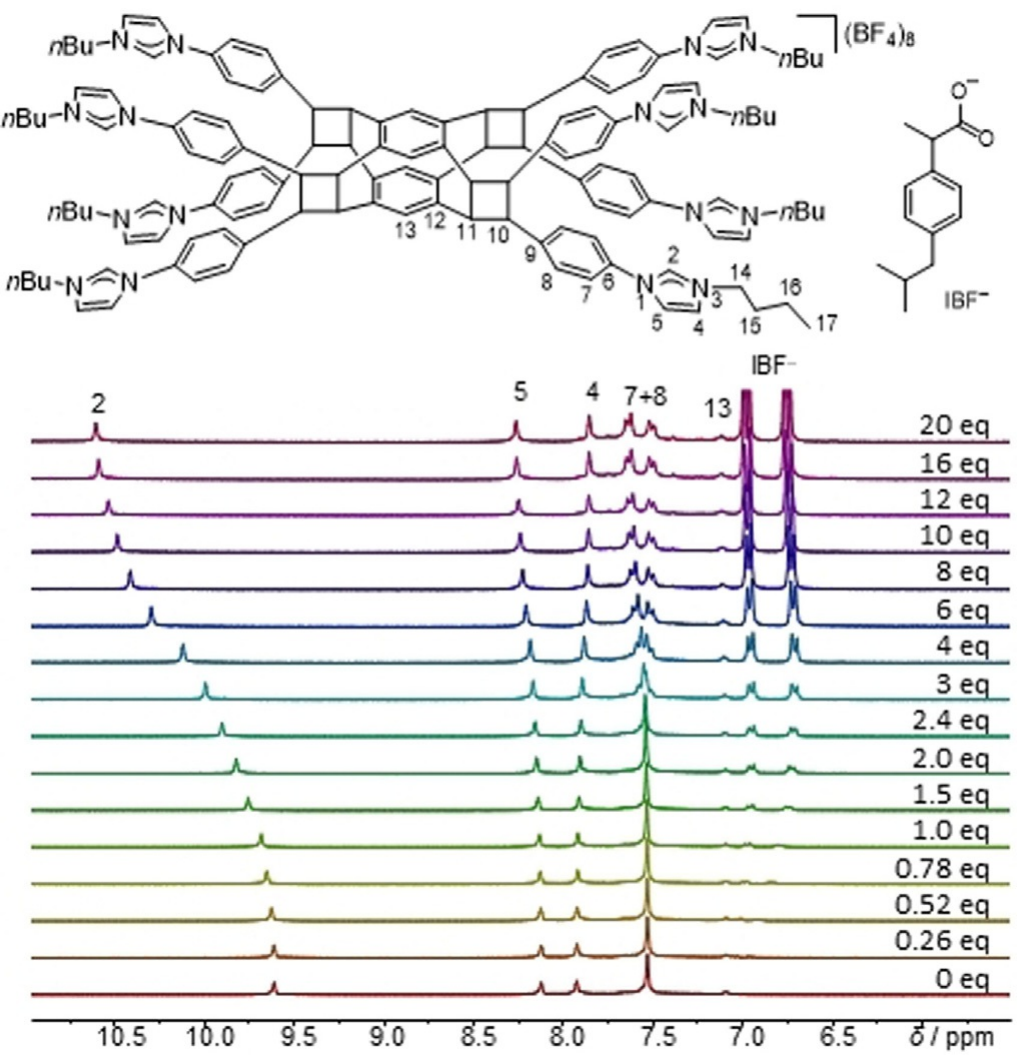
Regions of the ^1^H NMR spectra (300 MHz) recorded during the titration of H_8_‐**5**(BF_4_)_8_ (2 mm) with [NBu_4_^+^](IBF^−^) in [D_6_]DMSO.

As an illustrative example, Figure 4 shows the ^1^H NMR spectra of H_8_‐**5**(BF_4_)_8_ upon titration with [NBu_4_^+^](IBF^−^). The spectra illustrate how the resonance for the eight equivalent acidic imidazolium protons (H2) is shifted progressively downfield upon addition of increasing amounts of IBF^−^. Together with this, one of the resonances due to the remaining protons of the imidazolium ring (H5) is slightly downfield shifted, while the resonance for the other one (H4) moves slightly upfield (Figure 4). These observations strongly suggest that the interaction of the IBF^−^ anion with H_8_‐**5** ^8+^ mainly happens at the periphery of the host, with a maximum participation of the imidazolium rings via a hydrogen bonding interaction of the acidic imidazolium protons with the anion.

The determination of the binding constants between the anions and H_8_‐**5** ^8+^ was performed by global non‐linear regression analysis of the ^1^H NMR titration data.^12^ For the titrations using H_4_‐**2**(BF_4_)_4_ as the host, the stoichiometry of the host:guest complexes formed was best fitted to a 1:2 stoichiometry (two anions bound to the tetrakisimidazolium receptor). This assumption was based on the analysis of the binding isotherms resulting from the titrations of host H_4_‐**2**(BF_4_)_4_ with all six anions. In all cases, the 1:2 stoichiometry gave the best distribution of residuals, compared to a 1:1 stoichiometry.12b The 1:2 stoichiometry was also supported by the Job plot analysis (see the Supporting Information for details). This observation indicates that the binding can be described as the hydrogen bonding interaction of each anion with two imidazolium moieties of the receptor.

For the experiments performed with H_8_‐**5**(BF_4_)_8_, the Job plot analyses suggested a stoichiometry that could vary between 1:3 or 1:4 (the maximum χ values assumed values between 0.20–0.25). We are perfectly aware that the Job plot analysis has serious limitations, particularly when referred to models of high stoichiometry,^13^ but in this case and considering the results obtained for the titrations with H_4_‐**2**(BF_4_)_4_, the 1:4 stoichiometry in which two arms of the receptor are bound to each anion seems to be the most plausible one.

For the determination of the binding constants, a 1:2 stoichiometry model was used for both the tetrakisimidazolium and the octakisimidazolium salts. The 1:4 (or 1:3) models were not used for evaluating the titrations with H_8_‐**5**(BF_4_)_8_ due to the limitations of the regression analysis used to process the data obtained from ^1^H NMR titrations. For the determination of the constants we also considered two different variants of the 1:2 binding model, depending on whether the two stepwise binding constants are linked (thus assuming a non‐cooperative binding mode, in which *K*_11_=4*K*_12_), or not (cooperative, *K*_11_≠4*K*_12_).7a This is an important point to consider, because it determines how many parameters will need to be fitted during the regression fitting process,^14^ and this can be used to assess the reliability of the result.

As can be seen from the data in Table 1, the binding constants obtained for the tetrakisimidazolium salt H_4_‐**2**(BF_4_)_4_, did not differ much regardless of whether we used a cooperative or non‐cooperative binding model (entries 1–6). This allows us to assume that the binding of the anions followed a non‐cooperative binding model, and this was the model that we used for the determination of the association constants when the octakisimidazolium salt H_8_‐**5**(BF_4_)_8_ was used (entries 7–12). The data reflect that the affinities shown for chloride are higher than that shown for bromide, in agreement with the larger basicity of the former one. Both receptors show larger affinities for carboxylates and ATP^−^. The two carboxylate anions (benzoate and IBF^−^) exhibited a rather similar affinity and the binding constant shown for ATP^−^ was the largest found for both receptors, therefore showing large selectivity for this anion. The larger affinities observed for phosphate anions when polyazolium receptors are used have been observed before.^15^ It is important to point out, that for all anions tested, we found that the affinities for the octakisimidazolium receptor H_8_‐**5**(BF_4_)_8_ were between 3–8 times larger than those obtained when H_4_‐**2**(BF_4_)_4_ was used.

**Table 1:** Association constants for the formation of host‐guest complexes between salts H_4_‐**2**(BF_4_)_4_ and H_8_‐**5**(BF_4_)_8_ and some selected anions, in [D_6_]DMSO at 25 °C.

Entry	Host	Guest	*K*_11_ [M^−1^]	*K*_12_ [M^−1^]
1	H_4_‐**2**(BF_4_)_4_	ATP^‐^	1.60(2)×10^3[a]^ 1.7(1)×10^3[b]^	4.0×10^2^ 2.5(2)×10^2[b]^
2	H_4_‐**2**(BF_4_)_4_	benzoate	200(4)^[a]^ 290(8)^[b]^	49 34(2)^[b]^
3	H_4_‐**2**(BF_4_)_4_	Cl^‐^	70(1)^[a]^ 60(2)^[b]^	18 N/A^[b]^
4	H_4_‐**2**(BF_4_)_4_	Br^‐^	45(1)^[a]^ 40(1)^[b]^	10 N/A^[b]^
5	H_4_‐**2**(BF_4_)_4_	NO_3_^−^	31(1)^[a]^ 29(1)^[b]^	8 N/A^[b]^
6	H_4_‐**2**(BF_4_)_4_	IBF^‐^	133(2)^[a]^ 91(1)^[b]^	33 N/A^[b]^
7	H_8_‐**5**(BF_4_)_8_	ATP^‐^	8.4(4)×10^3^	2.1×10^3^
8	H_8_‐**5**(BF_4_)_8_	benzoate	7.4(1)×10^2^	1.8×10^2^
9	H_8_‐**5**(BF_4_)_8_	Cl^‐^	4.53(5)×10^2^	1.25×10^2^
10	H_8_‐**5**(BF_4_)_8_	Br^‐^	86(3)	21(1)
11	H_8_‐**5**(BF_4_)_8_	NO_3_^‐^	390(9)	98(2)
12	H_8_‐**5**(BF_4_)_8_	IBF^‐^	760(8)	190(2)×10^2^

[a] Association constants calculated by global nonlinear regression analysis and assuming a non‐cooperative 1:2 (H:G) binding model. All anions added as tetrabutyl ammonium salts. [b] Association constants calculated without parameter restrictions. N/A: the value resulting from the fitting was too small or did not have any physical meaning.

## Conclusions

In summary, we have demonstrated the template synthesis of the novel octakisimidazolium salt H_8_‐**5**(BF_4_)_8_ from two tetrakisimidazolium building blocks H_4_‐**2**(BF_4_)_4_. The intermediate octakis‐NHC complex [**4**](BF_4_)_4_ with four cyclobutane linkers features two non‐planar central benzene groups. The tetrakis‐ and the octakisimidazolium salts were tested as multivalent receptors of six different anions, including two with relevant biological and medical significance (ATP^−^ and IBF^−^). From our study, we concluded that the octakisimidazolium salt exhibited a larger binding affinity for all six anions tested. Given that we did not find reasons to conclude that the stepwise binding of the anions followed a cooperative model, we believe that the enhancement of the binding affinity should be ascribed to the larger electrostatic attraction produced between the anionic guests and the octacationic octakisimidazolium receptor compared to the tetracationic tetrakisimidazolium one. With our work we proved that the template‐controlled preparation of polyimidazolium salts offers a unique opportunity to generate multivalent receptors with enhanced recognition abilities.

## Experimental

Full details of synthesis and characterisation can be found in the Supporting Information.

Deposition Numbers 1972486 ([**3**](BF_4_)_2_(BPh_4_)_2_**⋅**4 MeCN), and 1972487 ([**4**](BPh_4_)_4_)  contain the supplementary crystallographic data for this paper. These data are provided free of charge by the joint Cambridge Crystallographic Data Centre and Fachinformationszentrum Karlsruhe Access Structures service www.ccdc.cam.ac.uk/structures.

## Conflict of interest

The authors declare no conflict of interest.

## Supporting information

As a service to our authors and readers, this journal provides supporting information supplied by the authors. Such materials are peer reviewed and may be re‐organized for online delivery, but are not copy‐edited or typeset. Technical support issues arising from supporting information (other than missing files) should be addressed to the authors.

Supplementary
