# Supplementary material for: Template‐Controlled Synthesis of Polyimidazolium Salts by Multiple [2+2] Cycloaddition Reactions
Source: Chemistry. 2020 Aug 6;26(50):11565–70. doi: 10.1002/chem.202001515 (PMC7540564; doi:10.1002/chem.202001515)
Supplement: Supplementary file 1 — Supplementary [file CHEM-26-11565-s001.pdf]

Chemistry–A European Journal

Supporting Information

Template-Controlled Synthesis of Polyimidazolium Salts by Multiple [2+2] Cycloaddition Reactions

Christian B. Dobbe,[a] Ana Gutiérrez-Blanco,[a, b] Tristan T. Y. Tan,[a] Alexander Hepp,[a] Macarena Poyatos,[b] Eduardo Peris,[b] and F. Ekkehardt Hahn*[a]

Table of Contents

1.	General comments	S1
2.	Synthesis and characterization of new compounds	S1
3.	NMR spectra of new compounds	S7
4.	X-ray crystallography	S25
	Table S1. Crystal data and structure refinement for [**3**]$(BF_4)_2(BPh_4)_2\cdot 4MeCN$	S26
	Table S2. Crystal data and structure refinement for [**4**]$(BPh_4)_4$	S27
5.	Titration experiments	S28
5.1.	^{1}H NMR titration experiments with H_8-**5**$(BF_4)_8$	S29
5.2.	^{1}H NMR titration experiments with H_4-**2**$(BF_4)_4$	S41
5.3.	Job-Plots	S60
6.	DOSY experiments	S72
7.	References	S75

1. General Information

All reactions were carried out under a nitrogen atmosphere using standard Schlenk techniques and high vacuum. THF was distilled by standard procedures prior to use from Na/benzophenone. Other solvents were used without further purification. Compounds 1,2,4,5-tetrakis(diethoxyphosphinyl-methyl)benzene[1] and 4-imidazole benzaldehyde[2] were prepared as described in the literature. NMR spectra were recorded at 400 MHz (^{1}H NMR) and 101 MHz (^{13}C{^{1}H}) on Bruker AVANCE I 400 and Bruker AVANCE III 400 spectrometers. Chemical shifts (δ) are expressed in ppm downfield from tetramethylsilane using the residual protonated solvent as an internal standard. NMR spectra were recorded at room temperature in MeCN-d_3 or DMSO-d_6. Mass spectra were obtained with an Orbitrap LTQ XL (Thermo Scientific, ESI) or a Finnigan MAT95 (EI) spectrometer. Elemental analyses were performed with a Vario EL III CHNS elemental analyzer. A Bruker AVANCE III 300.12 MHz spectrometer was used for NMR-titration experiments and a Varian Innova 500 MHz spectrometer was used for the DOSY experiments.

2. Synthesis and characterization of new compounds

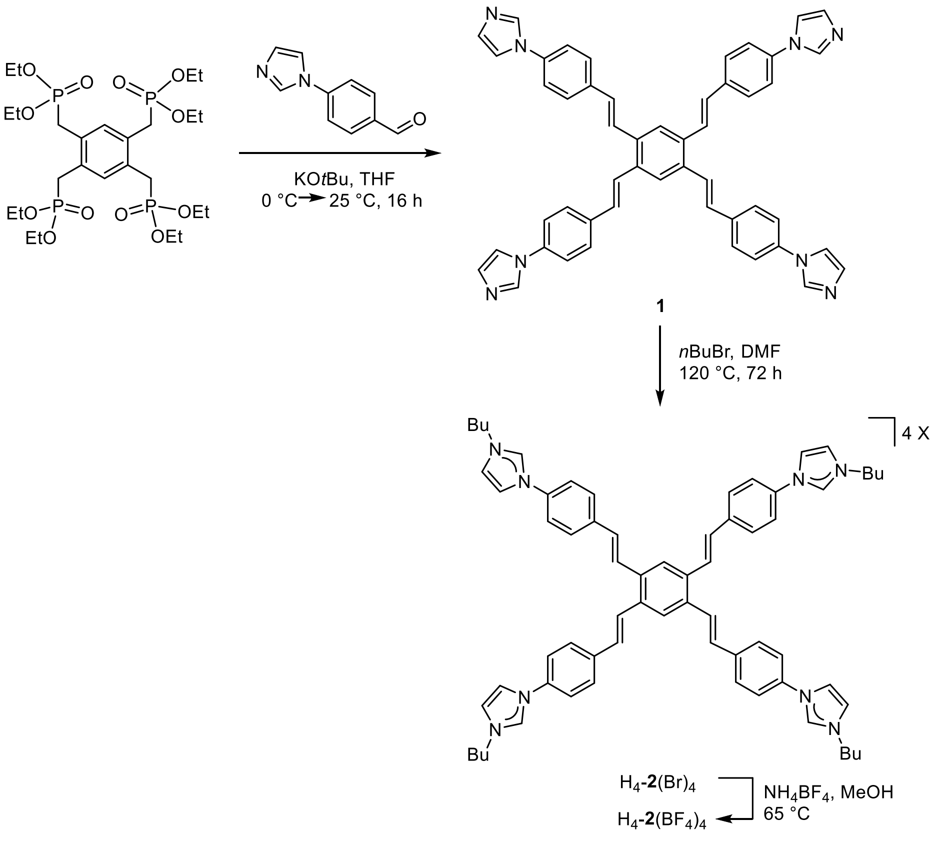

Scheme S1. Synthesis of tetrakisimidazolium salts H$_4$-**2**(X)$_4$ (X = Br, BF$_4$).

2.1. Synthesis of compound 1

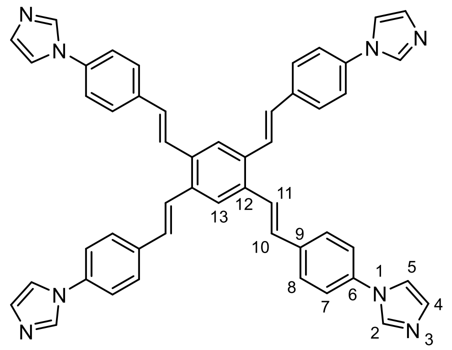

A solution of potassium *tert*-butoxide (2.61 g, 23.3 mmol) in THF (50 mL) was added slowly to a solution of 4-imidazole benzaldehyde (2.00 g, 11.6 mmol) and 1,2,4,5-tetrakis(diethoxyphosphinylmethyl)benzene (1.31 g, 1.93 mmol) in THF (50 mL) at 0 °C. The solution was stirred for 1 h at 0 °C and was then warmed to 25 °C overnight while stirring. Methanol (25 mL) and water (25 mL) were added to the reaction mixture leading to the precipitation of a yellow solid. The solid was isolated by filtration and washed with diethyl ether (3 × 15 mL). The solid was dried *in vacuo* to give compound **1** as a yellow solid. Yield: 1.06 g (1.41 mmol, 73.1%). ^{1}H NMR (400.1 MHz, DMSO-d_6): δ(ppm) = 8.34 (s, 4H, H2), 8.13 (s, 2H, H13), 7.92 (d, $^{3}J_{HH}$ = 8.2 Hz, 8H, H8), 7.84 (d, $^{3}J_{HH}$ = 16.4 Hz, 4H, H11), 7.82 (s, 4H, H5), 7.73 (d, $^{3}J_{HH}$ = 8.2 Hz, 8H, H7), 7.42 (d, $^{3}J_{HH}$ = 16.4 Hz, 4H, H10), 7,14 (s, 4H, H4). ^{13}C{^{1}H} NMR (101 MHz, DMSO-d_6): δ (ppm) = 136.0, 135.8, 135.4, 130.0, 129.9, 128.2, 120.2, 117.7. No 2D spectra could be recorded due to low solubility of **1**. MS (EI, positive ions): *m*/*z* (%) = 750 (10, calcd for [**1**]$^{+}$ 750). Anal. Calcd for **1**·1.5H$_2$O ($C_{50}H_{38}N_8$·1.5 H_2O): C, 77.19; H, 5.31; N, 14.41. Found: C, 77.30; H, 5.15; N, 13.98.

2.2. Synthesis of compound H$_4$-2(Br)$_4$

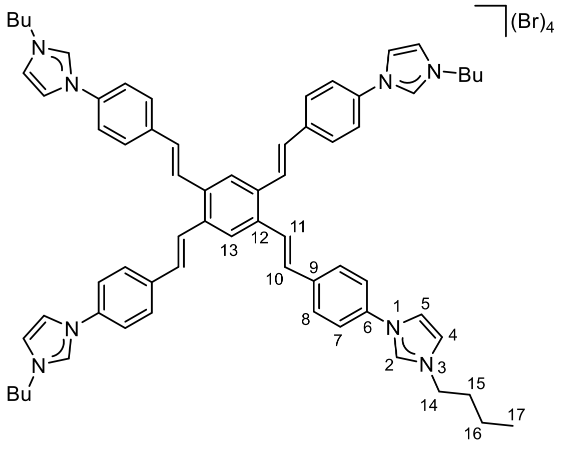

A suspension of 1-bromobutane (6.2 mL, 7.9 g, 57.7 mmol) and **1** (700 mg, 0.93 mmol) in DMF (40 mL) was stirred at 120 °C for 72 h. The suspension was then cooled to ambient temperature and filtered. The solid obtained was washed with DMF (2 × 10 mL), acetone (3 × 15 mL) and diethyl ether (3 × 15 mL). The solid was dried *in vacuo* to give compound H$_4$-**2**(Br)$_4$ as a yellow solid. Yield: 1.03 g (0.79 mmol, 85%). ^{1}H NMR (400.1 MHz, DMSO-d_6): δ (ppm) = 10.07 (s, 4H, H2), 8.44 (s, 4H, H5) 8.24 (s, 2H, H13), 8.12 (s, 4H, H4), 8.11 (d, $^{3}J_{HH}$ = 8.2 Hz, 8H, H8), 7.99 (d, $^{3}J_{HH}$ = 16.1 Hz, 4H, H11), 7.91 (d, $^{3}J_{HH}$ = 8.2 Hz, 8H, H7), 7.59 (d, $^{3}J_{HH}$ = 16.1 Hz, 4H, H10), 4.31 (t, $^{3}J_{HH}$ = 7.2 Hz, 8H, H14), 1.99–1.84 (m, 8H, H15), 1.53–1.24 (m, 8H, H16), 0.95 (t, $^{3}J_{HH}$ = 7.4 Hz, 12H, H17). ^{13}C{^{1}H} NMR (101 MHz, DMSO-d_6): δ(ppm) = 138.5 (C9), 135.1

(C12), 135.1 (C2), 133.7 (C6), 129.8 (C10), 128.4 (C8), 127.0 (C11), 123.8 (C13), 123.3 (C4), 121.7 (C7), 120.8 (C5), 49.1 (C14), 31.1 (C15), 18.8 (C16), 13.3 (C17). HRMS (ESI, positive ions): *m*/*z* (%) = 244.6501 (100, calcd for $[\mathbf{2}]^{4+}$ 244.6509). Anal. Calcd for **2**$(Br)_4 \cdot 4\ H_2O$ ($C_{66}H_{74}N_8Br_4 \cdot 4\ H_2O$): C, 57.82; H, 6.03; N, 8.18. Found: C, 57.87; H, 5.67; N, 8.28%.

2.3 Synthesis of compound H_4-2$(BF_4)_4$

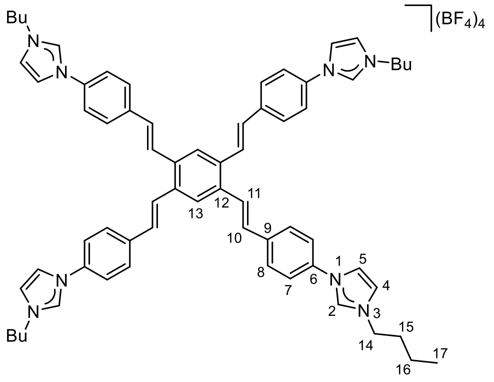

A sample of H_4-**2**$(Br)_4$ (400 mg, 0.308 mmol) was dissolved in warm MeOH (20 mL). To the warm solution was added a solution of NH_4BF_4 (258 mg, 2.46 mmol) in MeOH (5 mL). The resulting suspension was filtered and the obtained solid was washed with MeOH (2 × 5mL). The solid was brought to dryness *in vacuo* to give compound H_4-**2**$(BF_4)_4$ as a yellow solid Yield: 260 mg (0.196 mmol, 64%). ^1H NMR (400.1 MHz, DMSO-d_6): δ (ppm) = 9.85 (s, 4H, H2), 8.39 (s, 4H, H5) 8.19 (s, 2H, H13), 8.08 (s, br, 12H, H4, H8), 7.99 (d, $^3J_{HH}$ = 15.8 Hz, 4H, H11), 7.87 (d, $^3J_{HH}$ = 6.4 Hz, 8H, H7), 7.59 (d, $^3J_{HH}$ = 15.8 Hz, 4H, H10), 4.29 (s, br, 8H, H14), 1.93 (s, br, 8H, H15), 1.39 (s, br, 8H, H16), 0.98 (s, br, 12H, H17). $^{13}C\{^1H\}$ NMR (101 MHz, DMSO-d_6): δ (ppm) = 139.1 (C9), 135.7 (C12), 135.6 (C2), 134.3 (C6), 130.4 (C10), 128.9 (C8), 127.7 (C11), 124.5 (C13), 123.9 (C4), 122.7 (C7), 121.5 (C5), 49.7 (C14), 31.6 (C15), 19.4 (C16), 13.8 (C17). HRMS (ESI, positive ions): *m*/*z* (%) = 244.6501 (100, calcd for $[\mathbf{2}]^{4+}$ 244.6509), 355.2018 (15, calcd for $[\mathbf{2}+BF_4]^{3+}$ 355.2025).

2.4. Synthesis of complex [3]$(BF_4)_4$

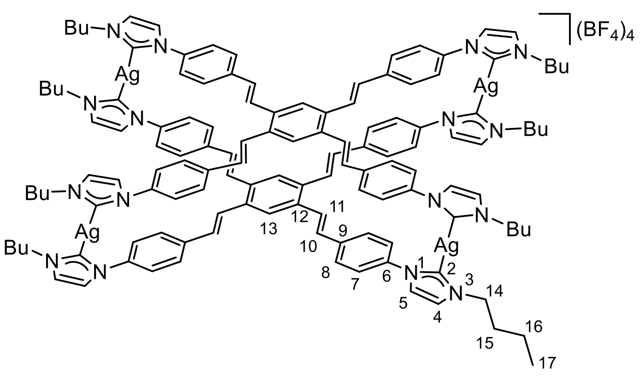

A mixture of H_4-**2**$(Br)_4$ (100 mg, 0.075 mmol) and Ag_2O (37 mg, 0.16 mmol) in methanol (10 mL) was stirred at 55 °C for 16 h. The methanol was then removed under reduced pressure and $AgBF_4$ (30 mg, 0.15 mmol) and acetonitrile (10 mL) were added. The resulting solution was stirred for 12 h at 55 °C. It was subsequently filtered twice trough Celite. The solvent was removed from the filtrate under reduced pressure and the remaining solid was dissolved in CH_2Cl_2 (5 mL). This solution

was again filtered through Celite. The filtrate was brought to dryness *in vacuo* to give compound [**3**](BF_4)$_4$ as a yellow solid. Yield: 90 mg (0.033 mmol, 86%). ^{1}H NMR (400.1 MHz, DMSO-d_6): δ (ppm) = 7.96 (s, 8H, H5), 7.86 (s, 12H, H4, H13), 7.76 (d, $^3J_{HH}$ = 8.2 Hz, 16H, H8), 7.66 (d, $^3J_{HH}$ = 8.2 Hz, 16H, H7), 7.54 (d, $^3J_{HH}$ = 16.0 Hz, 8H, H11), 7.31 (d, $^3J_{HH}$ = 16.0 Hz, 8H, H10), 4.39 (s, 16H, H14), 2.04 –1.88 (m, 16H, H15), 1.56–1.38 (m, 16H, H16), 1.02 (q, $^3J_{HH}$ = 7.2 Hz, 24H, H17). ^{13}C{^{1}H} NMR (101 MHz, DMSO-d_6): δ (ppm) = 178.1 (d, $^1J_{AgC}$ = 149 Hz, C2), 138.4 (C6), 137.2 (C9), 134.2 (C12), 129.2 (C10), 127.6(C8), 126.2 (C11), 123.6 (C13), 123.3 (C4), 123.2 (C7), 122.3 (C5), 51.7 (C14), 33.3 (C15), 19.4 (C16), 13.5 (C17). HRMS (ESI, positive ions): *m*/*z* (%) = 595.4418 (100, calcd for [**3**]$^{4+}$ 595.4422), 822.9297 (15, calcd for [[**3**]+BF_4]$^{3+}$ 822.9242), 1277.8895 (2, calcd for [[**3**]+2(BF_4)]$^{2+}$ 1277.8881).

2.5. Synthesis of complex [4](BF_4)$_4$

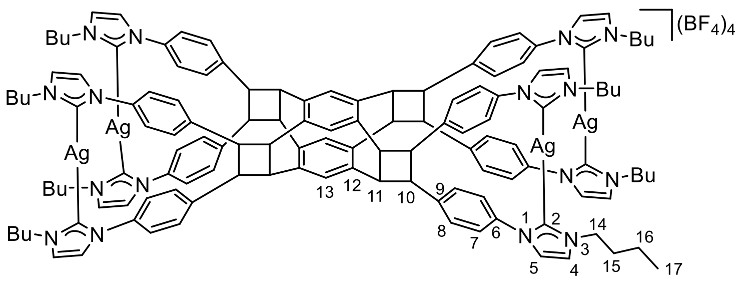

In a quartz Schlenk tube, a solution of [**3**](BF_4)$_4$ (100 mg, 0.037 mmol) in acetonitrile (50 mL) was irradiated with a Philips mercury high-pressure lamp for 16 h at 25 °C. The solution was then filtered through Celite and the filtrate was brought to dryness *in vacuo* to give compound [**4**](BF_4)$_4$ as a brownish solid. The conversion from [**3**](BF_4)$_4$ to [**4**](BF_4)$_4$ was quantitative as judged by NMR spectroscopy. ^{1}H NMR (400.1 MHz, CD_3CN): δ (ppm) = 7.47 (s, br, 20H, H13, H8), 7.37 (d, br, $^3J_{HH}$ = 7.3 Hz, 16H, H4, H5), 7.26 (d, br, $^3J_{HH}$ = 6.4 Hz, 16H, H7), 5.32 (s, br, 8H, H11), 5.18 (s, br, 8H, H10), 4.24 (t, $^3J_{HH}$ = 6.0 Hz, 16H, H14), 1.94–1.87 (m, 16H, H15), 1.48–1.36 (m, 16H, H16), 1.00 (t, $^3J_{HH}$ = 7.3 Hz, 24H, H17). ^{13}C{^{1}H} NMR (101 MHz, CD_3CN): δ (ppm) = 181.6 (C2), 143.2 (C9), 141.4 (C12), 139.4 (C6), 132.2 (C13), 131.2 (br, C8), 125.8 (C7), 123.6 (C5), 123.0 (C4), 52.8 (C14), 48.3 (C11), 44.3 (C10), 34.5 (C15), 20.7 (C16), 14.1 (C17). HRMS (ESI, positive ions): *m*/*z* (%) = 595.4459 (100, calcd for [**4**]$^{4+}$ 595.4422), 822.9297 (80, calcd for [[**4**]+BF_4]$^{3+}$ 822.9242), 1277.8979 (10, calcd for [[**4**]+2(BF_4)]$^{2+}$ 1277.8881).

2.6. Synthesis of compound H_8-5$(BF_4)_8$

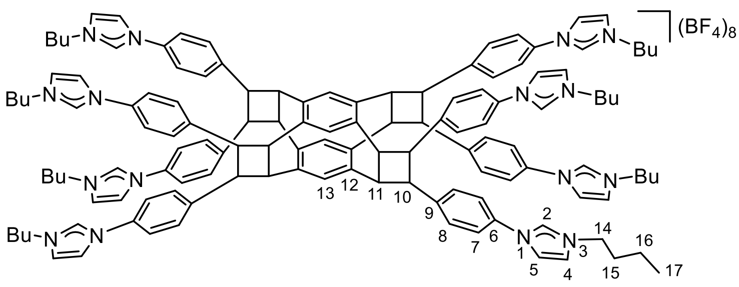

A solution of [**4**]$(BF_4)_4$ (100 mg, 0.037 mmol) in methanol (20 mL) and NH_4Cl (15.8 mg, 0.30 mmol) was stirred for 2 h at 25 °C. Immediately a white solid (AgCl) precipitated. The suspension was filtered through Celite and NH_4BF_4 (34.6 mg, 0.33 mmol) was added. The resulting solution was stirred at 25 °C for 2 h. After this period a brownish solid had precipitated which was isolated by filtration. The solid was dissolved in DMSO (2 mL) and water (4 mL) was added resulting in the precipitation of a brown solid. The solid was isolated by filtration, washed with diethyl ether (3 × 5 mL) and was brought to dryness *in vacuo* to give compound H_8-**5**$(BF_4)_8$ as a brownish solid. Yield: 73 mg (0.028 mmol, 76%). ^{1}H NMR (400.1 MHz, DMSO-d_6): δ (ppm) = 9.70 (s, 8H, H2), 8.24 (s, 8H, H5), 8.04 (s, 8H, H4), 7.79–7.57 (m, 32H, H7, H8), 7.24 (s, 4H, H13), 5.19 (s, 8H, H11), 5.10 (s, 8H, H10), 4.23 (t, $^3J_{HH}$ = 6.5 Hz, 16H, H14), 1.92–1.81 (m, 16H, H15), 1.38–1.27 (m, 16H, H16), 0.93 (t, $^3J_{HH}$ = 6.1 Hz, 24H, H17). ^{13}C{^{1}H} NMR (101 MHz, DMSO-d_6): δ (ppm) = 143.2 (C9), 140.0 (C12), 134.7 (C2), 132.5 (C6), 130.3 (C8, C13), 123.4 (C4), 120.7 (C5, C7), 49.1 (C14), 48.6 (C11), 41.7 (C10), 31.0 (C15), 18.8 (C16), 13.2 (C17). HRMS (ESI, positive ions): *m*/*z* (%) = 244.7773 (1, calcd for [H_8-**5**]$^{8+}$ 244.7763), 292.1748 (40, calcd for [H_8-**5**+BF_4]$^{7+}$ 292.1734), 355.3715 (100, calcd for [H_8-**5**+2(BF_4)]$^{6+}$ 355.3697), 443.8467 (40, calcd for [H_8-**5**+3(BF_4)]$^{5+}$ 443.8444), 576.3097 (20, calcd for [H_8-**5**+4(BF_4)]$^{4+}$ 576.3061).

2.7. Synthesis of complex [6]$(BF_4)_4$

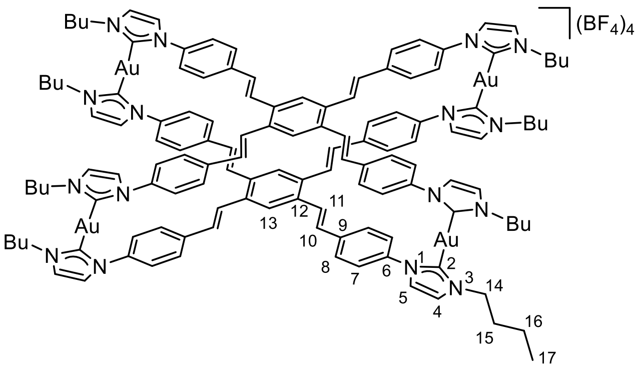

A mixture of [**3**]$(BF_4)_4$ (100 mg, 0.037 mmol) and [AuCl(tht)] (52 mg, 0.16 mmol) in acetonitrile (10 mL) was stirred for 16 h at 55 °C. The solution was then filtered through Celite and the solvent was removed from the filtrate. The solid residue was dissolved in CH_2Cl_2 (10 mL) and the solution was filtered through Celite. Then Et_2O was added to the filtrate leading to precipitation of a yellow solid. The solid was isolated by filtration, washed with diethyl ether (3 × 5 mL) and brought to dryness *in vacuo* to give compound [**6**]$(BF_4)_4$ as a yellow solid. Yield:

80 mg (0.026 mmol, 70%). ^{1}H NMR (400.1 MHz, CD_3CN): δ (ppm) = 7.95 (d, $^3J_{HH}$ = 1.6 Hz, 8H, H5), 7.91 (d, $^3J_{HH}$ = 1.6 Hz, 8H, H4), 7.87 (s, 4H, H13), 7.74 (d, $^3J_{HH}$ = 8.4 Hz, 16H, H7), 7.64 (d, $^3J_{HH}$ = 8.4 Hz, 16H, H8), 7.56 (d, $^3J_{HH}$ = 16.1 Hz, 8H, H11), 7.30 (d, $^3J_{HH}$ = 16.1 Hz, 8H, H10), 4.44 (t, $^3J_{HH}$ = 6.7 Hz, 16H, H14), 2.05–1.94 (m, 16H, H15), 1.50–1.38 (m, 16H, H16), 1.02 (t, $^3J_{HH}$ = 7.2 Hz, 24H, H17). $^{13}C\{^1H\}$ NMR (101 MHz, CD_3CN): δ (ppm) = 180.3 (C2), 137.5 (C9), 137.3 (C6), 134.2 (C12), 129.3 (C10), 127.4 (C8), 126.3 (C11), 124.1 (C7), 123.5 (C13), 123.3 (C4), 122.9 (C5), 51.0 (C14), 33.0 (C15), 19.3 (C16), 13.51 (C17). HRMS (ESI, positive ions): *m/z* (%) = 684.5022 (100, calcd for $[\mathbf{6}]^{4+}$ 684.5035), 941.6711 (80, calcd for $[[\mathbf{6}]+BF_4]^{3+}$ 941.6726), 1456.0096 (10, calcd for $[[\mathbf{6}]+2(BF_4)]^{2+}$ 1456.0109).

2.8. Synthesis of complex [7]$(BF_4)_4$

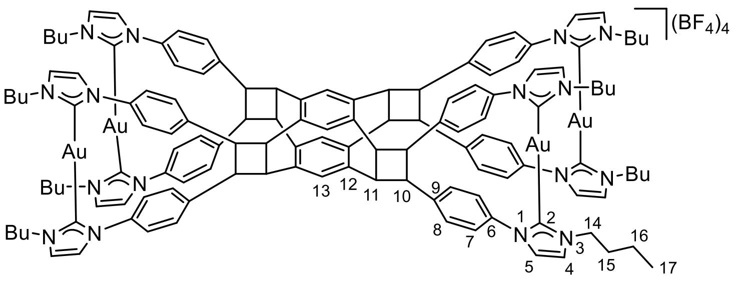

Route A. A solution of [**6**]$(BF_4)_4$ (120 mg, 0.039 mmol) in acetonitrile (50 mL) in a quartz Schlenk flask was irradiated with a Philips mercury high-pressure lamp for 16 h at 25 °C. The solution was then filtered through Celite and the filtrate was brought to dryness *in vacuo* to give compound [**7**]$(BF_4)_4$ as a brown solid. The conversion of [**6**]$(BF_4)_4$ to [**7**]$(BF_4)_4$ was quantitative as judged by NMR spectroscopy.

Route B. A mixture of [**4**]$(BF_4)_4$ (80 mg, 0.029 mmol) and [AuCl(tht)] (38 mg, 0.12 mmol) in acetonitrile (10 mL) was stirred at 55 °C for 16 h. The solution was filtered through Celite and the solvent was removed from the filtrate *in vacuo*. The solid obtained was dissolved in CH_2Cl_2 (10 mL) and filtered through Celite. Then Et_2O was added to the filtrate which caused the precipitation of a yellow solid. The solid was isolated by filtration, washed with diethyl ether (3 × 5 mL) and brought to dryness *in vacuo* to give compound [**7**]$(BF_4)_4$ as a brown solid. Yield: 52 mg (17 mmol, 59%). ^{1}H NMR (400.1 MHz, CD_3CN): δ (ppm) = 7.95–7.16 (m, 52H, H4, H5, H7, H8, H13), 5.34 (d, $^3J_{HH}$ = 4.7 Hz, 8H, H11), 5.20 (d, $^3J_{HH}$ = 4.7 Hz, 8H, H10), 4.41–4.28 (m, 16H, H14), 1.95–1.92 (m, 16H, H15), 1.51–1.35 (m, 16H, H16), 1.05–0.96 (m, 24H, H17). $^{13}C\{^1H\}$ NMR (101 MHz, CD_3CN): δ (ppm) = 184.1 (C2), 143.6 (C9), 141.4 (C12), 138.3 (C6), 132.6 (C8’), 131.6 (C13), 129.7 (C8), 126.3 (C7), 126.2 (C7’), 124.1 (C5), 123.1 (C4), 52.1 (C14), 48.1 (C11), 44.3 (C10), 34.3 (C15), 20.7 (C16), 14.1 (C17). HRMS (ESI, positive ions): *m/z* (%) = 684.5034 (100, calcd for $[\mathbf{7}]^{4+}$ 684.5035).

3. NMR spectra of new compounds

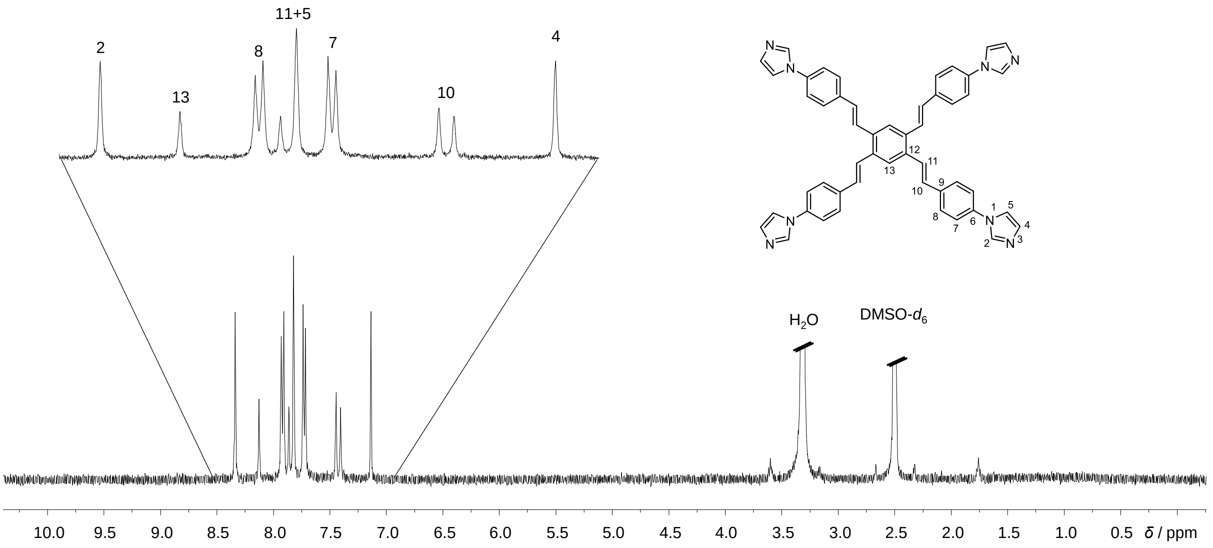

Figure S1. ^{1}H NMR (400.1 Hz) of **1** in DMSO-d_6.

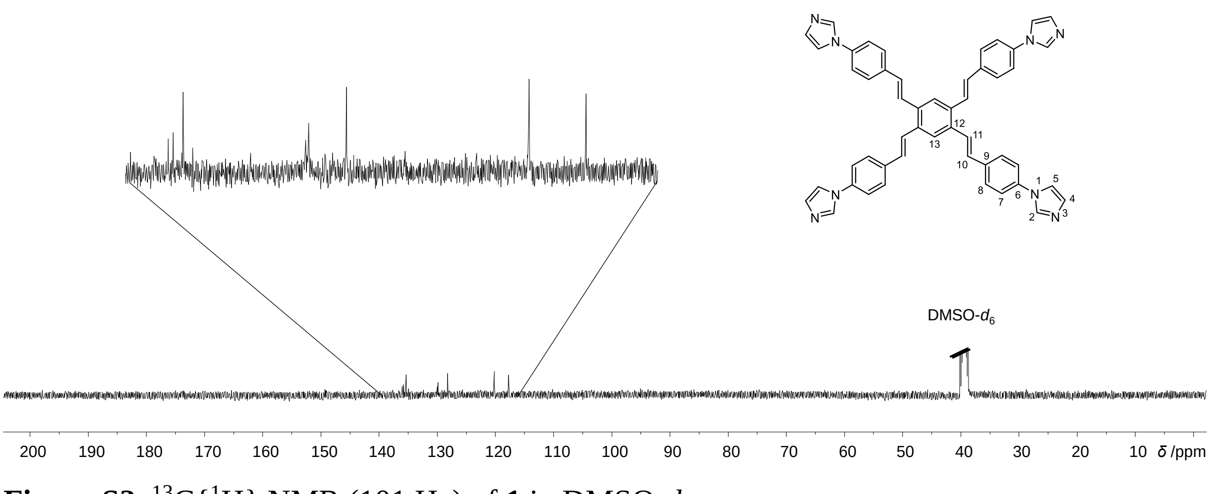

Figure S2. $^{13}C\{^{1}H\}$ NMR (101 Hz) of **1** in DMSO-d_6.

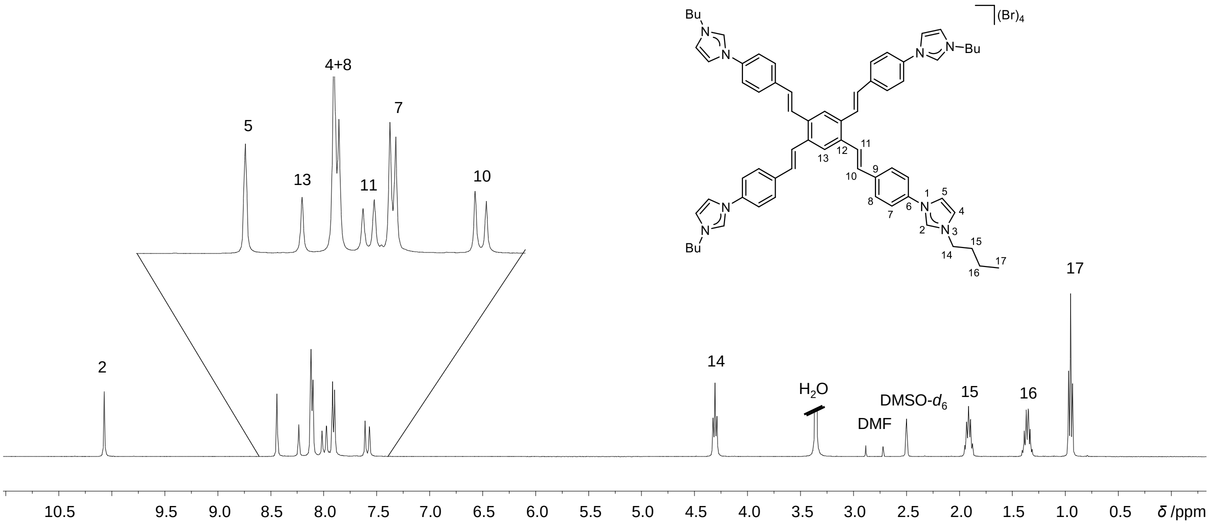

Figure S3. ^{1}H NMR (400.1 Hz) of H_4-**2**$(Br)_4$ in DMSO-d_6.

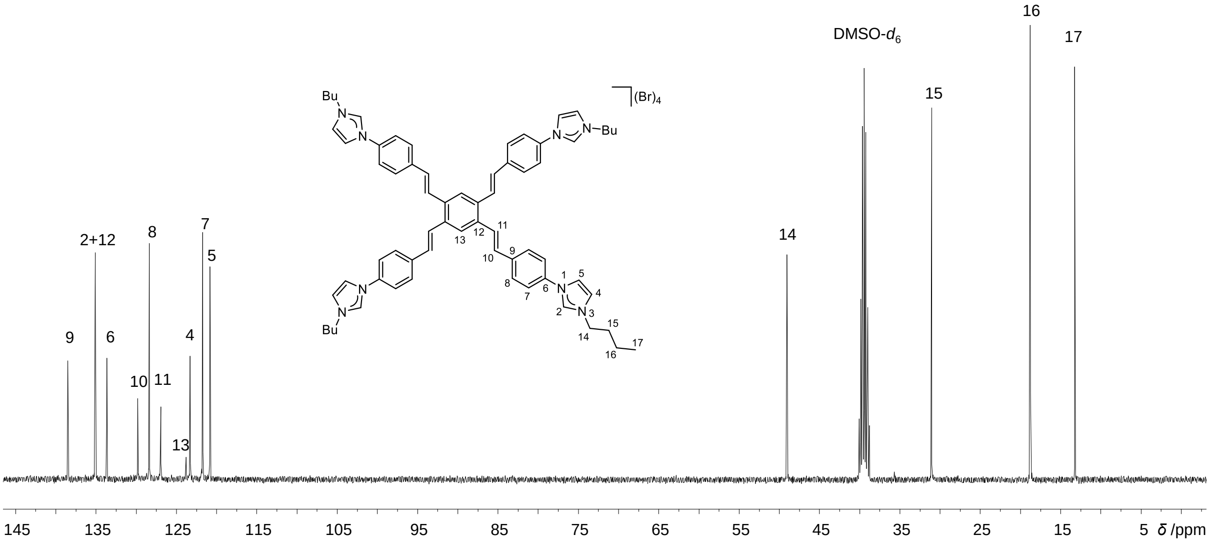

Figure S4. ^{13}C{^{1}H} NMR (101 Hz) of H_4-**2**$(Br)_4$ in DMSO-d_6.

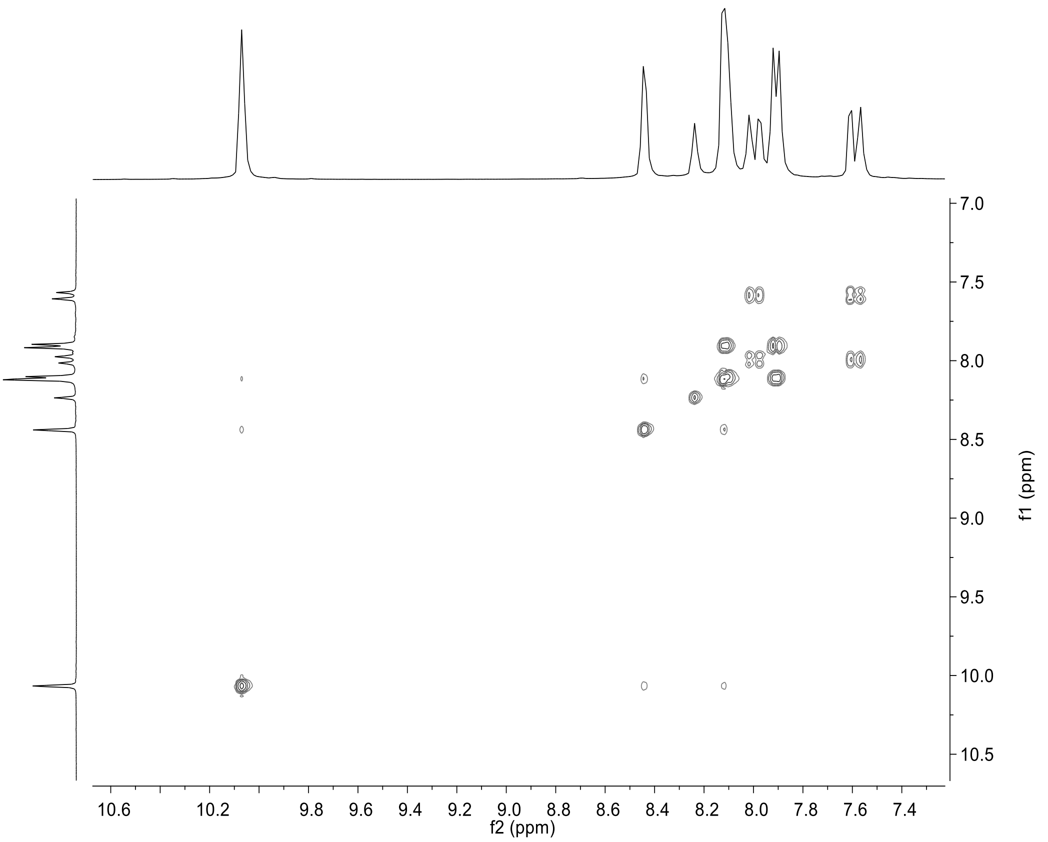

Figure S5. $^{1}H^{1}H$ COSY spectrum (400 MHz) of H_4-**2**$(Br)_4$ in DMSO-d_6.

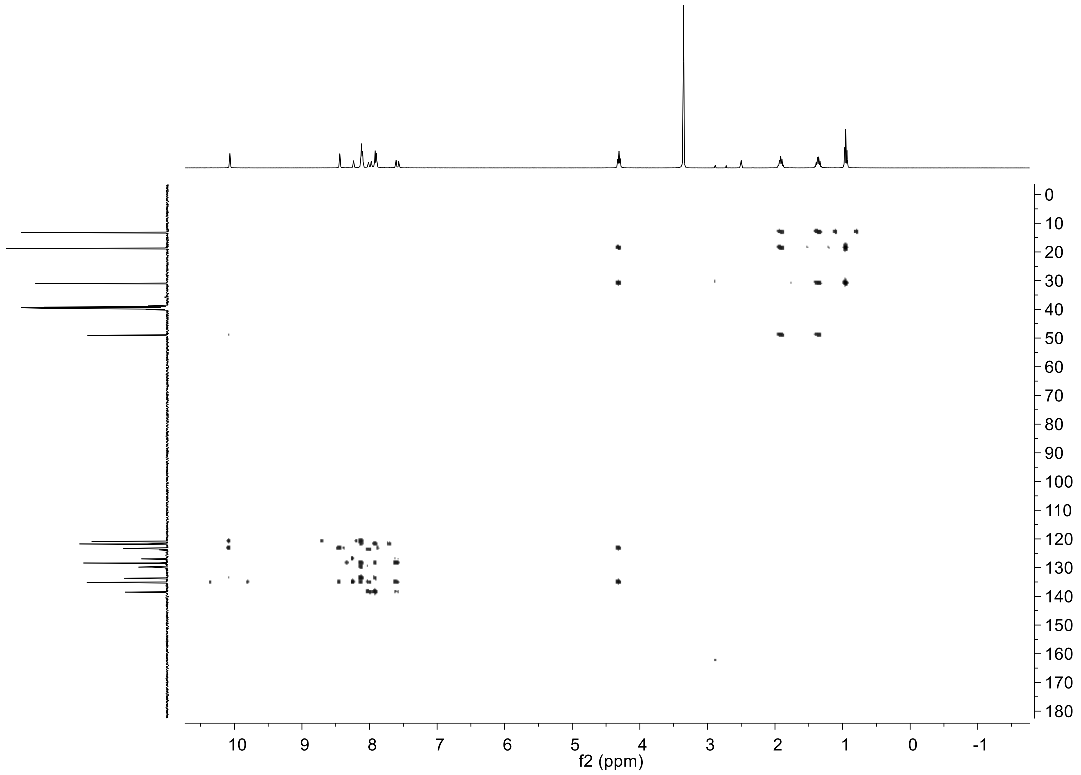

Figure S6. $^{1}H^{13}C$ HMBC spectrum (400 MHz, 101 MHz) of H_4-**2**$(Br)_4$ in DMSO-d_6.

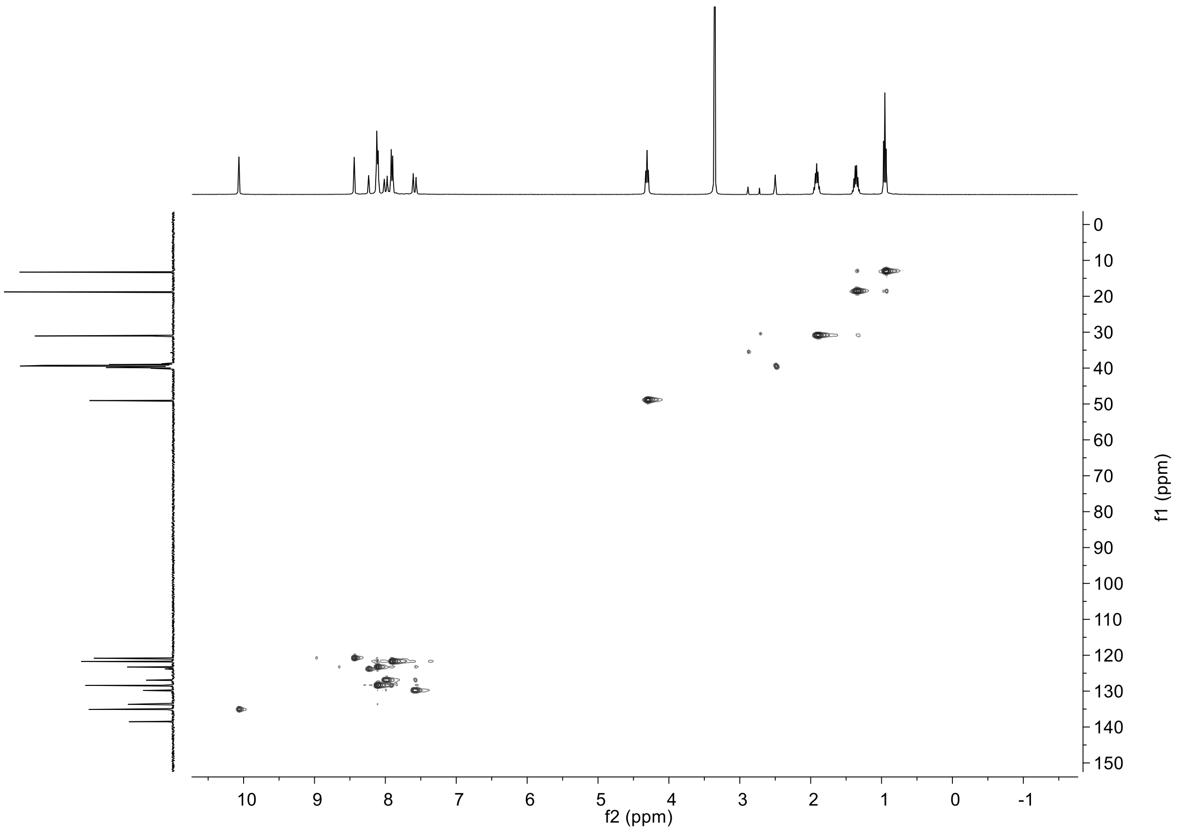

Figure S7. $^{1}H^{13}C$ HSQC spectrum (400 MHz, 101 MHz) of H_4-**2**$(Br)_4$ in DMSO-d_6.

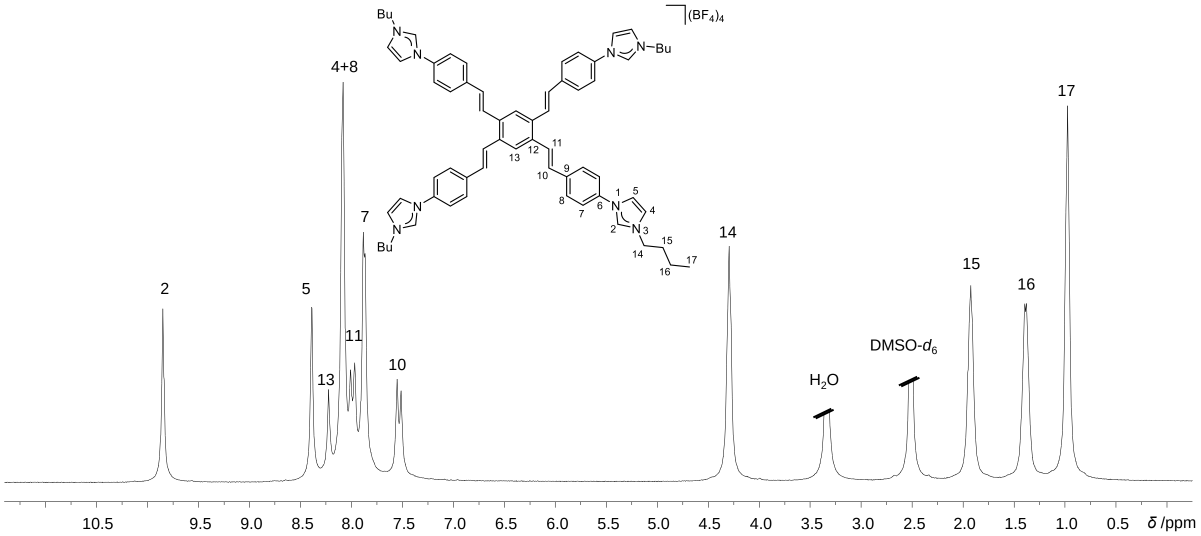

Figure S8. ^{1}H NMR (400.1 Hz) of H_4-**2**$(BF_4)_4$ in DMSO-d_6.

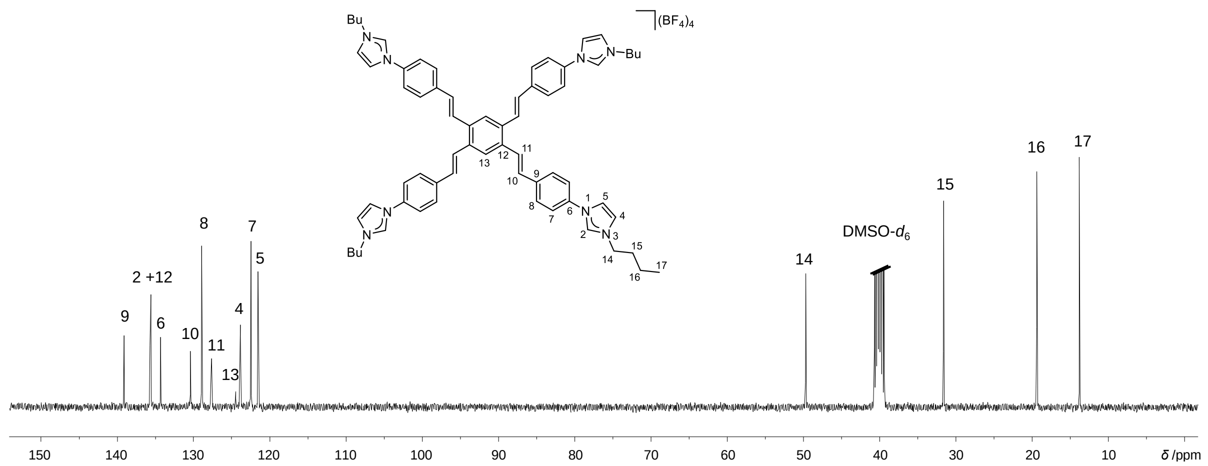

Figure S9. $^{13}C\{^{1}H\}$ NMR (101 Hz) of H_4-**2**$(BF_4)_4$ in DMSO-d_6.

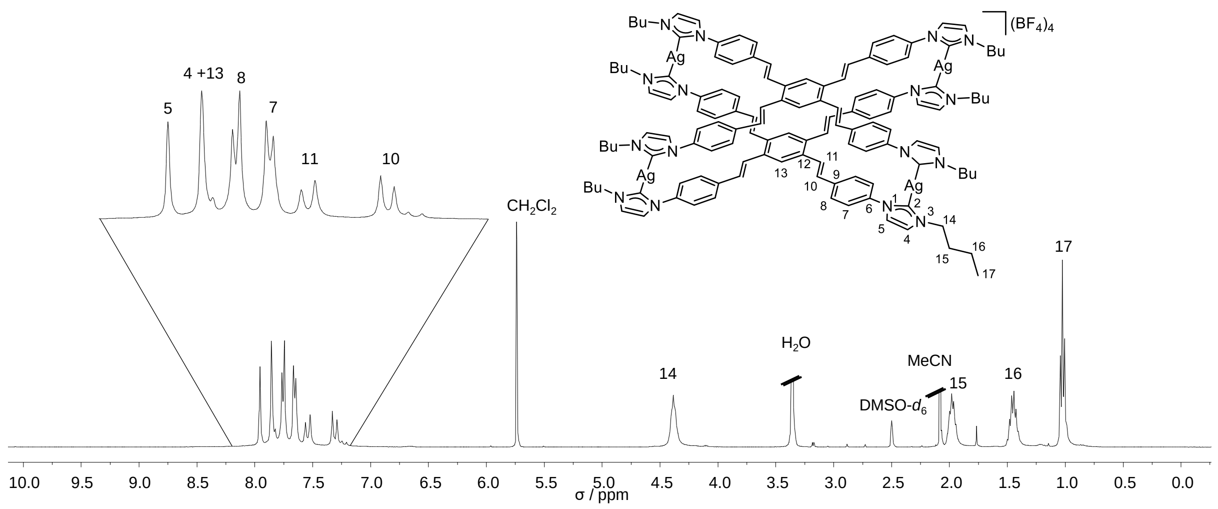

Figure S10. ^{1}H NMR (400.1 Hz) of [**3**]$(BF_4)_4$ in DMSO-d_6.

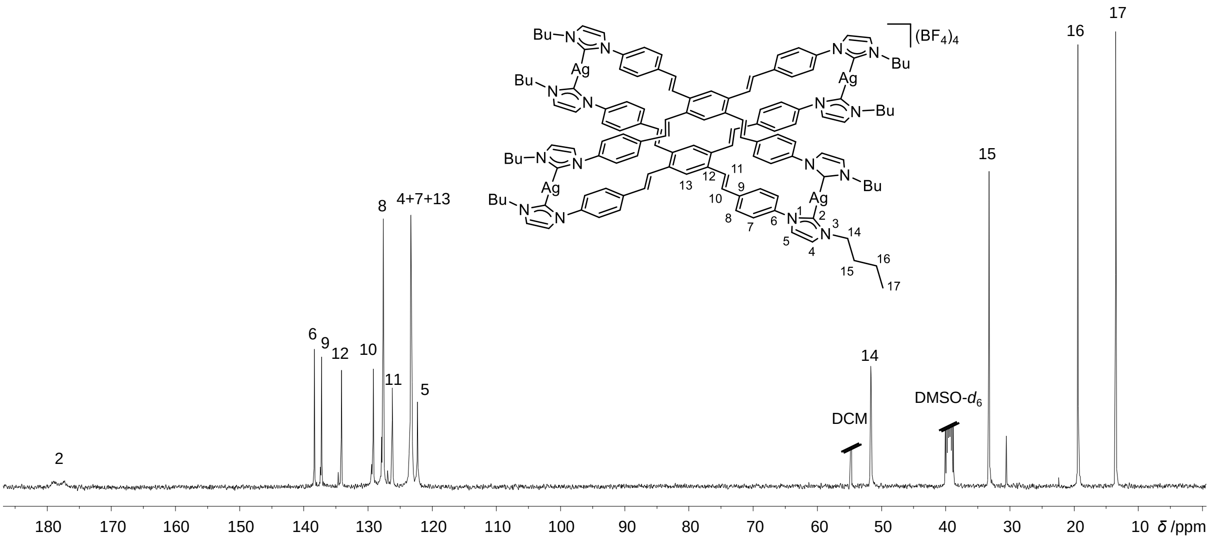

Figure S11. $^{13}C\{^{1}H\}$ NMR (101 Hz) of [**3**]$(BF_4)_4$ in DMSO-d_6.

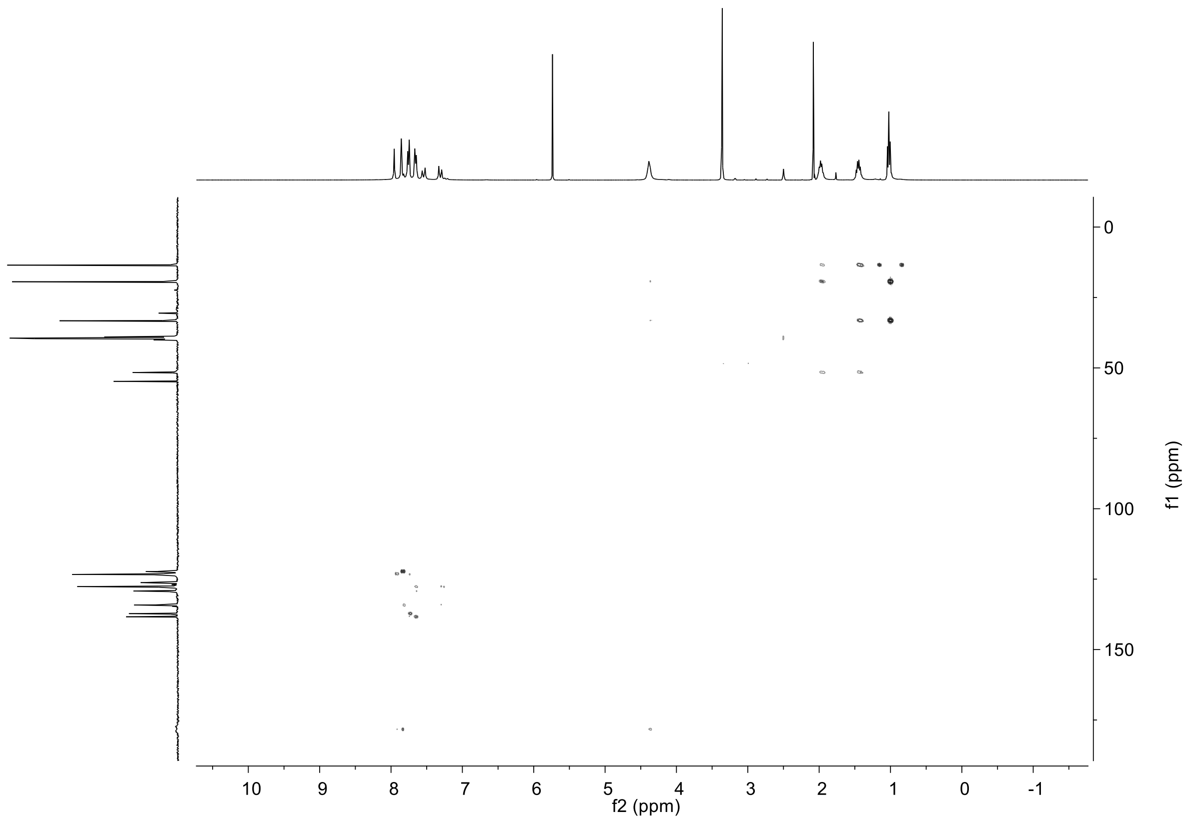

Figure S12. $^{1}H^{13}C$ HMBC spectrum (400 MHz, 101 MHz) of [**3**]$(BF_4)_4$ in DMSO-d_6.

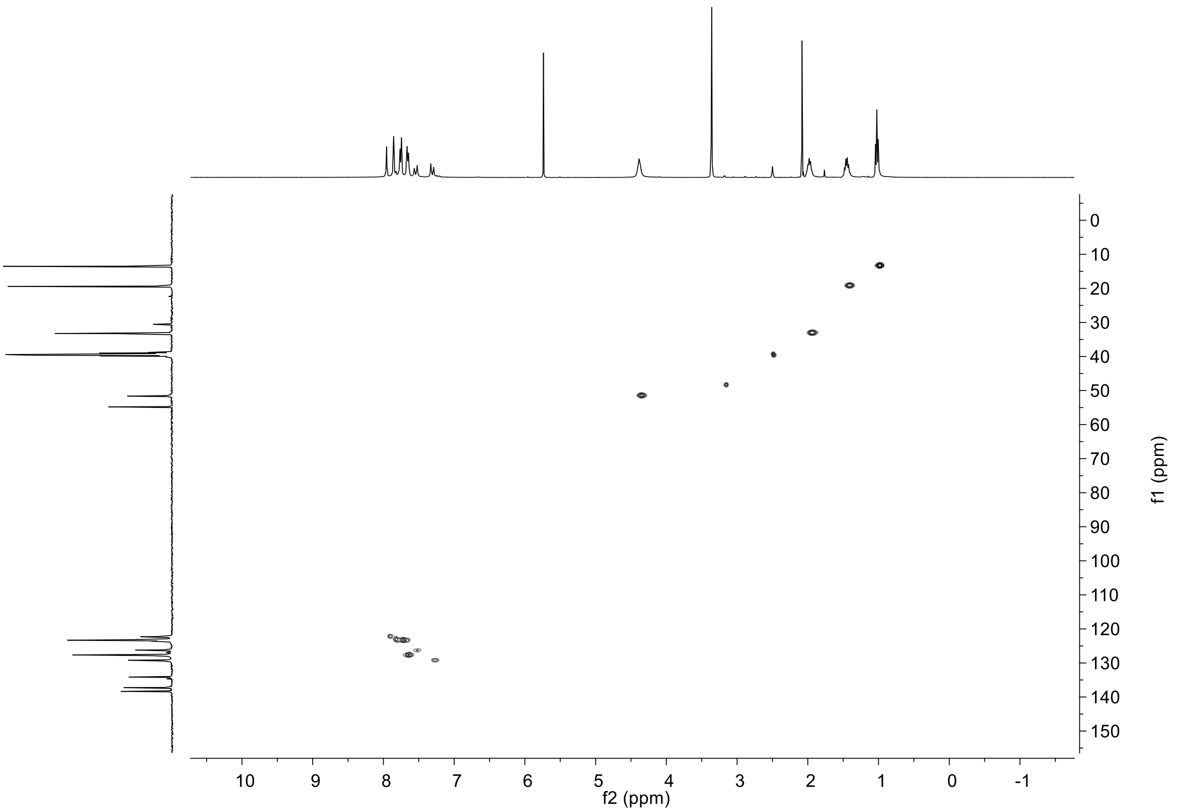

Figure S13. $^{1}H^{13}C$ HSQC spectrum (400 MHz, 101 MHz) of [**3**]$(BF_4)_4$ in DMSO-d_6.

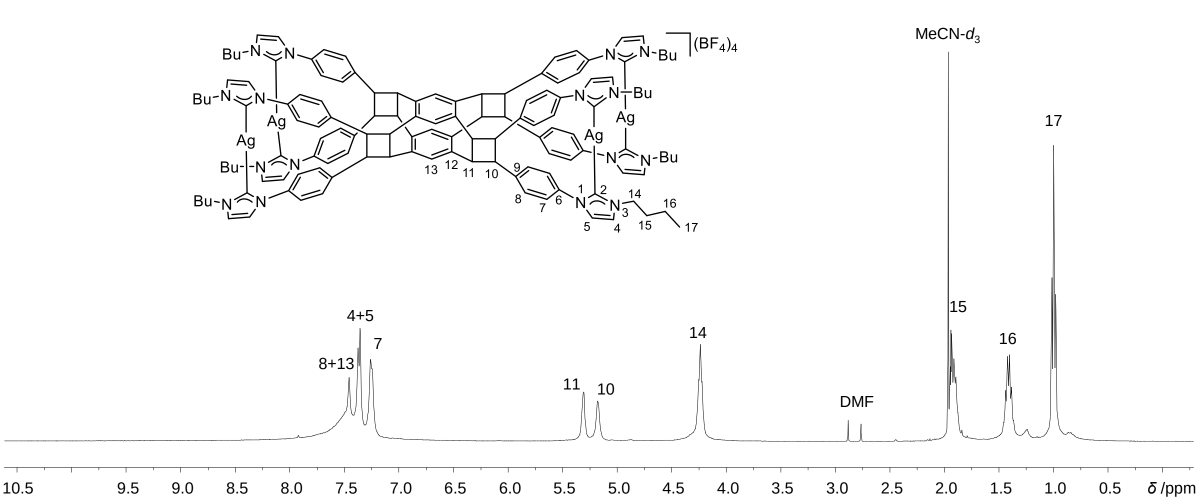

Figure S14. ^{1}H NMR (400.1 Hz) of [**4**]$(BF_4)_4$ in MeCN-d_3.

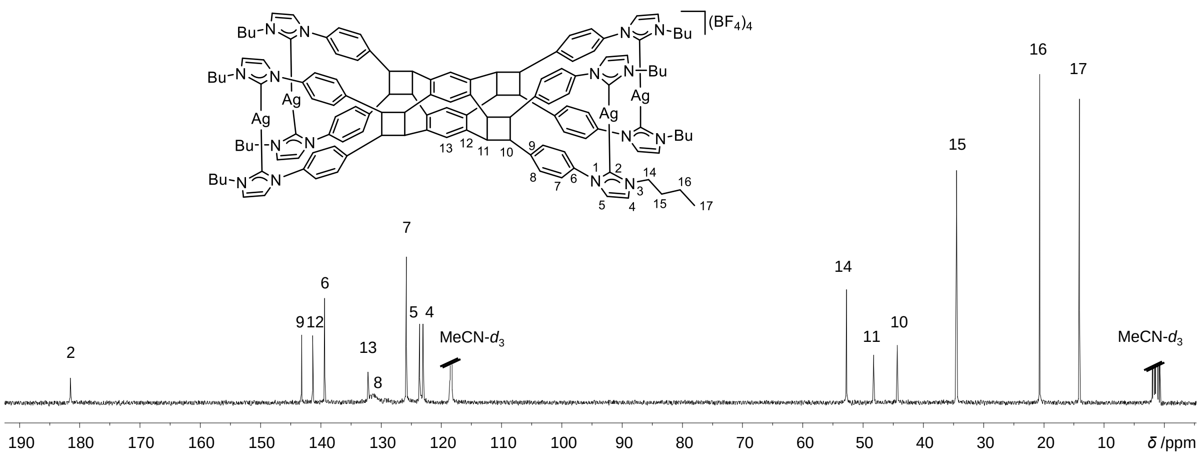

Figure S15. $^{13}C\{^{1}H\}$ NMR (101 Hz) of [**4**]$(BF_4)_4$ in MeCN-d_3.

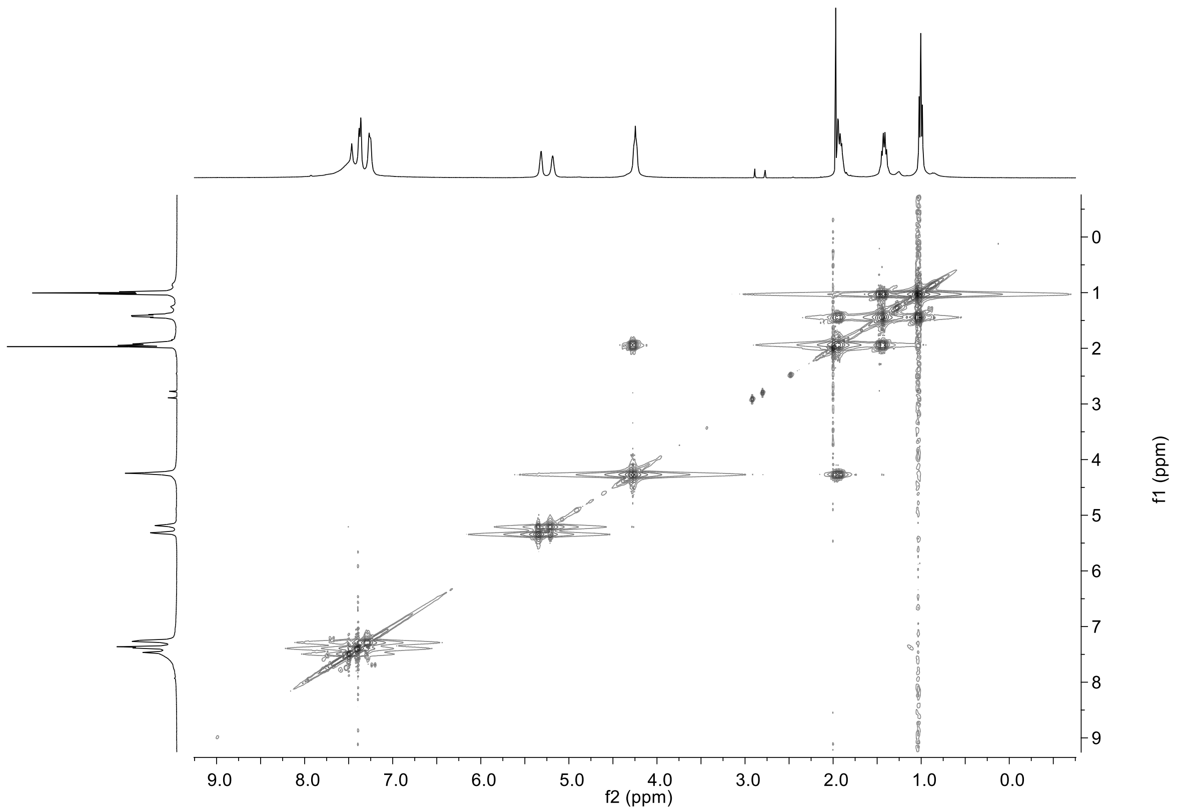

Figure S16. $^{1}H^{1}H$ COSY spectrum (400 MHz) of [**4**]$(BF_4)_4$ in DMSO-d_6.

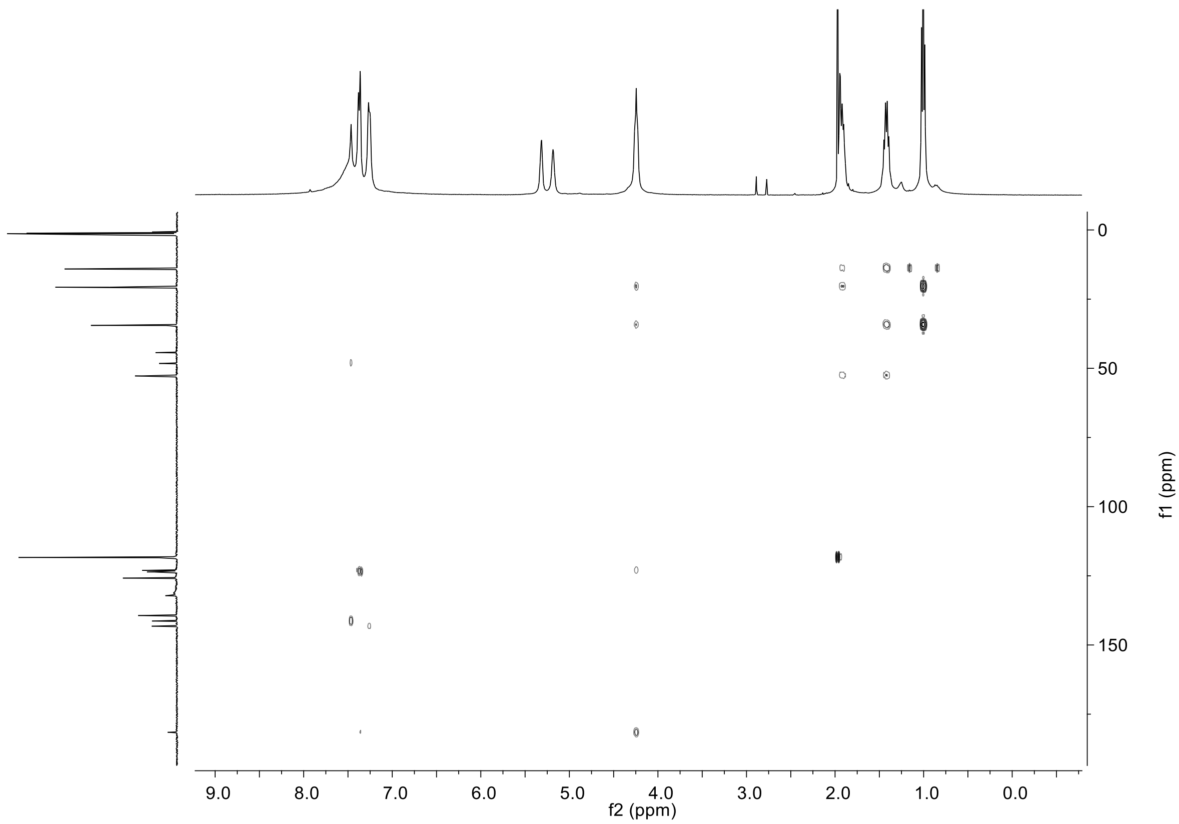

Figure S17. $^{1}H^{13}C$ HMBC spectrum (400 MHz, 101 MHz) of [**4**]$(BF_4)_4$ in DMSO-d_6.

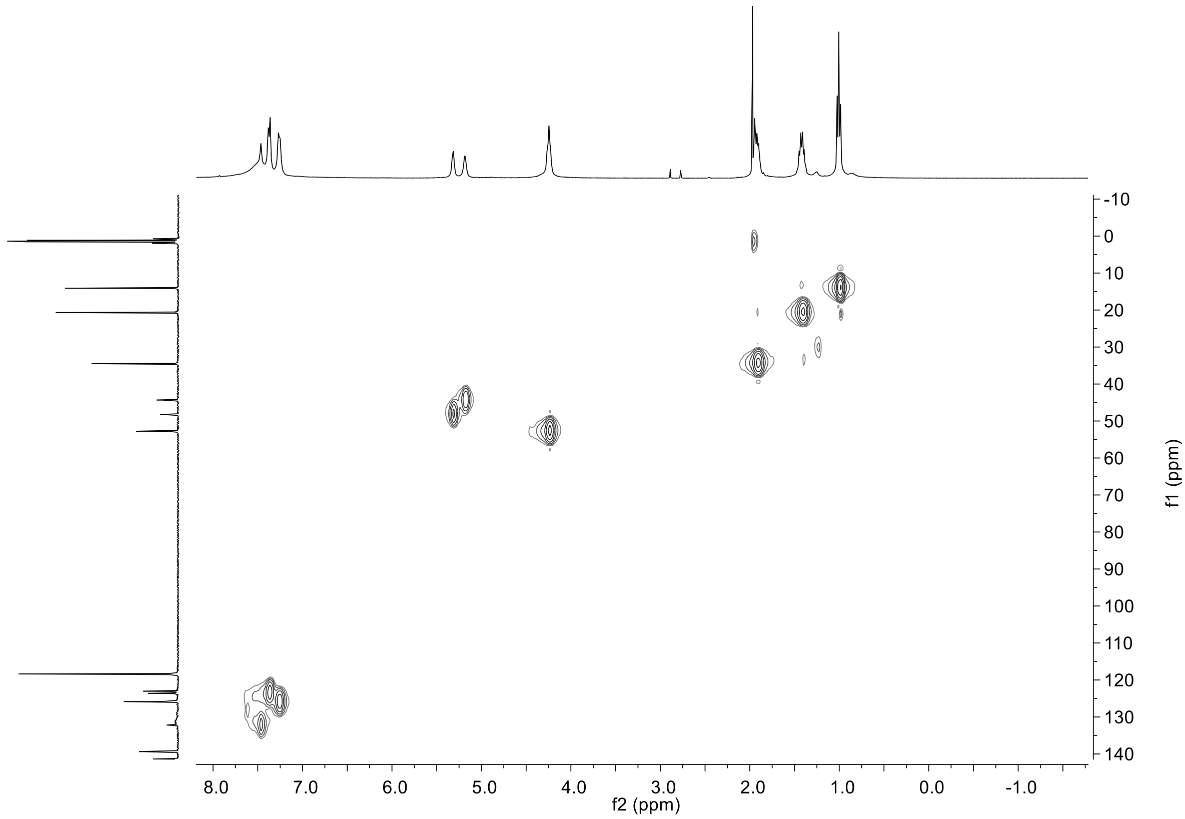

Figure S18. $^{1}H^{13}C$ HSQC spectrum (400 MHz, 101 MHz) of [**4**]$(BF_4)_4$ in DMSO-d_6.

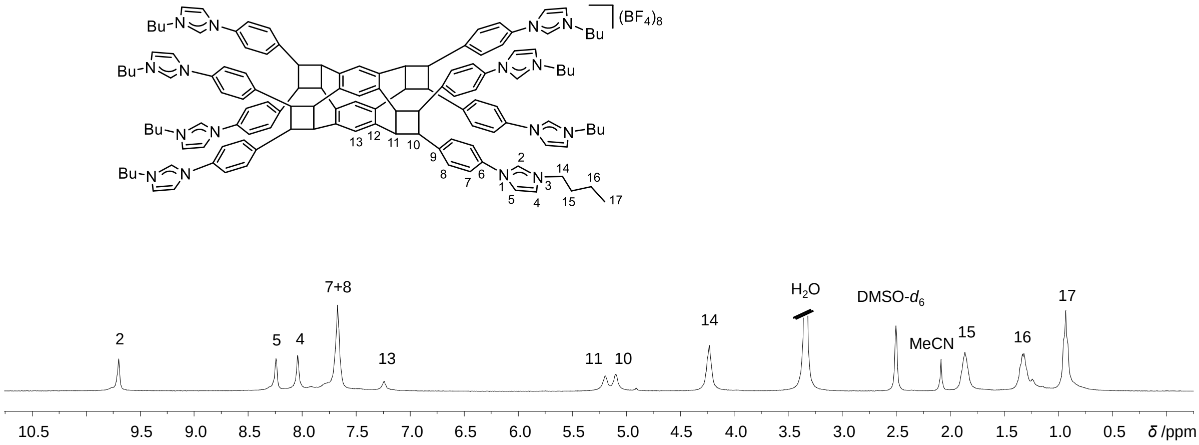

Figure S19. ^{1}H NMR (400.1 Hz) of H_8-**5**$(BF_4)_8$ in DMSO-d_6.

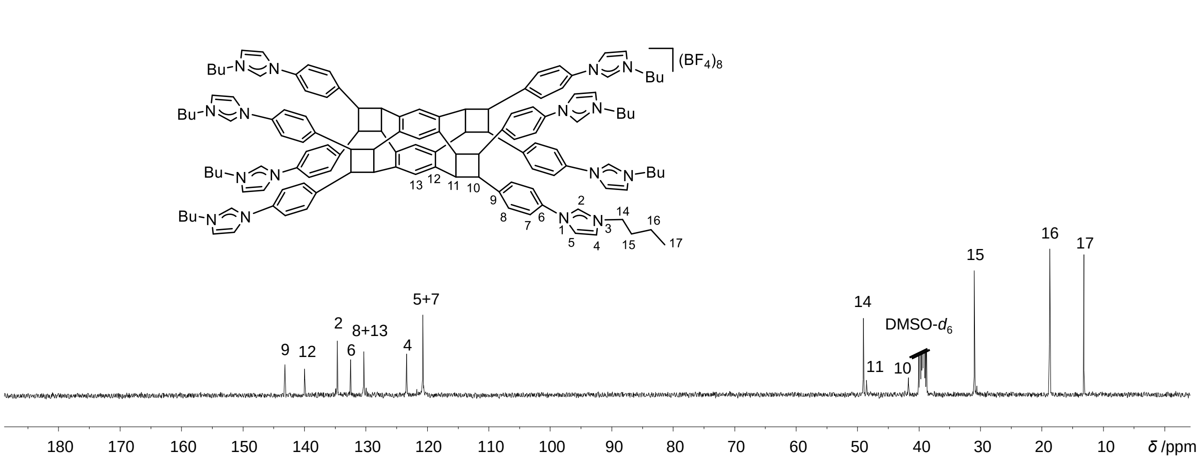

Figure S20. $^{13}C\{^{1}H\}$ NMR (101 Hz) of H_8-**5**$(BF_4)_8$ in DMSO-d_6.

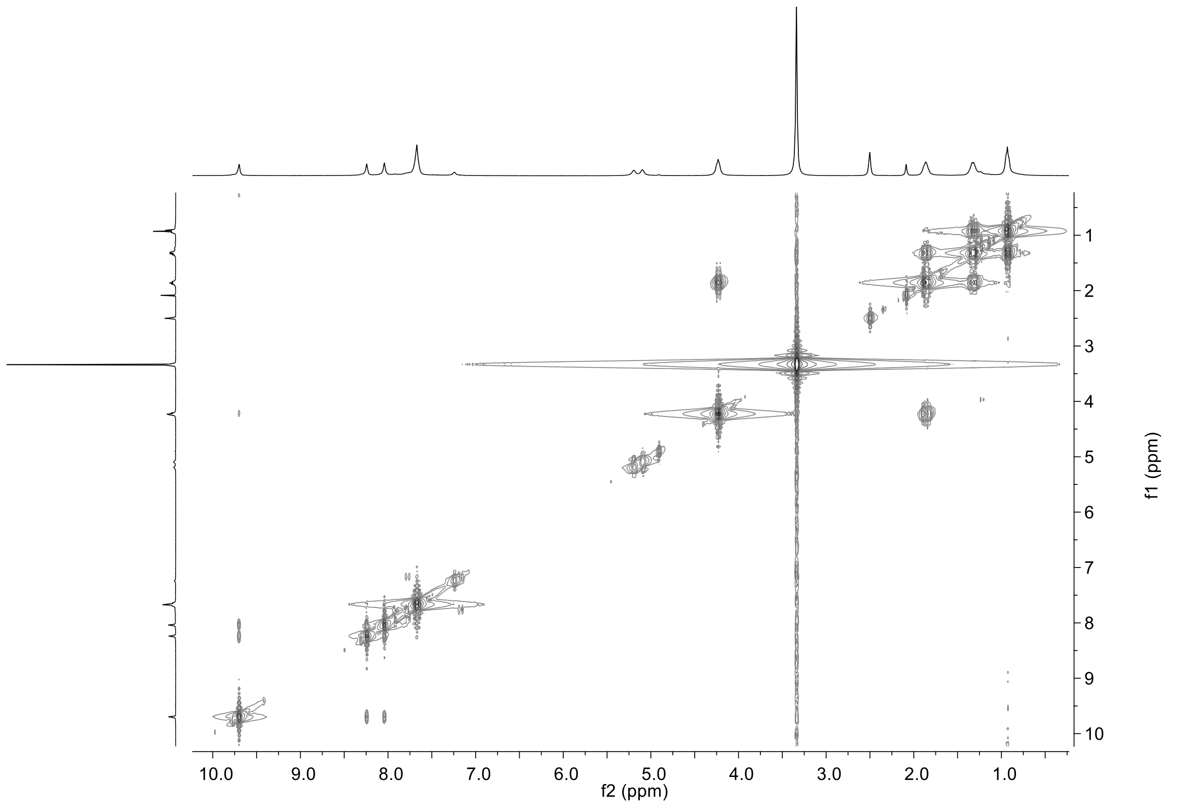

Figure S21. $^{1}H^{1}H$ COSY spectrum (400 MHz) of H_8-**5**$(BF_4)_8$ in DMSO-d_6.

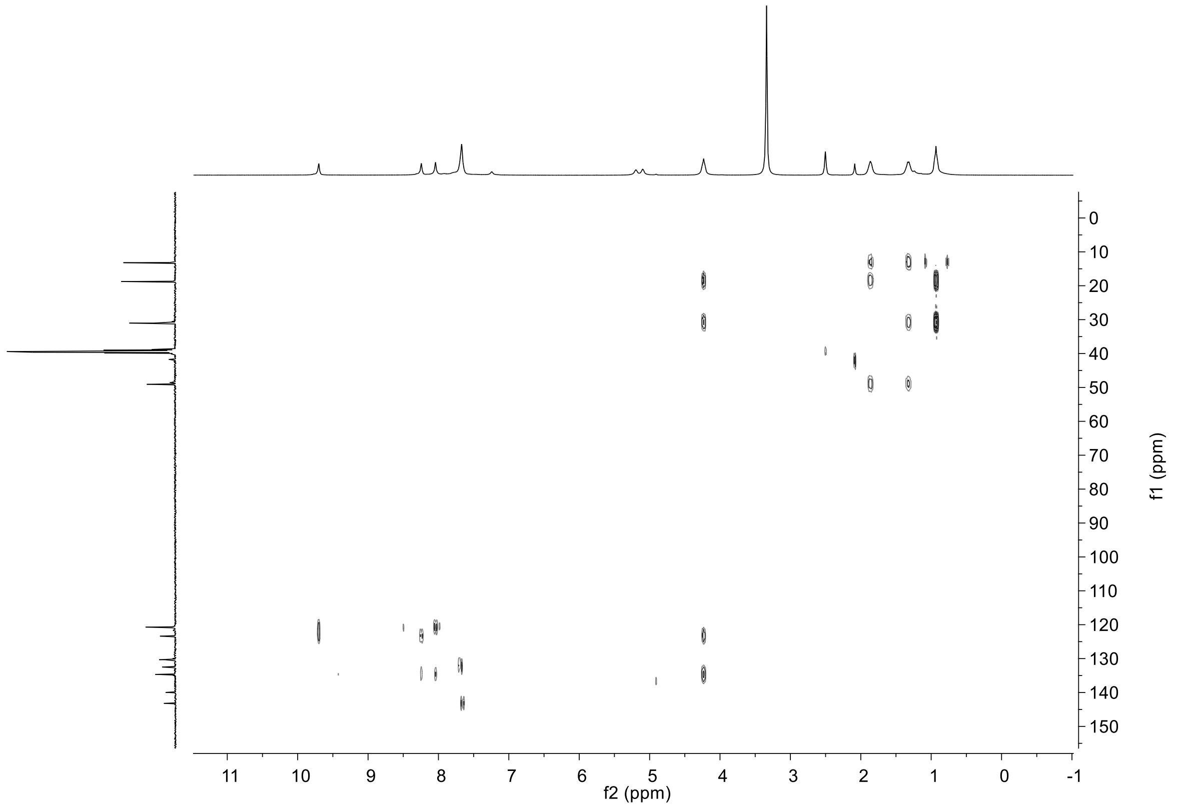

Figure S22. $^{1}H^{13}C$ HMBC spectrum (400 MHz, 101 MHz) of H_8-**5**$(BF_4)_8$ in DMSO-d_6.

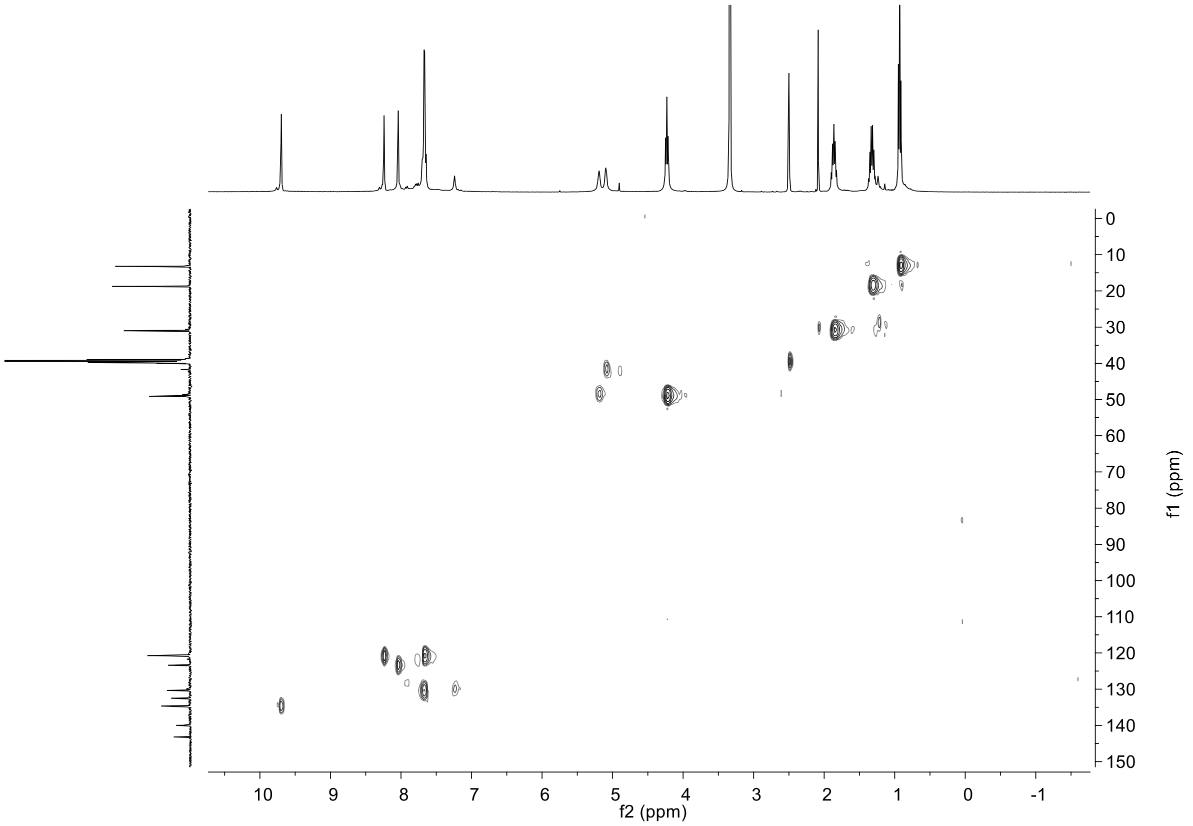

Figure S23. ^{1}H^{13}C HSQC spectrum (400 MHz, 101 MHz) of H_8-**5**$(BF_4)_8$ in DMSO-d_6.

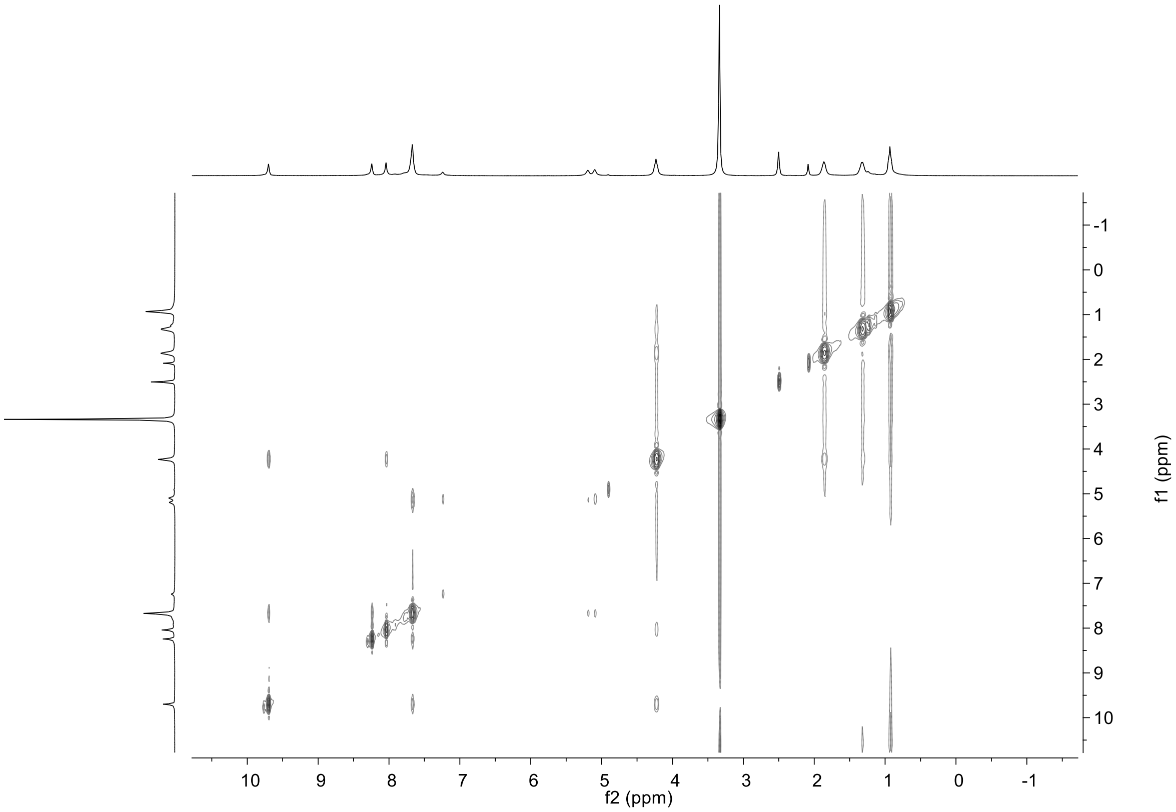

Figure S24. ^{1}H^{1}H ROESY spectrum (400 MHz) of H_8-**5**$(BF_4)_8$ in DMSO-d_6.

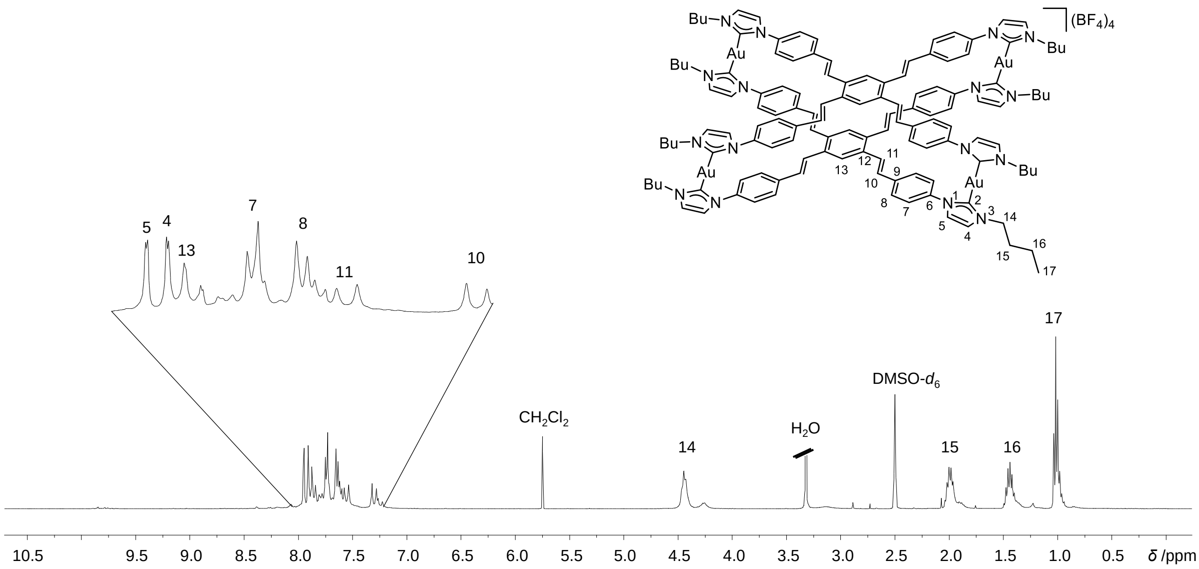

Figure S25. ^{1}H NMR (400.1 Hz) of [**6**]**(**BF_4)**4** in DMSO-*d*6.

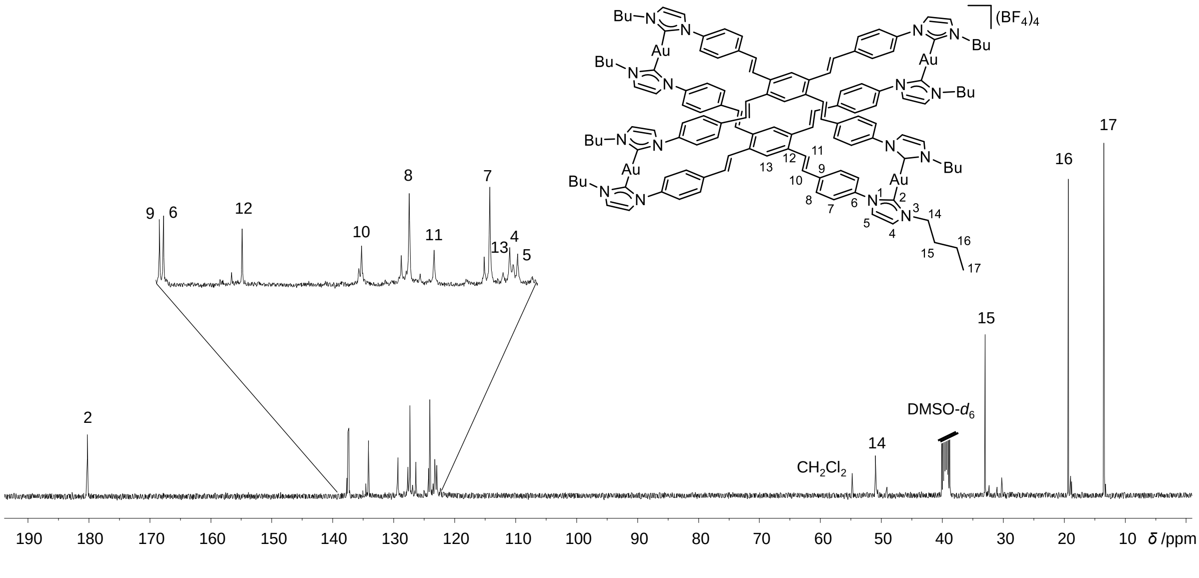

Figure S26. $^{13}C\{^{1}H\}$ NMR (101 Hz) of [**6**](BF_4)$_4$ in DMSO-*d*6.

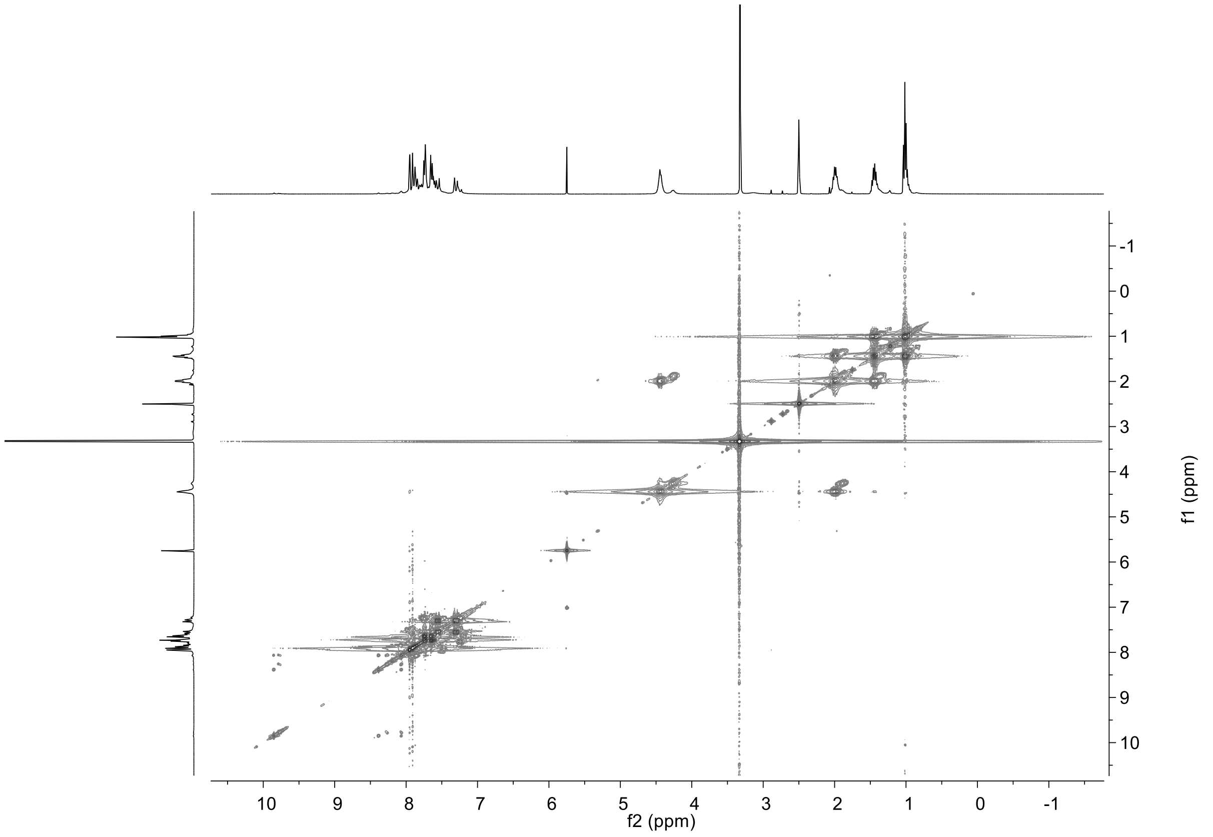

Figure S27. $^{1}H^{1}H$ COSY spectrum (400 MHz) of [**6**](BF_4)$_4$ in DMSO-d_6.

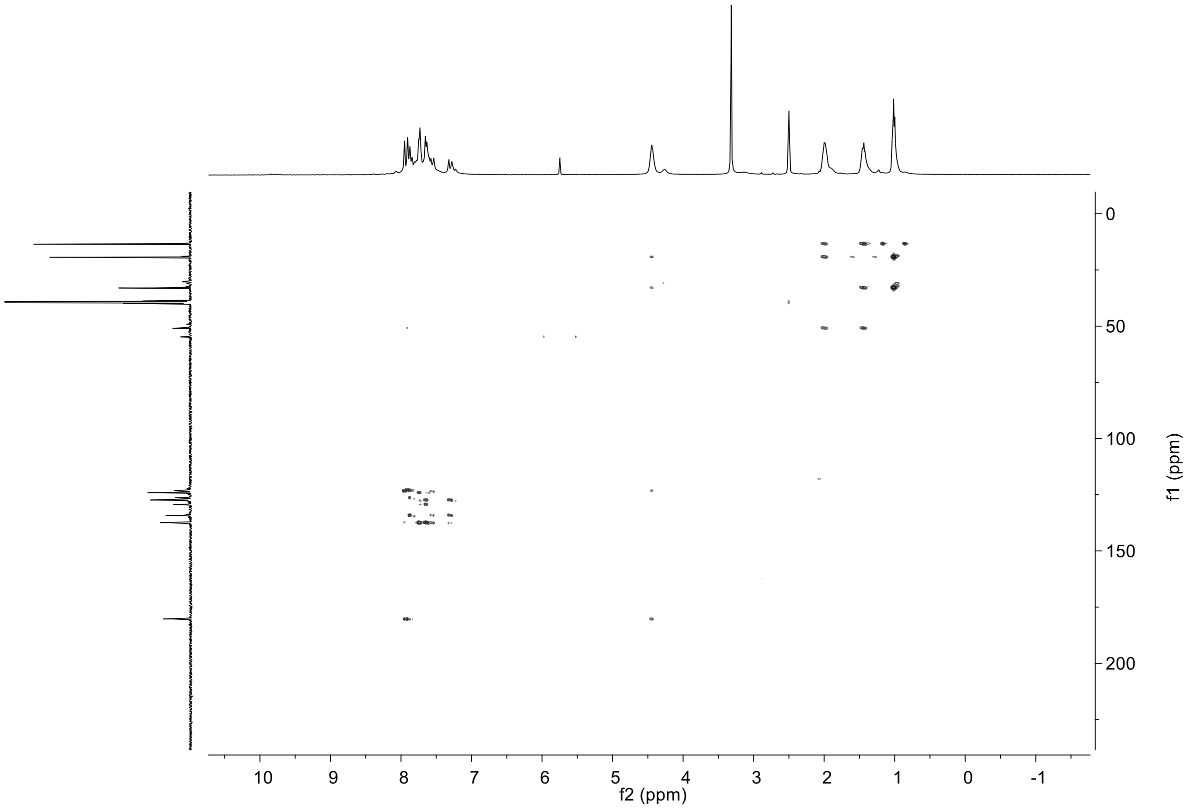

Figure S28. $^{1}H^{13}C$ HMBC spectrum (400 MHz, 101 MHz) of [**6**](BF_4)$_4$ in DMSO-d_6.

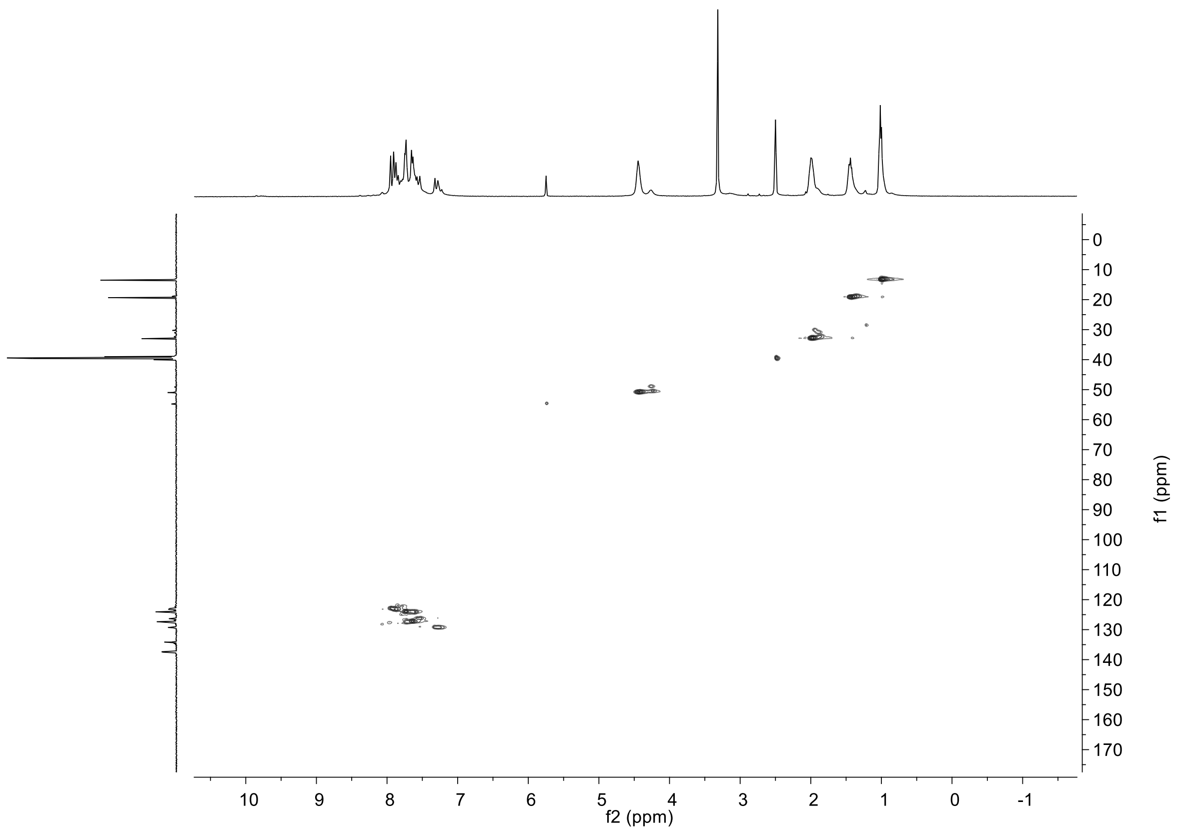

Figure S29. $^{1}H^{13}C$ HSQC spectrum (400 MHz, 101 MHz) of [**6**]$(BF_4)_4$ in DMSO-d_6.

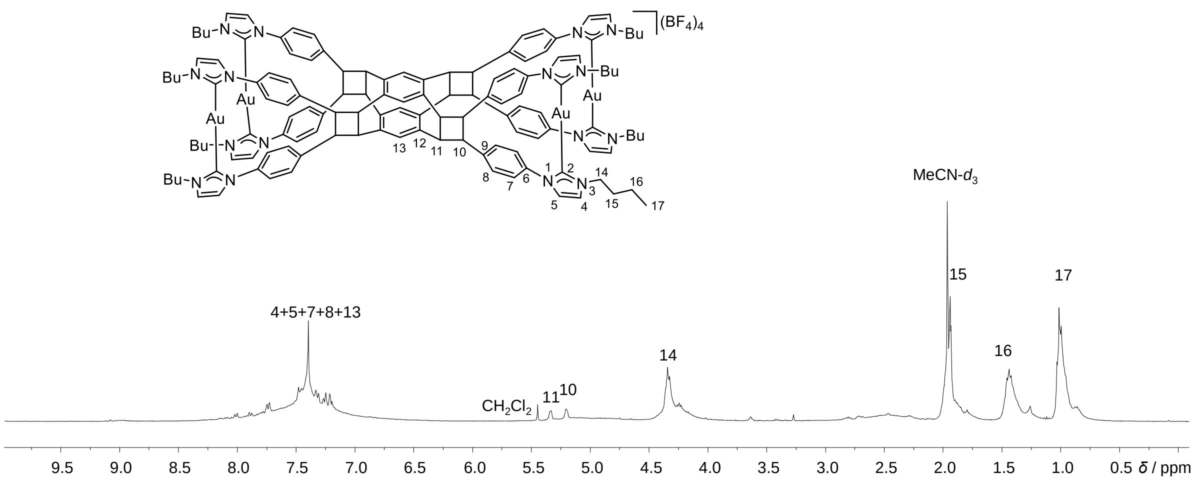

Figure S30. ^{1}H NMR (400.1 Hz) of [**7**]$(BF_4)_4$ in DMSO-d_6.

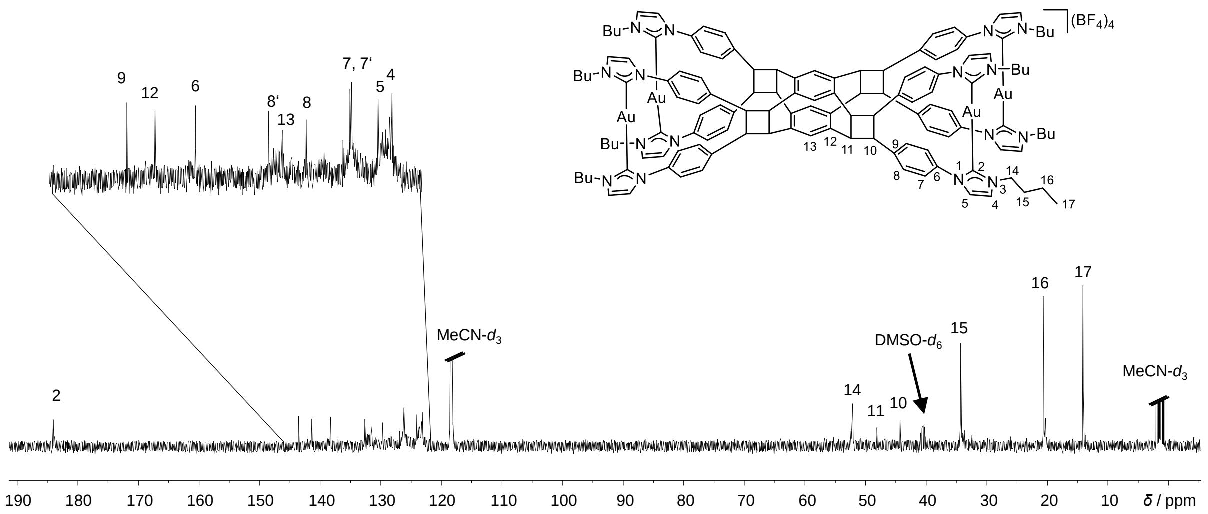

Figure S31. $^{13}C\{^1H\}$ NMR (101 Hz) of [**7**]$(BF_4)_4$ in DMSO-d_6.

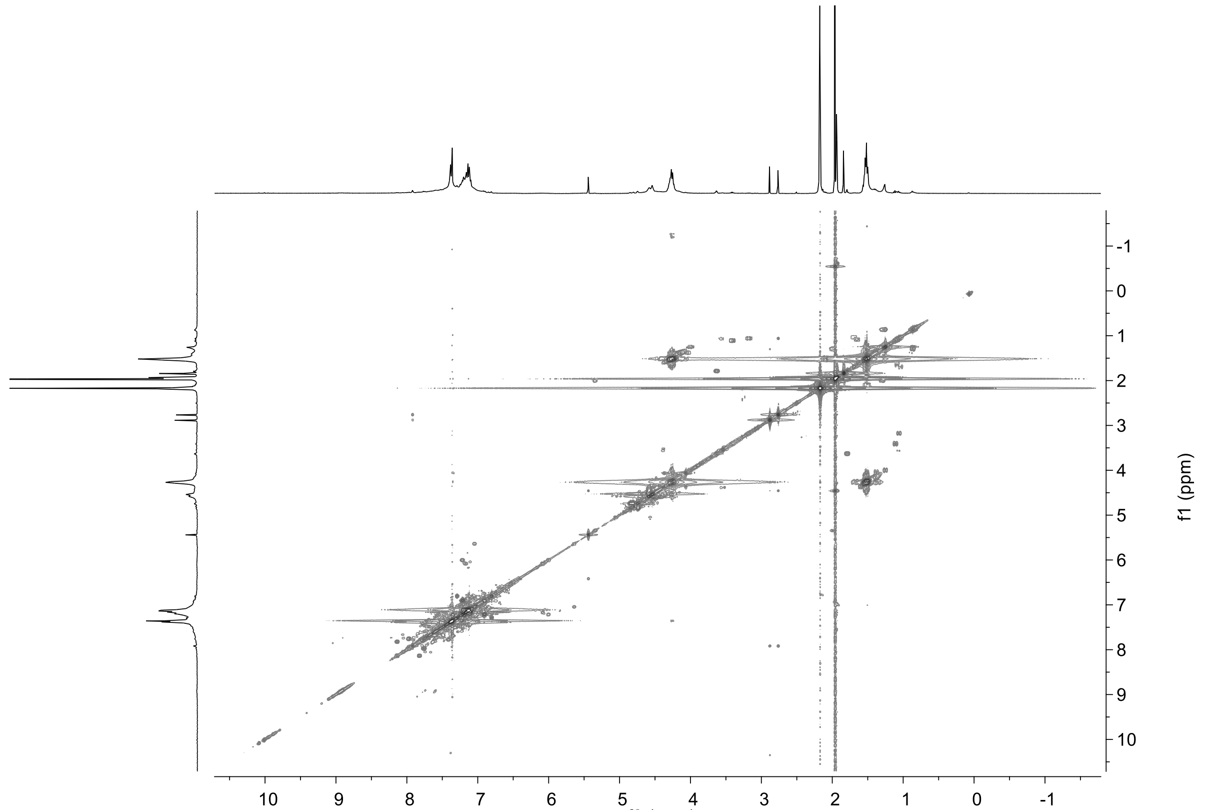

Figure S32. $^1H^1H$ COSY spectrum (400 MHz) of [**7**]$(BF_4)_4$ in DMSO-d_6.

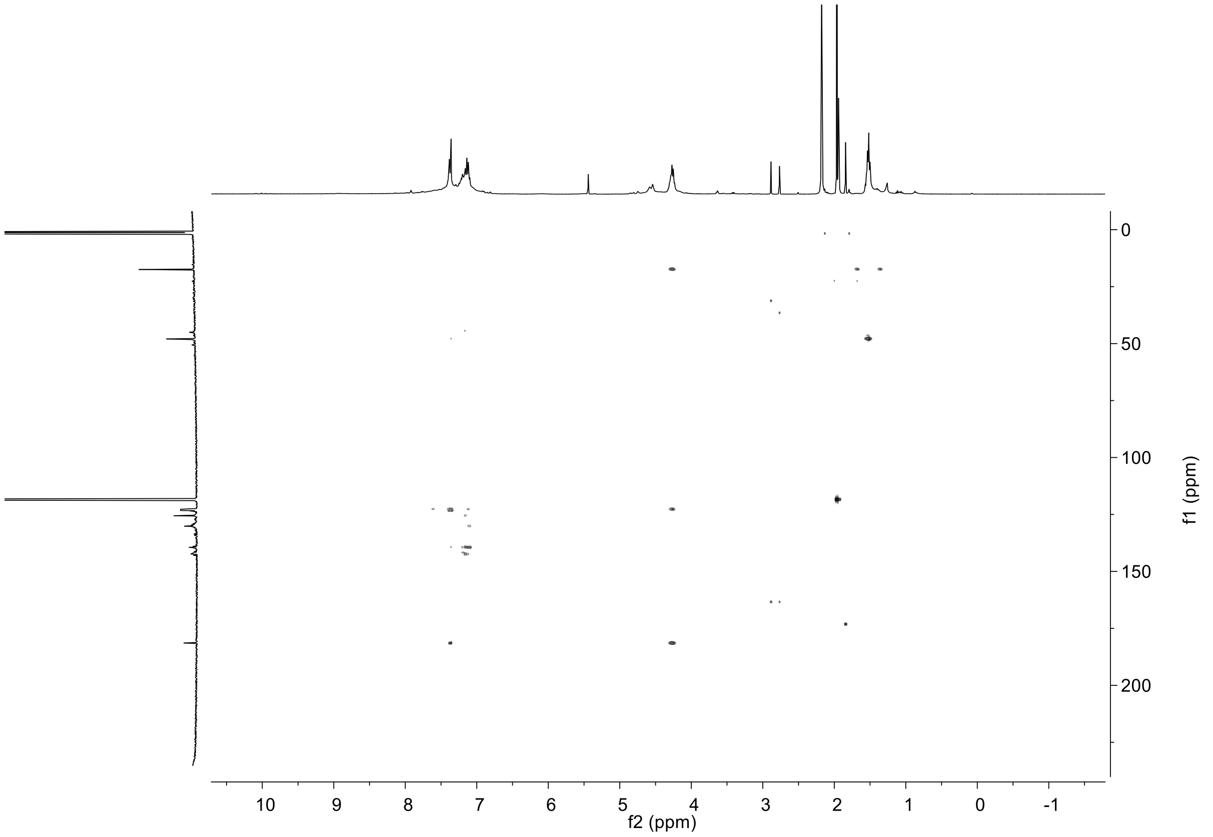

Figure S33. $^{1}H^{13}C$ HMBC spectrum (400 MHz, 101 MHz) of [**7**]$(BF_4)_4$ in DMSO-d_6.

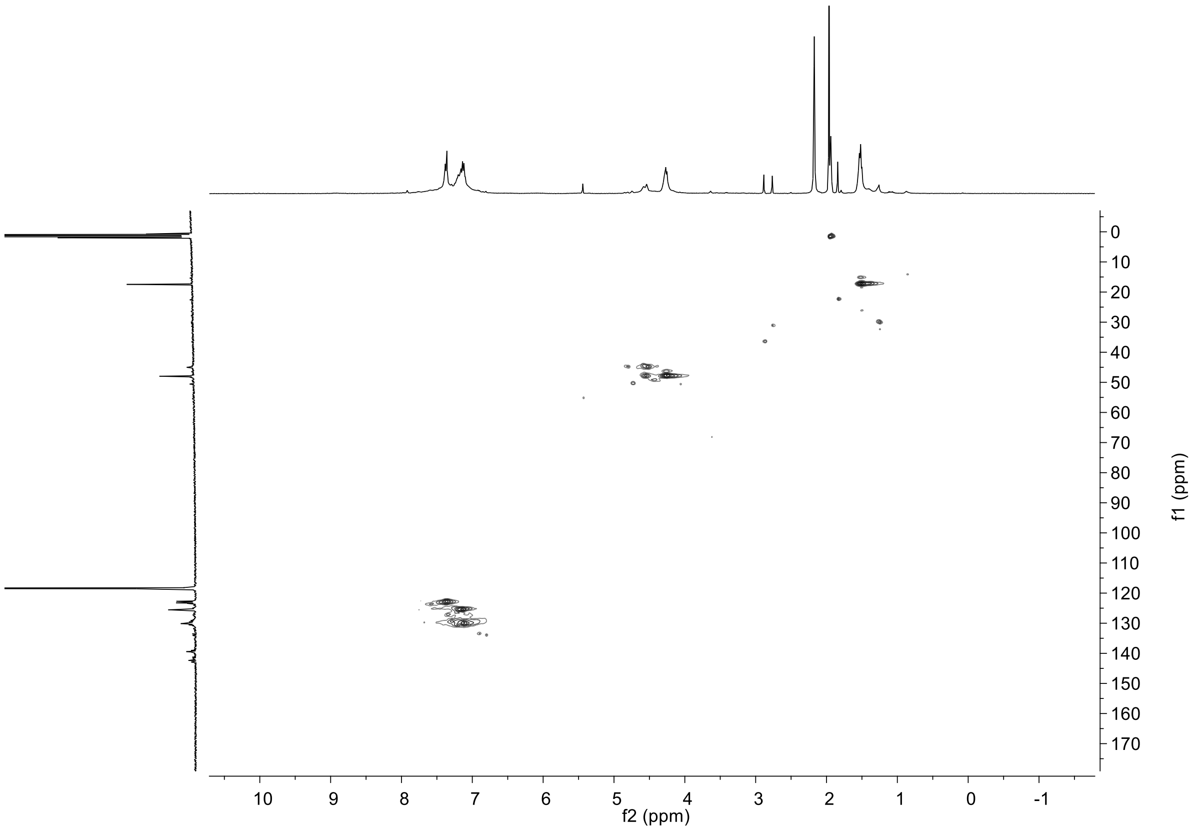

Figure S34. $^{1}H^{13}C$ HSQC spectrum (400 MHz, 101 MHz) of [**7**]$(BF_4)_4$ in DMSO-d_6.

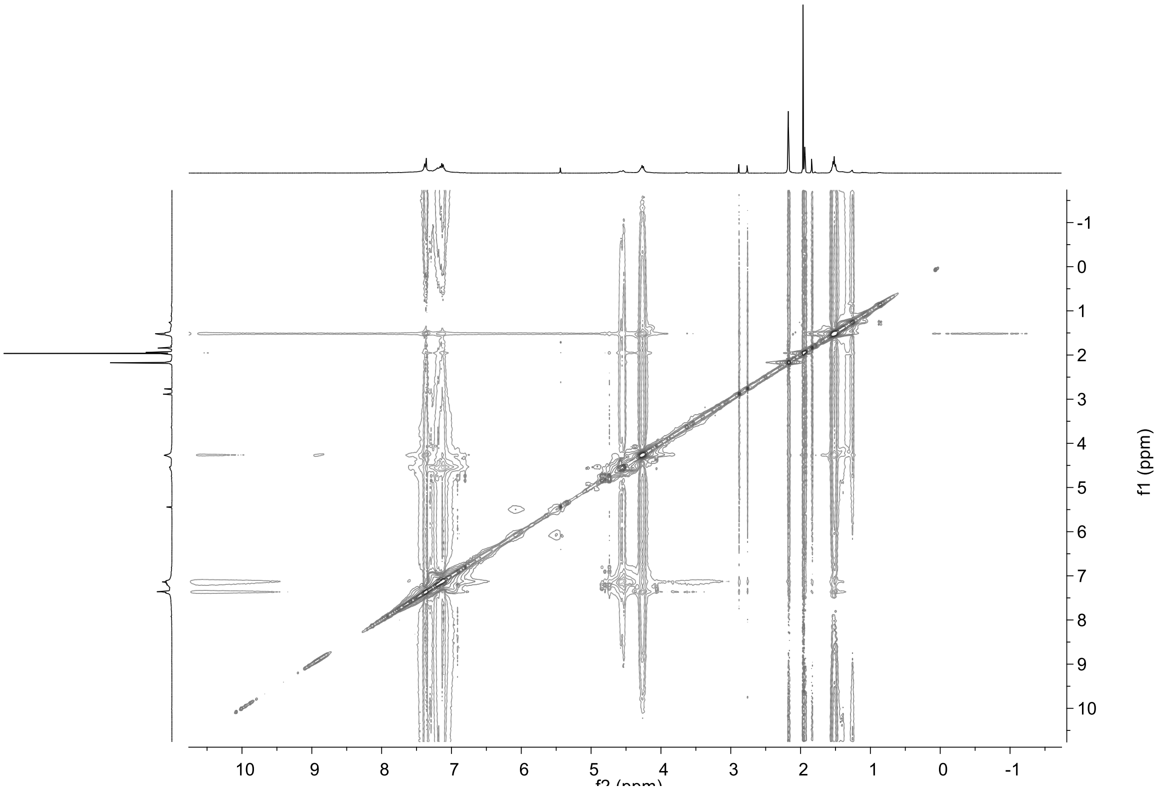

Figure S35. $^1H^1H$ ROESY spectrum (400 MHz) of [**7**](BF_4)$_4$ in DMSO-d_6.

4. X-ray crystallography

Colorless crystals of [**3**]$(BF_4)_2(BPh_4)_2$·4MeCN were obtained by stirring samples of [**3**]$(BF_4)_4$ (50 mg, 0.018 mmol) and $NaBPh_4$ (25.3 mg, 0.074 mmol) in acetonitrile followed by diffusion of diethyl ether into the acetonitrile solution. Single crystals of [**3**]$(BF_4)_2(BPh_4)_2$·4MeCN were mounted on a MicroMount® polymer tip (MiteGen) in random orientation. Diffraction data were collected on an Agilent SuperNova diffractometer equipped with an Atlas CCD detector using CuKα radiation (λ = 1.54184 Å). The raw data were corrected for absorption. A structure solution was found using Olex2[3] and SHELXT[4] and the structure was refined using the SHELXL program package.[5] Hydrogen atoms were introduced to the structure model on calculated positions and were refined as riding atoms. Non-hydrogen atoms were refined with anisotropic thermal parameters. An enhanced rigid distance restraint (SHELX SADI) and rigid bond restraint (SHELX RIGU) were applied to the positional parameters of the *n*-butyl groups. Disordered solvent molecules complicated the refinement. A solvent mask procedure was used to treat the significant disorder of the solvent molecules. The positional parameters of solvent molecules (diethyl ether) located inside the cylinder-like cavity of [**3**]$^{4+}$ could not be refined and the SQUEEZE procedure was employed to remove the effects of these solvent molecules.

Colorless crystals of [**4**]$(BPh_4)_2$ were obtained by stirring samples of [**4**]$(BF_4)_4$ (50 mg, 0.018 mmol) and $NaBPh_4$ (25.3 mg, 0.074 mmol) in acetonitrile, adding of THF and cooling of the resulting solution. Diffraction data for [**4**]$(BPh_4)_4$ were collected with a Bruker APEX-II CCD diffractometer equipped with a rotation anode using graphite-monochromated Mo-Kα radiation (λ = 0.71073 Å). Diffraction data were collected over the full sphere and were corrected for absorption. Structure solutions were found with SHELXT[4] using direct methods and were refined with SHELXL[5] against $|F^2|$ of all data using first isotropic and later anisotropic thermal parameters for all non-hydrogen atoms. Hydrogen atoms were added to the structure model on calculated positions.

Important crystal details and structure refinement parameters are summarized in Tables S1 and Table S2. Further crystallographic details can be found in the CIF files, which were deposited at the Cambridge Crystallographic Data Centre, Cambridge, UK. The reference number for compounds [**3**]$(BF_4)_2(BPh_4)_2$·4MeCN and [**4**]$(BPh_4)_4$ are 1972486 and 1972487, respectively.

Table S1. Crystal data and structure refinement details for [**3**]$(BF_4)_2(BPh_4)_2\cdot 4MeCN$

Empirical formula	$C_{188}H_{192}N_{20}Ag_4B_4F_8$
Formula weight	3358.32
Temperature, K	204(6)
Crystal system	monoclinic
Space group	*I*2/*a*
a, Å	14.0718(7)
b, Å	49.980(3)
c, Å	24.3788(10)
α, deg	90.0
β, deg	92.963(4)
γ, deg	90.0
Volume, Å^3	17123.0(15)
Z	4
ρ_{calcd}, g·cm^{-3}	1.303
μ, mm^{-1}	4.145
F(000)	6960
Crystal size, mm^3	0.45×0.06×0.03
Radiation	CuKα (λ = 1.54184 Å)
2Θ range, deg	6.4 to 133.2
Index ranges	–13≤h≤16, –54≤k≤59, –21≤l≤29
Intensities collected	29996
Independent intensities	15029 (R_{int} = 0.1131)
Observed intensities ($I \geq 2\sigma(I)$)	6573
Data/restraints/parameters	15029/53/1016
Goodness-of-fit on F^2	0.934
Final *R* ($I \geq 2\sigma(I)$)	*R* = 0.0869, *wR* = 0.2192
Final *R* (all data)	*R* = 0.1522, *wR* = 0.2769

Table S2. Crystal data and structure refinement details for [**4**]$(BPh_4)_4$

Empirical formula	$C_{228}H_{220}N_{16}Ag_4B_4$
Formula weight	3658.91
Temperature, K	296(2)
Crystal system	orthorhombic
Space group	*Fdd*2
a, Å	55.073(2)
b, Å	25.2451(9)
c, Å	34.4583(12)
α, deg	90.0
β, deg	90.0
γ, deg	90.0
Volume, Å^3	47909(3)
Z	8
ρ_{calcd}, g/cm^3	1.015
μ, mm^{-1}	0.370
F(000)	15264
Crystal size, mm^3	0.35×0.35×0.09
Radiation	MoKα (λ = 0.71073)
2Θ range, deg	2.1 to 51.6
Index ranges	$-67 \leq h \leq 67$, $-30 \leq k \leq 30$, $-42 \leq l \leq 42$
Intensities collected	145539
Independent intensities	22920 (R_{int} = 0.0979)
Observed intensities ($I \geq 2\sigma(I)$)	21868
Data/restraints/parameters	22920/1751/961
Goodness-of-fit on F^2	1.192
Final *R* ($I \geq 2\sigma(I)$)	*R* = 0.1245, *wR* = 0.3299
Final *R* (all data)	*R* = 0.1293, *wR* = 0.3333

5. Titration experiments

The anion recognition capability of salts H_4-**2**$(BF_4)_4$ and H_8-**5**$(BF_4)_8$ (hosts) was studied by ^1H NMR titration experiments, by addition of increasing amounts of the tetrabutylammonium salts of chloride, bromide, nitrate, benzoate, ATP^- and ibuprofen$^-$. The experiments were carried out in DMSO-d_6 at constant concentration of the host (2 mM except for NBu_4ATP 0.5 mM). Two solutions were prepared. Solution A (only containing the hosts at 2 mM or 0.5 mM) and solution B (containing hosts at 2 mM or 0.5 mM and guests at 53.6 mM or 13.4 mM). The addition of increasing amounts of solution B to solution A produced a shift of the resonances for the aromatic protons of the hosts. Generally, a non-cooperative model with parameter restrictions 1:2 (H:G) was used for the system H_8-**5**$(BF_4)_8$ with the tetrabutyl ammonium salts. For the system H_4-**2**$(BF_4)_4$ with the tetrabutyl ammonium salts two methods were used, a non-cooperative 1:2 (H:G) binding model and a model without parameter restrictions.

Table S3. Association constants for the formation of host-guest complexes between salts H_4-**2**(BF_4) and H_8-**5**$(BF_4)_8$ and some selected anions, in DMSO-d_6 at 25ºC.[a]

Entry	Host	Guest	K_{11} (M^{-1})	K_{12} (M^{-1})
1	H_4-**2**$(BF_4)_4$	ATP^-	[a]$1.60(2) \times 10^3$	4.0×10^2
			[b]$1.7(1) \times 10^3$	[b]$2.5(2) \times 10^2$
2	H_4-**2**$(BF_4)_4$	benzoate$^-$	[a]$1.97(4) \times 10^2$	49
			[b]290(8)	[b]34(2)
3	H_4-**2**$(BF_4)_4$	Br^-	[a]45(1)	10
			[b]40(1)	[b]N/A
4	H_4-**2**$(BF_4)_4$	Cl^-	[a]70(1)	18
			[b]60(2)	[b]N/A
5	H_4-**2**$(BF_4)_4$	ibuprofen$^-$	[a]133(2)	33
			[b]91	[b]N/A
6	H_4-**2**$(BF_4)_4$	NO_3^-	[a]31(1)	8
			[b]29(1)	[b]N/A
7	H_8-**5**$(BF_4)_8$	ATP^-	$8.4(4) \times 10^3$	2.1×10^3
8	H_8-**5**$(BF_4)_8$	benzoate$^-$	$7.4(1) \times 10^2$	1.8×10^2
9	H_8-**5**$(BF_4)_8$	Br^-	86(3)	21
10	H_8-**5**$(BF_4)_8$	Cl^-	$4.53(5) \times 10^2$	1.25×10^2
11	H_8-**5**$(BF_4)_8$	ibuprofen$^-$	$7.6(1) \times 10^2$	1.9×10^2
12	H_8-**5**$(BF_4)_8$	NO_3^-	390(9)	98

[a]Association constants were calculated by global nonlinear regression analysis[6] and assuming a non-cooperative 1:2 (H:G) binding model.[7] All anions added as tetrabutyl ammonium salts. [b]Association constants calculated without parameter restrictions. N/A: the value resulting from the fitting was too small or did not have any physical meaning.

5.1. ^{1}H NMR titration experiments with H_8-5$(BF_4)_8$

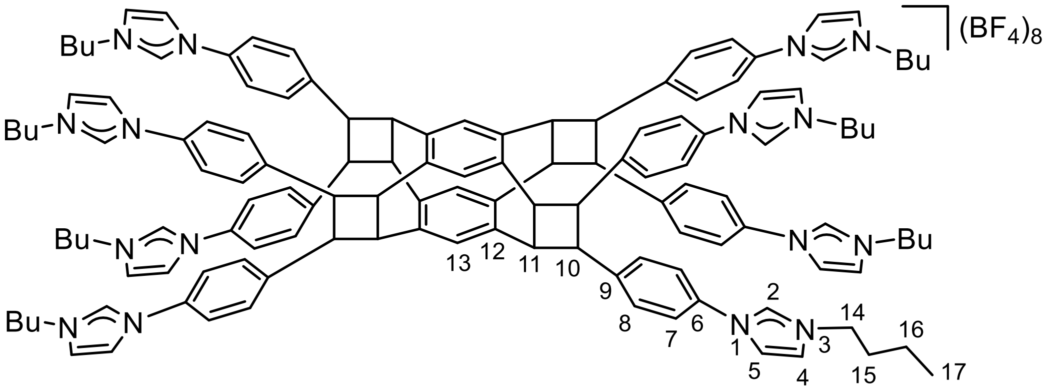

H_8-**5**$(BF_4)_8$

a. Br^- titration

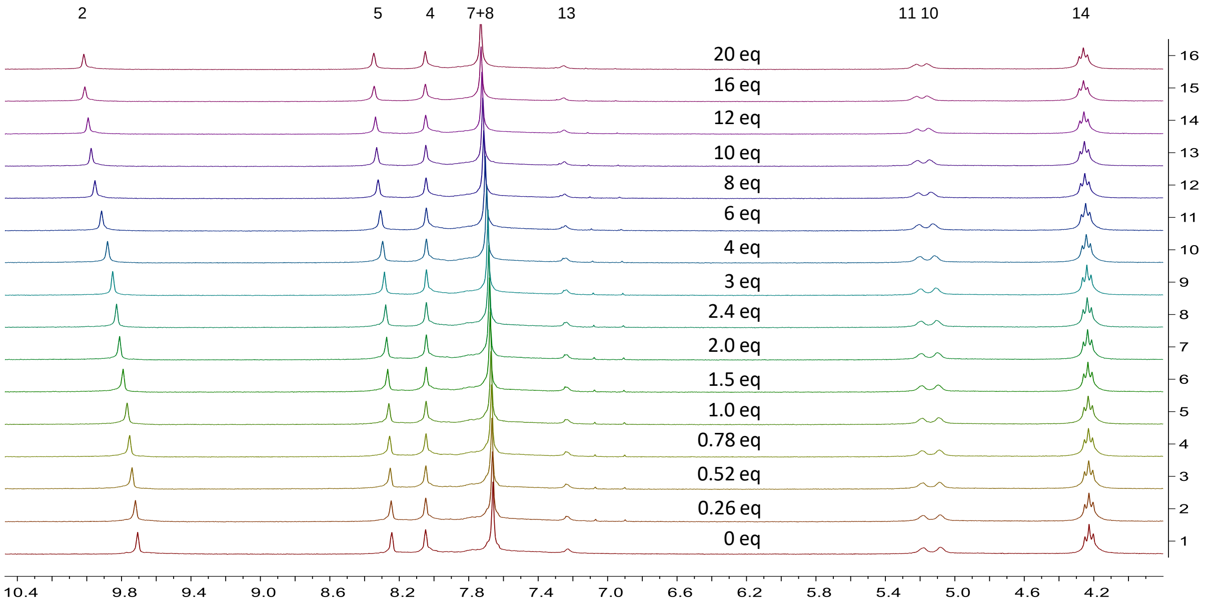

Figure S36. Partial ^{1}H-NMR (300 MHz) changes observed for the host H_8-**5**$(BF_4)_8$ in DMSO-d_6 during the addition of Br^-.

Table S4. Data values from the titration study of H_8-**5**$(BF_4)_8$ with Br^-

H_8-**5**$(BF_4)_8$ M / mol	NBu_4Br M / mol	Eq NBu_4Br	δ_{H2} [ppm]	δ_{H5} [ppm]	$\Delta\delta_{H2}$ [ppm]	$\Delta\delta_{H5}$ [ppm]
$2.00\cdot10^{-3}$	0.00	0.00	9.707	8.243	0.000	0
$2.00\cdot10^{-3}$	$5.30\cdot10^{-4}$	0.26	9.720	8.247	0.013	0.004
$2.00\cdot10^{-3}$	$1.05\cdot10^{-3}$	0.52	9.739	8.252	0.032	0.009
$2.00\cdot10^{-3}$	$1.56\cdot10^{-3}$	0.78	9.753	8.255	0.046	0.012
$2.00\cdot10^{-3}$	$2.06\cdot10^{-3}$	1.0	9.767	8.260	0.060	0.017
$2.00\cdot10^{-3}$	$3.03\cdot10^{-3}$	1.5	9.790	8.266	0.083	0.023
$2.00\cdot10^{-3}$	$3.97\cdot10^{-3}$	2.0	9.811	8.273	0.104	0.03
$2.00\cdot10^{-3}$	$4.87\cdot10^{-3}$	2.4	9.828	8.278	0.121	0.035
$2.00\cdot10^{-3}$	$6.16\cdot10^{-3}$	3.1	9.851	8.285	0.144	0.042
$2.00\cdot10^{-3}$	$8.17\cdot10^{-3}$	4.1	9.880	8.296	0.173	0.053
$2.00\cdot10^{-3}$	$1.14\cdot10^{-2}$	5.7	9.915	8.304	0.208	0.061
$2.00\cdot10^{-3}$	$1.61\cdot10^{-2}$	8.0	9.953	8.322	0.246	0.079
$2.00\cdot10^{-3}$	$2.01\cdot10^{-2}$	10.0	9.975	8.330	0.268	0.087
$2.00\cdot10^{-3}$	$2.41\cdot10^{-2}$	12.0	9.992	8.337	0.285	0.094
$2.00\cdot10^{-3}$	$3.21\cdot10^{-2}$	16.0	10.011	8.344	0.304	0.101
$2.00\cdot10^{-3}$	$4.02\cdot10^{-2}$	20.0	10.017	8.347	0.31	0.104

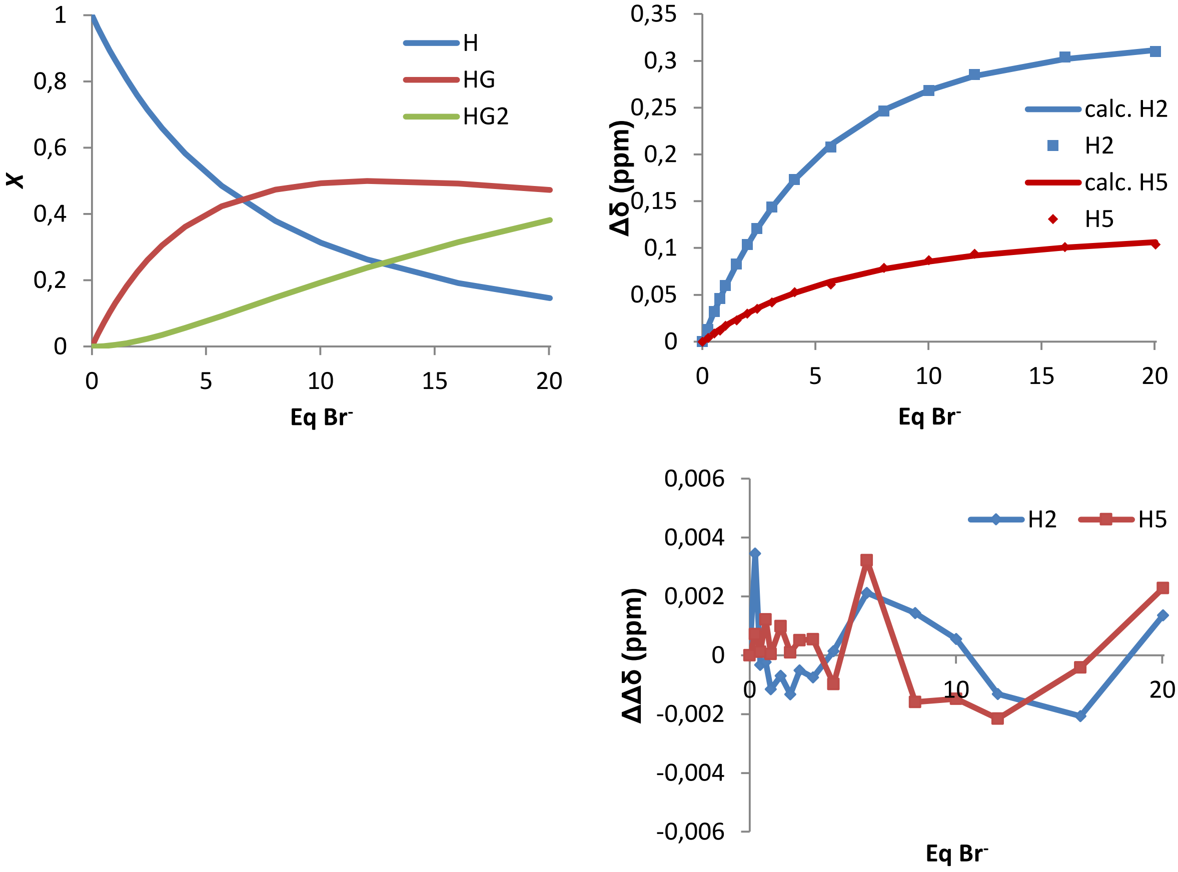

Figure S37. Non-linear least-squares fitting calculated nonlinear regression analysis and assuming a non-cooperative 1:2 (H:G) binding model of the chemical shift changes of H2 and H5 during titration experiments of H_8-**5**$(BF_4)_4$ with Br^-. The graph on the left represents the speciation profiles.

b. benzoate titration

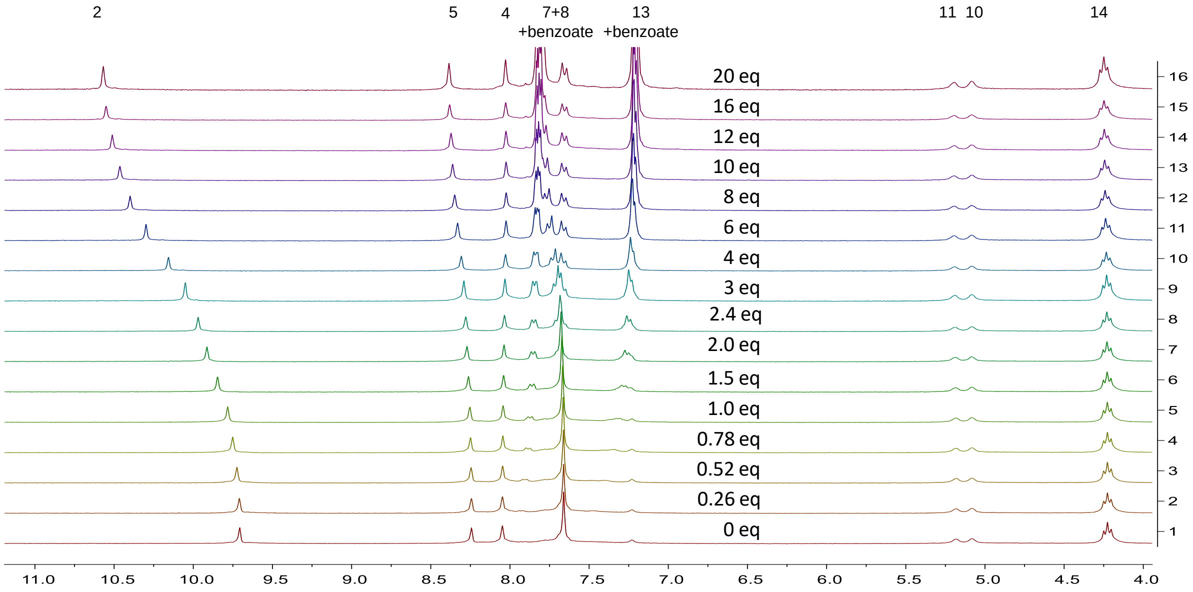

Figure S38. Partial ^{1}H-NMR (300 MHz) changes observed for the host H_8-**5**$(BF_4)_8$ in DMSO-d_6 during the addition of benzoate.

Table S5. Data values from the titration study of H_8-**5**$(BF_4)_8$ with benzoate

H_8-**5**$(BF_4)_8$ M / mol	NBu_4PhCOO M / mol	Eq NBu_4PhCOO	δ_{H2} [ppm]	δ_{H5} [ppm]	δ_{H4} [ppm]	$\Delta\delta_{H2}$ [ppm]	$\Delta\delta_{H5}$ [ppm]	$\Delta\delta_{H4}$ [ppm]
$2.00\cdot10^{-3}$	0.00	0.00	9.706	8.243	8.047	0.000	0.000	0.000
$2.00\cdot10^{-3}$	$5.30\cdot10^{-4}$	0.26	9.709	8.243	8.047	0.003	0.000	0.000
$2.00\cdot10^{-3}$	$1.05\cdot10^{-3}$	0.52	9.724	8.245	8.046	0.018	0.002	–0.001
$2.00\cdot10^{-3}$	$1.56\cdot10^{-3}$	0.78	9.751	8.249	8.045	0.045	0.006	–0.002
$2.00\cdot10^{-3}$	$2.06\cdot10^{-3}$	1.0	9.782	8.253	8.043	0.076	0.010	–0.004
$2.00\cdot10^{-3}$	$3.03\cdot10^{-3}$	1.5	9.847	8.262	8.040	0.141	0.019	–0.007
$2.00\cdot10^{-3}$	$3.97\cdot10^{-3}$	2.0	9.914	8.271	8.037	0.208	0.028	–0.010
$2.00\cdot10^{-3}$	$4.87\cdot10^{-3}$	2.4	9.969	8.279	8.035	0.263	0.036	–0.012
$2.00\cdot10^{-3}$	$6.17\cdot10^{-3}$	3.1	10.050	8.291	8.031	0.344	0.048	–0.016
$2.00\cdot10^{-3}$	$8.18\cdot10^{-3}$	4.1	10.157	8.307	8.028	0.451	0.064	–0.019
$2.00\cdot10^{-3}$	$1.20\cdot10^{-2}$	6.0	10.299	8.330	8.025	0.593	0.087	–0.022
$2.00\cdot10^{-3}$	$1.61\cdot10^{-2}$	8.0	10.399	8.349	8.024	0.693	0.106	–0.023
$2.00\cdot10^{-3}$	$2.01\cdot10^{-2}$	10.0	10.464	8.362	8.024	0.758	0.119	–0.023
$2.00\cdot10^{-3}$	$2.41\cdot10^{-2}$	12.0	10.512	8.372	8.025	0.806	0.129	–0.022
$2.00\cdot10^{-3}$	$3.21\cdot10^{-2}$	16.0	10.550	8.381	8.027	0.844	0.138	–0.020
$2.00\cdot10^{-3}$	$4.02\cdot10^{-2}$	20.0	10.568	8.385	8.028	0.862	0.142	–0.019

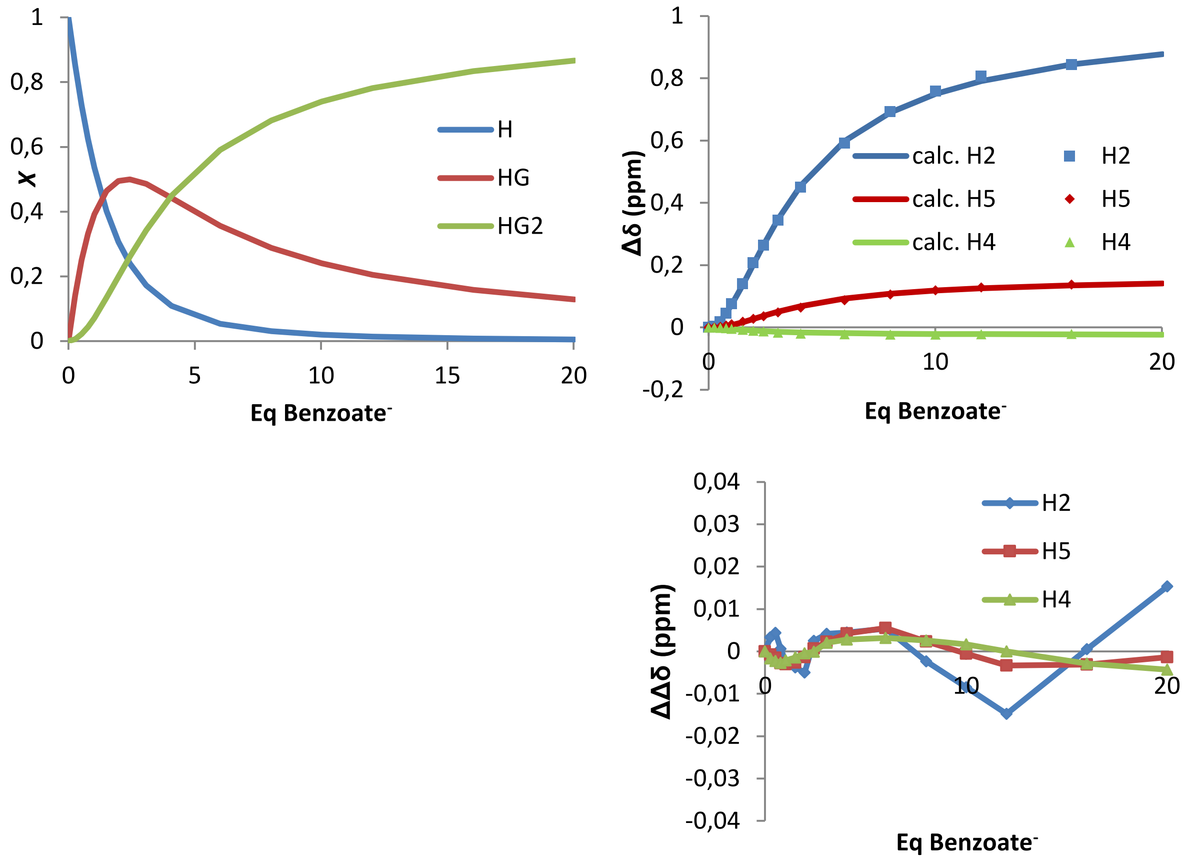

Figure S39. Non-linear least-squares fitting calculated nonlinear regression analysis and assuming a non-cooperative 1:2 (H:G) binding model of the chemical shift changes of H2, H4 and H5 during titration experiments of H_8-**5**$(BF_4)_4$ with benzoate. The graph on the left represents the speciation profiles.

c. Cl^- titration

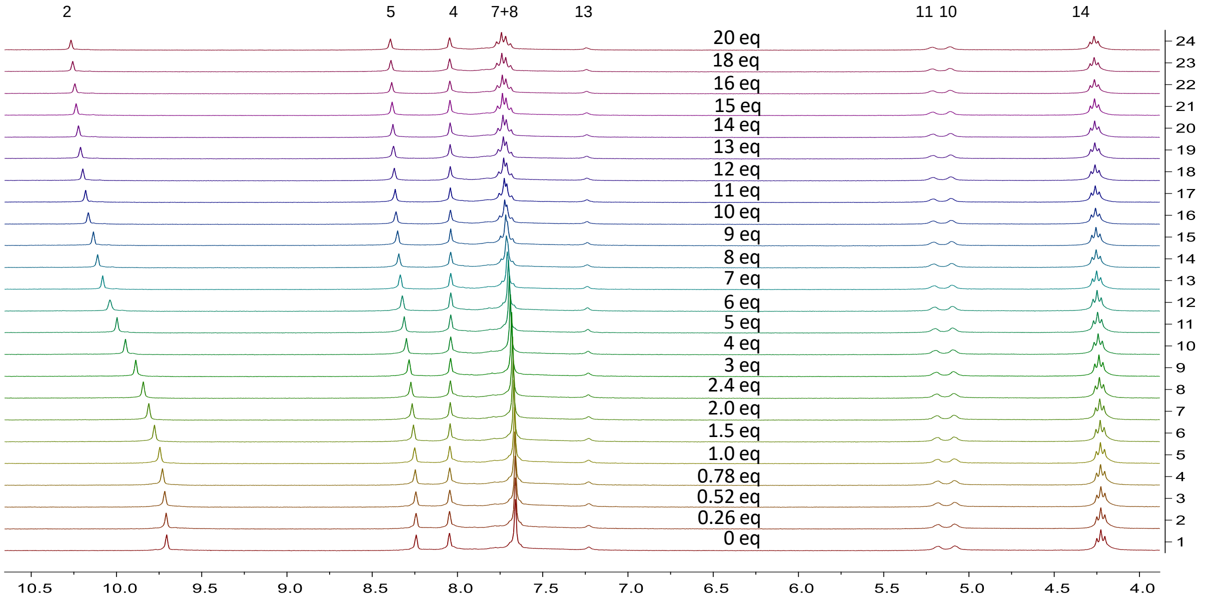

Figure S40. Partial ^{1}H-NMR (300 MHz) changes observed for the host H_8-**5**$(BF_4)_8$ in DMSO-d_6 during the addition of Cl^-.

Table S6. Data values from the titration study of H_8-**5**$(BF_4)_8$ with Cl^-

H_8-**5**$(BF_4)_8$ M / mol	NBu_4Cl M / mol	Eq NBu_4Cl	δ_{H2} [ppm]	δ_{H5} [ppm]	δ_{H4} [ppm]	$\Delta\delta_{H2}$ [ppm]	$\Delta\delta_{H5}$ [ppm]	$\Delta\delta_{H4}$ [ppm]
$2.00\cdot10^{-3}$	0.00	0.00	9.705	8.242	8.047	0	0	0
$2.00\cdot10^{-3}$	$5.30\cdot10^{-4}$	0.26	9.708	8.243	8.046	0.003	0.001	–0.001
$2.00\cdot10^{-3}$	$1.05\cdot10^{3}$	0.52	9.717	8.244	8.045	0.012	0.002	–0.002
$2.00\cdot10^{-3}$	$1.56\cdot10^{-3}$	0.78	9.73	8.247	8.045	0.025	0.005	–0.002
$2.00\cdot10^{-3}$	$2.06\cdot10^{-3}$	1.0	9.745	8.251	8.044	0.04	0.009	–0.003
$2.00\cdot10^{-3}$	$3.03\cdot10^{-3}$	1.5	9.777	8.258	8.043	0.072	0.016	–0.004
$2.00\cdot10^{-3}$	$3.97\cdot10^{-3}$	2.0	9.81	8.265	8.042	0.105	0.023	–0.005
$2.00\cdot10^{-3}$	$4.87\cdot10^{-3}$	2.4	9.843	8.273	8.041	0.138	0.031	–0.006
$2.00\cdot10^{-3}$	$6.16\cdot10^{-3}$	3.1	9.887	8.284	8.04	0.182	0.042	–0.007
$2.00\cdot10^{-3}$	$8.17\cdot10^{-3}$	4.1	9.947	8.299	8.04	0.242	0.057	–0.007
$2.00\cdot10^{-3}$	$1.00\cdot10^{-2}$	5.0	9.996	8.312	8.039	0.291	0.07	–0.008
$2.00\cdot10^{-3}$	$1.21\cdot10^{-2}$	6.0	10.038	8.323	8.039	0.333	0.081	–0.008
$2.00\cdot10^{-3}$	$14.2\cdot10^{-2}$	7.1	10.081	8.335	8.039	0.376	0.093	–0.008
$2.00\cdot10^{-3}$	$1.61\cdot10^{-2}$	8.1	10.11	8.344	8.04	0.405	0.102	–0.007
$2.00\cdot10^{-3}$	$1.81\cdot10^{-2}$	9.0	10.135	8.351	8.039	0.43	0.109	–0.008
$2.00\cdot10^{-3}$	$2.05\cdot10^{-2}$	10.2	10.165	8.36	8.041	0.46	0.118	–0.006
$2.00\cdot10^{-3}$	$2.22\cdot10^{-2}$	11.1	10.18	8.365	8.042	0.475	0.123	–0.005
$2.00\cdot10^{-3}$	$2.42\cdot10^{-2}$	12.1	10.197	8.37	8.042	0.492	0.128	–0.005
$2.00\cdot10^{-3}$	$2.61\cdot10^{-2}$	13.0	10.211	8.375	8.043	0.506	0.133	–0.004
$2.00\cdot10^{-3}$	$2.82\cdot10^{-2}$	14.0	10.223	8.379	8.043	0.518	0.137	–0.004
$2.00\cdot10^{-3}$	$3.03\cdot10^{-2}$	15.1	10.236	8.383	8.044	0.531	0.141	–0.003
$2.00\cdot10^{-3}$	$3.22\cdot10^{-2}$	16.1	10.244	8.385	8.045	0.539	0.143	–0.002
$2.00\cdot10^{-3}$	$3.61\cdot10^{-2}$	18.0	10.256	8.39	8.046	0.551	0.148	–0.001
$2.00\cdot10^{-3}$	$4.03\cdot10^{-2}$	20.1	10.267	8.393	8.046	0.562	0.151	–0.001

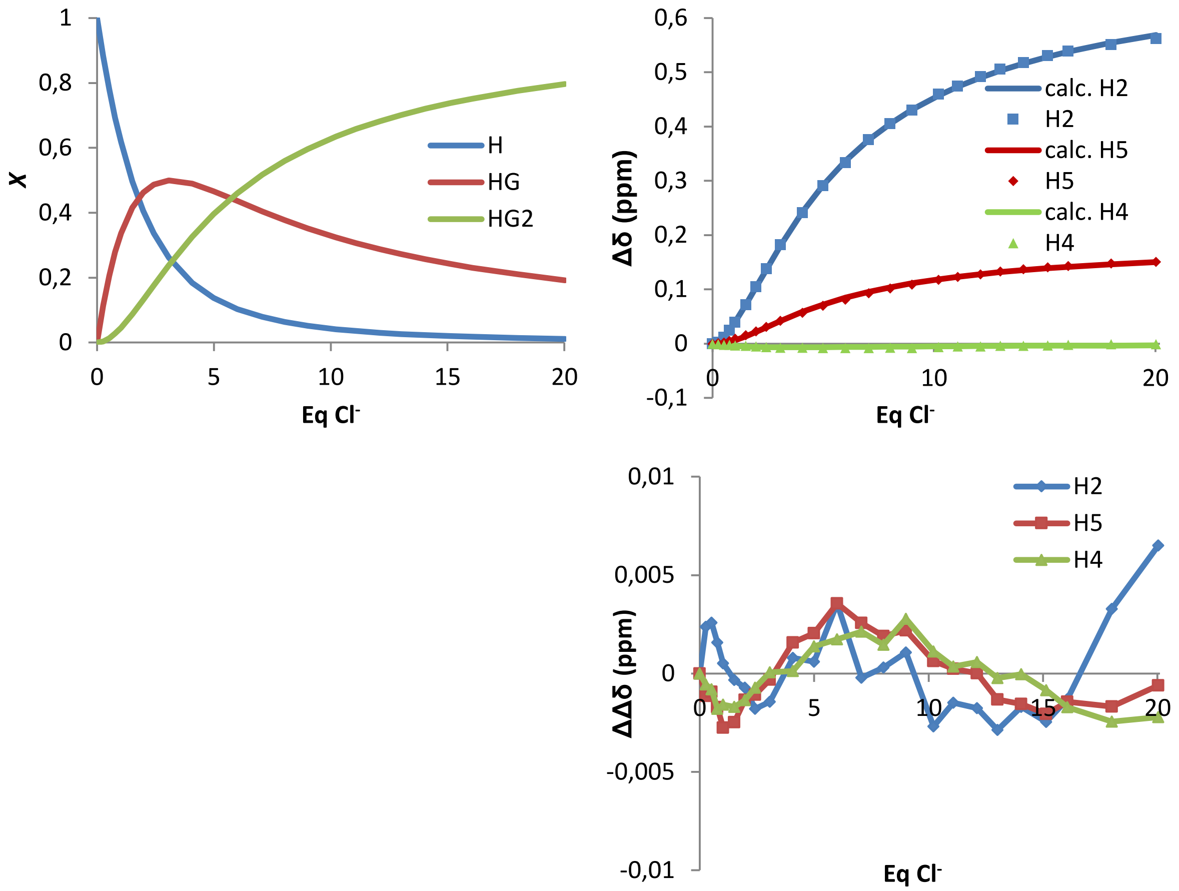

Figure S41. Non-linear least-squares fitting calculated nonlinear regression analysis and assuming a non-cooperative 1:2 (H:G) binding model of the chemical shift changes of H2, H4 and H5 during titration experiments of H_8-**5**$(BF_4)_4$ with Cl^-. The graph on the left represents the speciation profiles.

d. NO3- titration

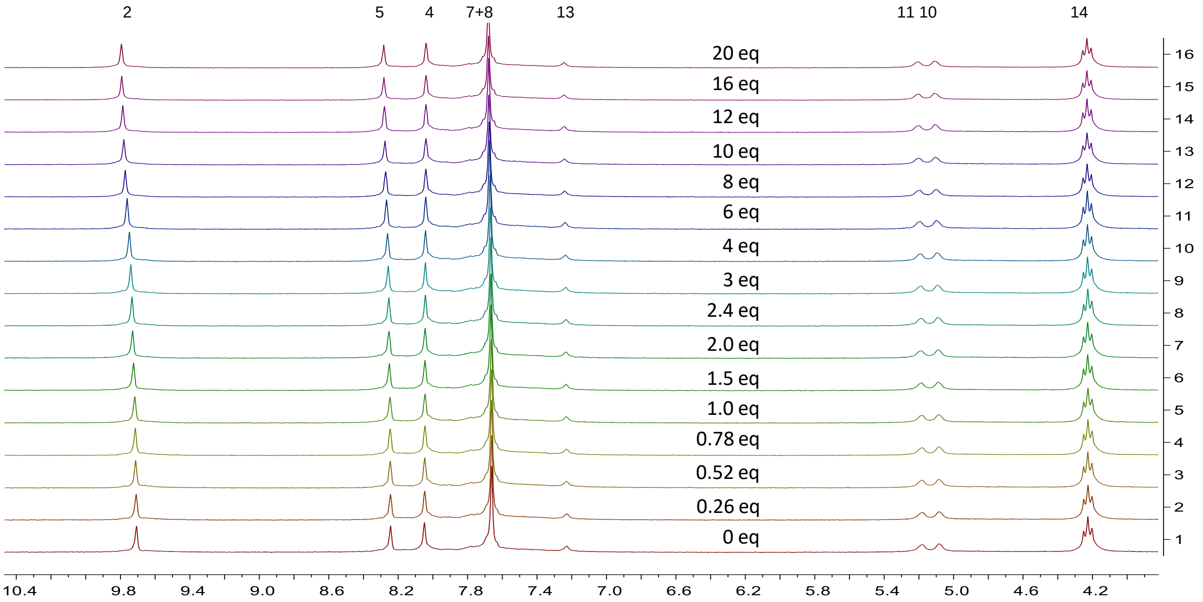

Figure S42. Partial ^{1}H-NMR (300 MHz) changes observed for the host H_8-**5**$(BF_4)_8$ in DMSO-*d*$_6$ during the addition of NO_3^-.

Table S7. Data values from the titration study of H_8-**5**$(BF_4)_8$ with NO_3^-

H_8-**5**$(BF_4)_8$ M / mol	NBu_4NO_3 M / mol	Eq NBu_4NO_3	δ_{H2} [ppm]	δ_{H5} [ppm]	$\Delta\delta_{H2}$ [ppm]	$\Delta\delta_{H5}$ [ppm]
$2.00 \cdot 10^{-3}$	0	0	9.707	0	8.243	0
$2.00 \cdot 10^{-3}$	$5.31 \cdot 10^{-4}$	0.26	9.709	0.002	8.244	0.002
$2.00 \cdot 10^{-3}$	$1.05 \cdot 10^{-3}$	0.52	9.713	0.006	8.245	0.006
$2.00 \cdot 10^{-3}$	$1.56 \cdot 10^{-3}$	0.78	9.714	0.007	8.246	0.007
$2.00 \cdot 10^{-3}$	$2.06 \cdot 10^{-3}$	1.0	9.717	0.01	8.247	0.01
$2.00 \cdot 10^{-3}$	$3.04 \cdot 10^{-3}$	1.5	9.724	0.017	8.25	0.017
$2.00 \cdot 10^{-3}$	$3.97 \cdot 10^{-3}$	2.0	9.729	0.022	8.252	0.022
$2.00 \cdot 10^{-3}$	$4.88 \cdot 10^{-3}$	2.4	9.733	0.026	8.254	0.026
$2.00 \cdot 10^{-3}$	$6.17 \cdot 10^{-3}$	3.1	9.74	0.033	8.257	0.033
$2.00 \cdot 10^{-3}$	$8.18 \cdot 10^{-3}$	4.1	9.748	0.041	8.261	0.041
$2.00 \cdot 10^{-3}$	$1.20 \cdot 10^{-2}$	6.0	9.761	0.054	8.267	0.054
$2.00 \cdot 10^{-3}$	$1.61 \cdot 10^{-2}$	8.0	9.772	0.065	8.272	0.065
$2.00 \cdot 10^{-3}$	$2.01 \cdot 10^{-2}$	10.0	9.78	0.073	8.276	0.073
$2.00 \cdot 10^{-3}$	$2.42 \cdot 10^{-2}$	12.1	9.786	0.079	8.279	0.079
$2.00 \cdot 10^{-3}$	$3.22 \cdot 10^{-2}$	16.1	9.793	0.086	8.282	0.086
$2.00 \cdot 10^{-3}$	$4.02 \cdot 10^{-2}$	20.1	9.794	0.087	8.283	0.087

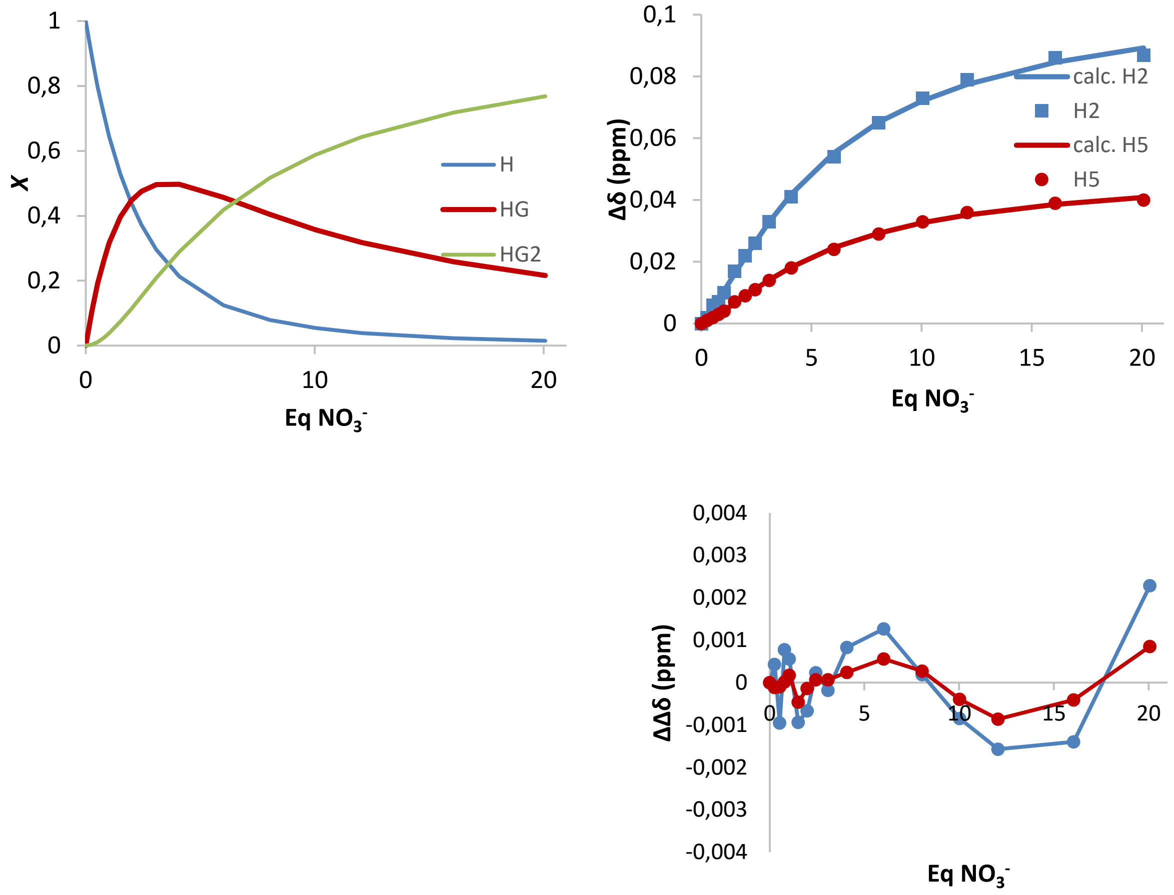

Figure S43. Non-linear least-squares fitting calculated nonlinear regression analysis and assuming a non-cooperative 1:2 (H:G) binding model of the chemical shift changes of H2 and H5 during titration experiments of H_8-**5**$(BF_4)_4$ with NO_3^-. The graph on the left represents the speciation profiles.

e. ibuprofen⁻ titration

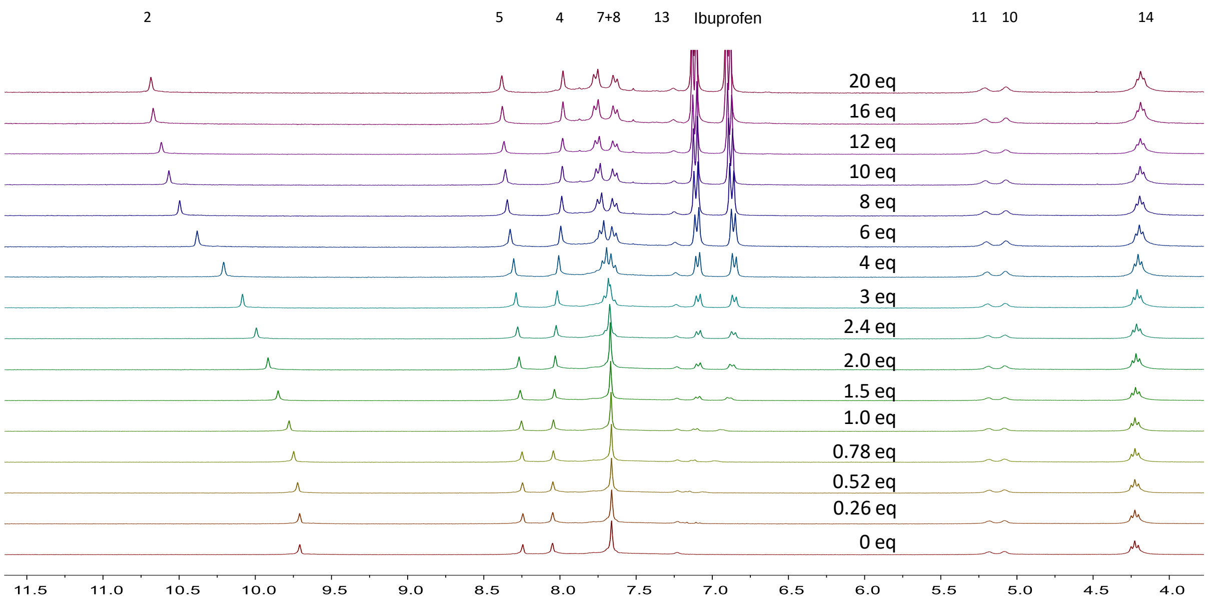

Figure S44. Partial ^{1}H-NMR (300 MHz) changes observed for the host H_8-**5**$(BF_4)_8$ in DMSO-d_6 during the addition of ibuprofen$^-$.

Table S8. Data values from the titration study of H_8-**5**$(BF_4)_8$ with ibuprofen$^-$

H_8-**5**$(BF_4)_8$ M / mol	(NBu_4)(ibuprofen) M / mol	Eq (NBu_4)(ibuprofen)	δ_{H2} [ppm]	δ_{H5} [ppm]	δ_{H4} [ppm]	$\Delta\delta_{H2}$ [ppm]	$\Delta\delta_{H5}$ [ppm]	$\Delta\delta_{H4}$ [ppm]
$2.00\cdot10^{-3}$	0.00	0.00	9.708	8.244	8.049	0.000	0.000	–0.000
$2.00\cdot10^{-3}$	$5.33\cdot10^{-4}$	0.27	9.709	8.243	8.047	0.001	–0.001	–0.000
$2.00\cdot10^{-3}$	$1.05\cdot10^{-3}$	0.53	9.722	8.246	8.047	0.014	0.002	–0.000
$2.00\cdot10^{-3}$	$1.57\cdot10^{-3}$	0.78	9.748	8.248	8.044	0.040	0.004	–0.005
$2.00\cdot10^{-3}$	$2.07\cdot10^{-3}$	1.0	9.778	8.253	8.042	0.070	0.009	–0.007
$2.00\cdot10^{-3}$	$3.04\cdot10^{-3}$	1.5	9.850	8.261	8.036	0.142	0.017	–0.013
$2.00\cdot10^{-3}$	$3.99\cdot10^{-3}$	2.0	9.916	8.268	8.031	0.208	0.024	–0.018
$2.00\cdot10^{-3}$	$4.89\cdot10^{-3}$	2.4	9.993	8.277	8.025	0.285	0.033	–0.024
$2.00\cdot10^{-3}$	$6.19\cdot10^{-3}$	3.1	10.084	8.288	8.018	0.376	0.044	–0.031
$2.00\cdot10^{-3}$	$8.20\cdot10^{-3}$	4.1	10.208	8.304	8.008	0.500	0.060	–0.041
$2.00\cdot10^{-3}$	$1.21\cdot10^{-2}$	6.0	10.382	8.328	7.995	0.674	0.084	–0.054
$2.00\cdot10^{-3}$	$1.61\cdot10^{-2}$	8.1	10.496	8.346	7.988	0.788	0.102	–0.061
$2.00\cdot10^{-3}$	$2.01\cdot10^{-2}$	10.1	10.568	8.358	7.985	0.860	0.114	–0.064
$2.00\cdot10^{-3}$	$2.42\cdot10^{-2}$	12.1	10.617	8.367	7.983	0.909	0.123	–0.066
$2.00\cdot10^{-3}$	$3.23\cdot10^{-2}$	16.1	10.671	8.379	7.980	0.963	0.135	–0.069
$2.00\cdot10^{-3}$	$4.03\cdot10^{-2}$	20.1	10.686	8.382	7.980	0.978	0.138	–0.069

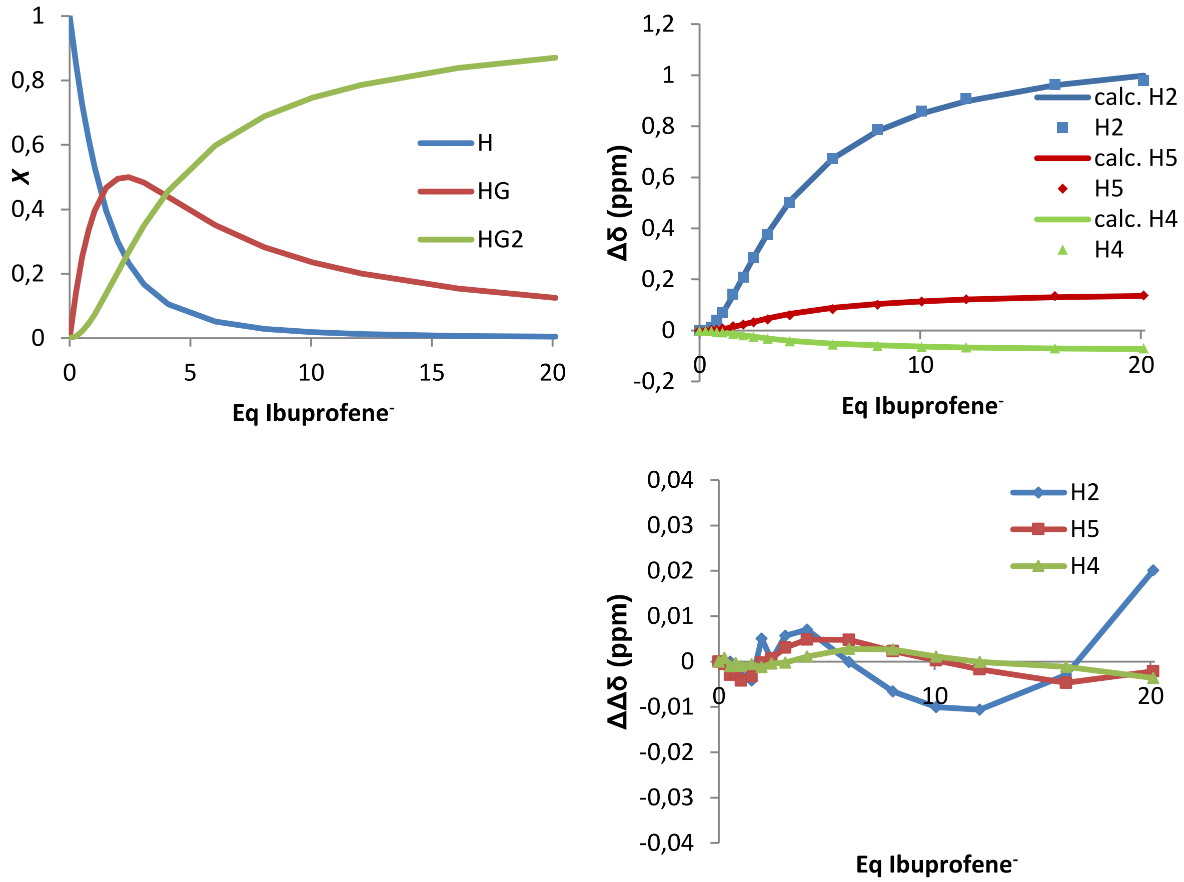

Figure S45. Non-linear least-squares fitting calculated nonlinear regression analysis and assuming a non-cooperative 1:2 (H:G) binding model of the chemical shift changes of H2, H4 and H5 during titration experiments of H_8-**5**$(BF_4)_4$ with ibuprofen⁻. The graph on the left represents the speciation profiles.

f. ATP⁻ titration

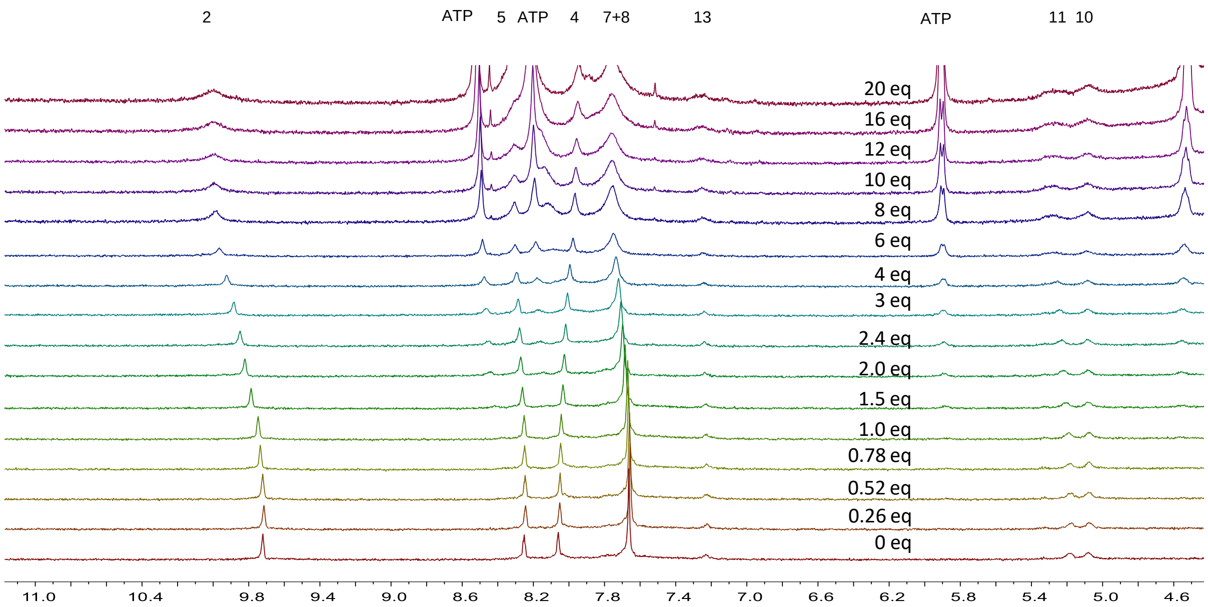

Figure S46. Partial ^{1}H-NMR (300 MHz) changes observed for the host H_8-**5**$(BF_4)_8$ in DMSO-d_6 during the addition of ATP⁻.

Table S9. Data values from the titration study of H_8-**5**$(BF_4)_8$ with ATP⁻

H_8-**5**$(BF_4)_8$ M / mol	(NBu_4)ATP M / mol	Eq (NBu_4)ATP	δ_{H2} [ppm]	$\Delta\delta_{H2}$ [ppm]
$5.14{\cdot}10^{-4}$	0.00	0.00	9.714	0.000
$5.14{\cdot}10^{-4}$	$1.32{\cdot}10^{-4}$	0.27	9.715	0.001
$5.14{\cdot}10^{-4}$	$2.62{\cdot}10^{-4}$	0.53	9.721	0.007
$5.14{\cdot}10^{-4}$	$3.89{\cdot}10^{-4}$	0.78	9.735	0.021
$5.14{\cdot}10^{-4}$	$5.14{\cdot}10^{-4}$	1.0	9.747	0.033
$5.14{\cdot}10^{-4}$	$7.56{\cdot}10^{-4}$	1.5	9.786	0.072
$5.14{\cdot}10^{-4}$	$9.89{\cdot}10^{-4}$	2.0	9.821	0.107
$5.14{\cdot}10^{-4}$	$1.21{\cdot}10^{-3}$	2.4	9.848	0.134
$5.14{\cdot}10^{-4}$	$1.53\ {\cdot}10^{-3}$	3.1	9.882	0.168
$5.14{\cdot}10^{-4}$	$2.04\ {\cdot}10^{-3}$	4.1	9.922	0.208
$5.14{\cdot}10^{-4}$	$3.00\ {\cdot}10^{-3}$	6.0	9.965	0.251
$5.14{\cdot}10^{-4}$	$4.02{\cdot}10^{-3}$	8.1	9.979	0.265
$5.14{\cdot}10^{-4}$	$5.01\ {\cdot}10^{-3}$	10.1	9.991	0.277
$5.14{\cdot}10^{-4}$	$6.02\ {\cdot}10^{-3}$	12.1	9.989	0.275
$5.14{\cdot}10^{-4}$	$8.01\ {\cdot}10^{-3}$	16.1	9.997	0.283
$5.14{\cdot}10^{-4}$	$1.00\ {\cdot}10^{-2}$	20.1	9.995	0.281

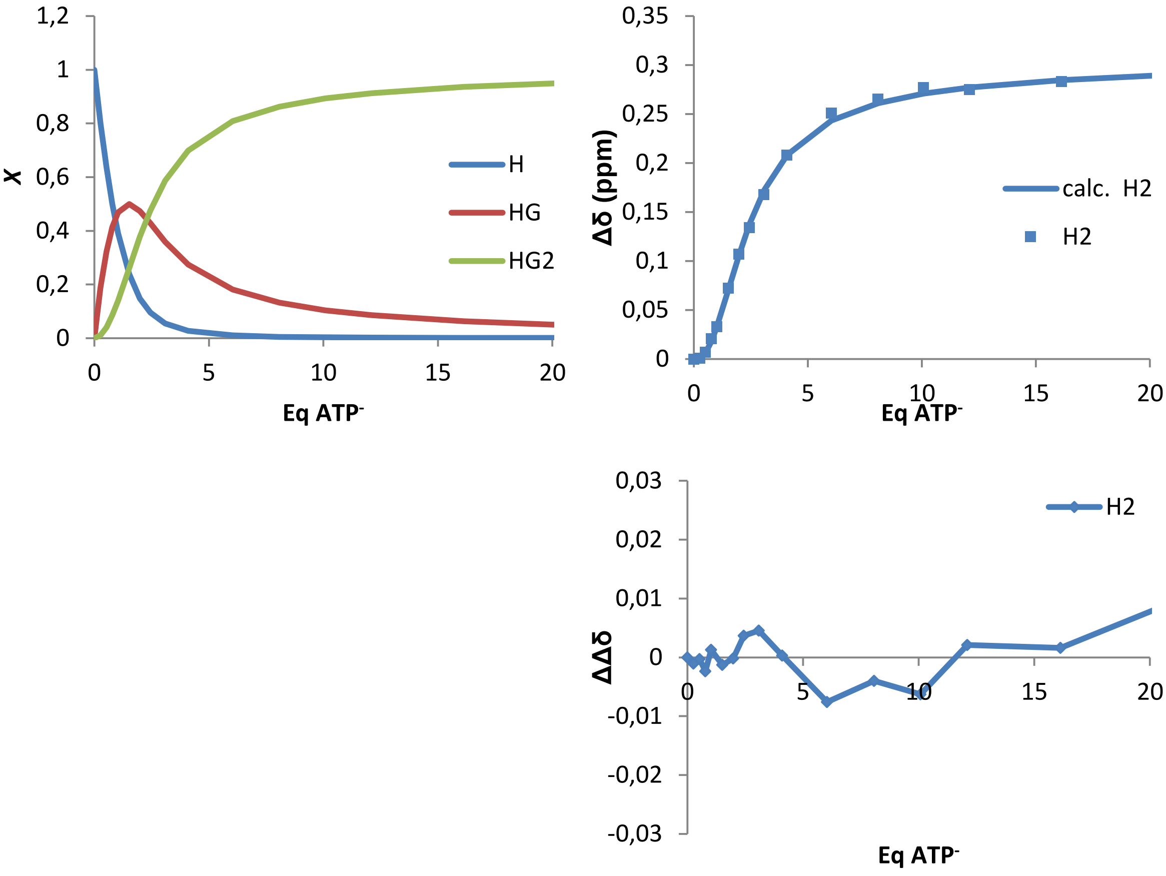

Figure S47. Non-linear least-squares fitting calculated nonlinear regression analysis and assuming a non-cooperative 1:2 (H:G) binding model of the chemical shift changes of H2 during titration experiments of H_8-**5**$(BF_4)_4$ with $ATP^–$. The graph on the left represents the speciation profiles.

5.2. ^{1}H NMR titration experiments with H_4-2$(BF_4)_4$

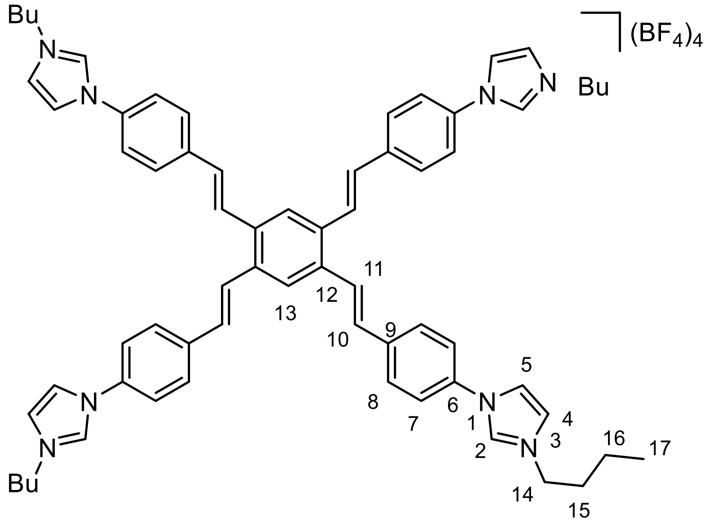

a. Br^{-} titration

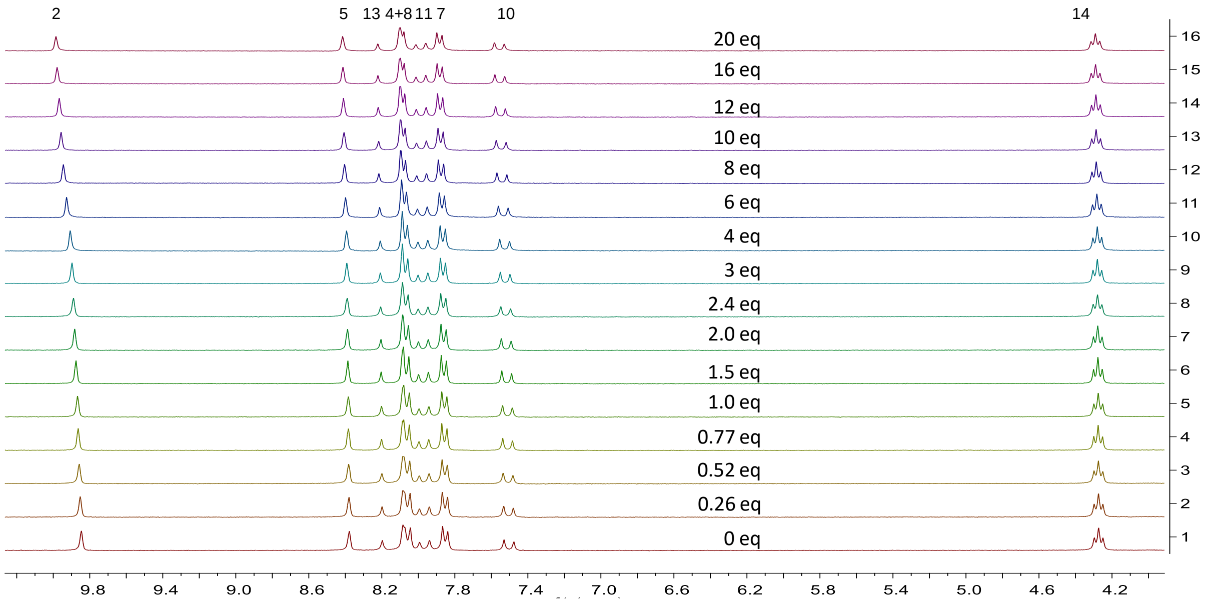

Figure S48. Partial ^{1}H-NMR (300 MHz) changes observed for the host H_4-**2**$(BF_4)_4$ in DMSO-d_6 during the addition of Br^{-}.

Table S10. Data values from the titration study of H_4-**2**$(BF_4)_4$ with Br^-

H_4-**2**$(BF_4)_4$ M / mol	NBu_4Br M / mol	Eq NBu_4Br	δ_{H2} [ppm]	δ_{H5} [ppm]	$\Delta\delta_{H2}$ [ppm]	$\Delta\delta_{H5}$ [ppm]
$2.02 \cdot 10^{-3}$	0.00	0.00	9.846	8.378	0.000	0.000
$2.02 \cdot 10^{-3}$	$5.30 \cdot 10^{-4}$	0.26	9.852	8.380	0.006	0.002
$2.02 \cdot 10^{-3}$	$1.05 \cdot 10^{-3}$	0.52	9.857	8.381	0.011	0.003
$2.02 \cdot 10^{-3}$	$1.56 \cdot 10^{-3}$	0.77	9.863	8.383	0.017	0.005
$2.02 \cdot 10^{-3}$	$2.06 \cdot 10^{-3}$	1.0	9.867	8.383	0.021	0.005
$2.02 \cdot 10^{-3}$	$3.03 \cdot 10^{-3}$	1.5	9.875	8.386	0.029	0.008
$2.02 \cdot 10^{-3}$	$3.97 \cdot 10^{-3}$	2.0	9.882	8.387	0.036	0.009
$2.02 \cdot 10^{-3}$	$4.87 \cdot 10^{-3}$	2.4	9.889	8.389	0.043	0.011
$2.02 \cdot 10^{-3}$	$6.16 \cdot 10^{-3}$	3.0	9.897	8.392	0.051	0.014
$2.02 \cdot 10^{-3}$	$8.17 \cdot 10^{-3}$	4.1	9.907	8.393	0.061	0.015
$2.02 \cdot 10^{-3}$	$1.14 \cdot 10^{-2}$	5.7	9.927	8.399	0.081	0.021
$2.02 \cdot 10^{-3}$	$1.61 \cdot 10^{-2}$	8.0	9.945	8.404	0.099	0.026
$2.02 \cdot 10^{-3}$	$2.01 \cdot 10^{-2}$	10.0	9.957	8.407	0.111	0.029
$2.02 \cdot 10^{-3}$	$2.41 \cdot 10^{-2}$	12.0	9.967	8.410	0.121	0.032
$2.02 \cdot 10^{-3}$	$3.21 \cdot 10^{-2}$	15.9	9.979	8.413	0.133	0.035
$2.02 \cdot 10^{-3}$	$4.02 \cdot 10^{-2}$	19.9	9.985	8.415	0.139	0.037

Plot with a non-cooperative 1:2 (H:G) binding model:

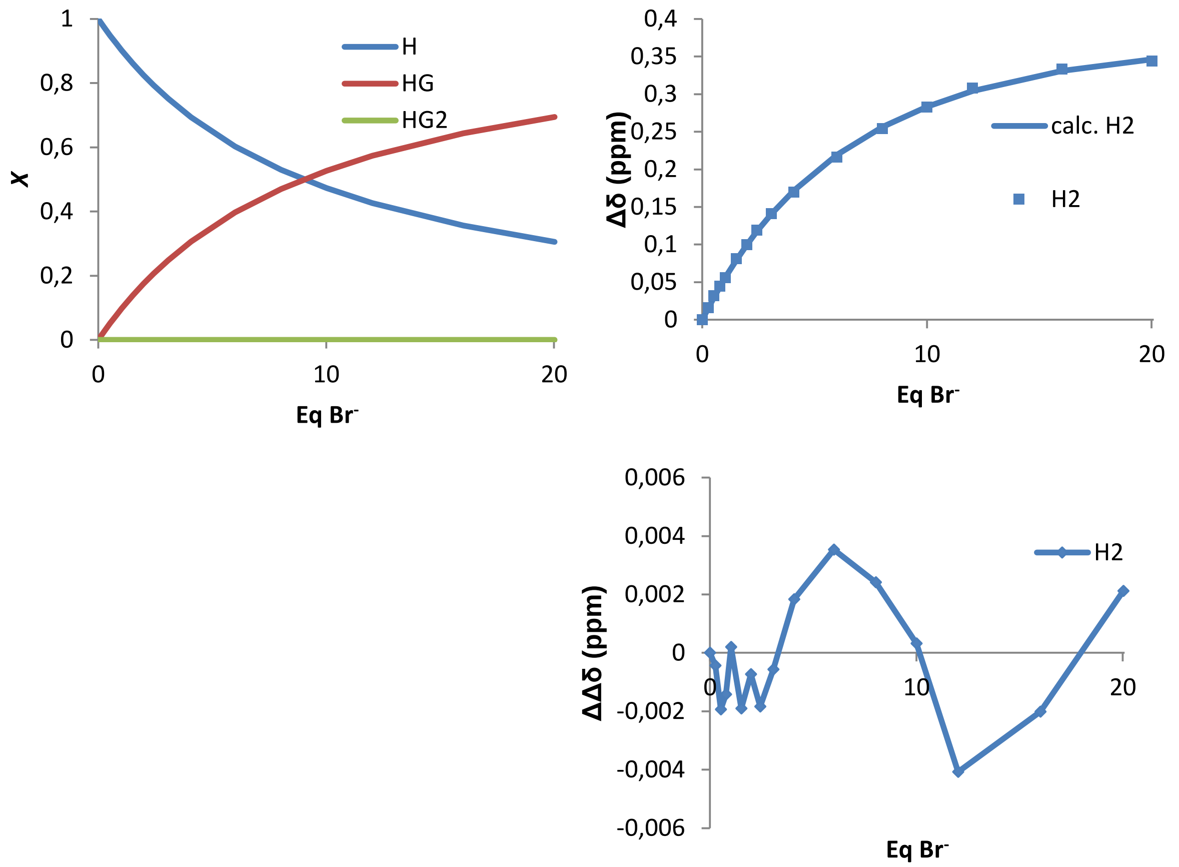

Figure S49. Non-linear least-squares fitting calculated nonlinear regression analysis and assuming a non-cooperative 1:2 (H:G) binding model of the chemical shift changes of H2 and H5 during titration experiments of H_4-**2**$(BF_4)_4$ with Br^-. The graph on the left represents the speciation profiles.

Plot without parameter restrictions:

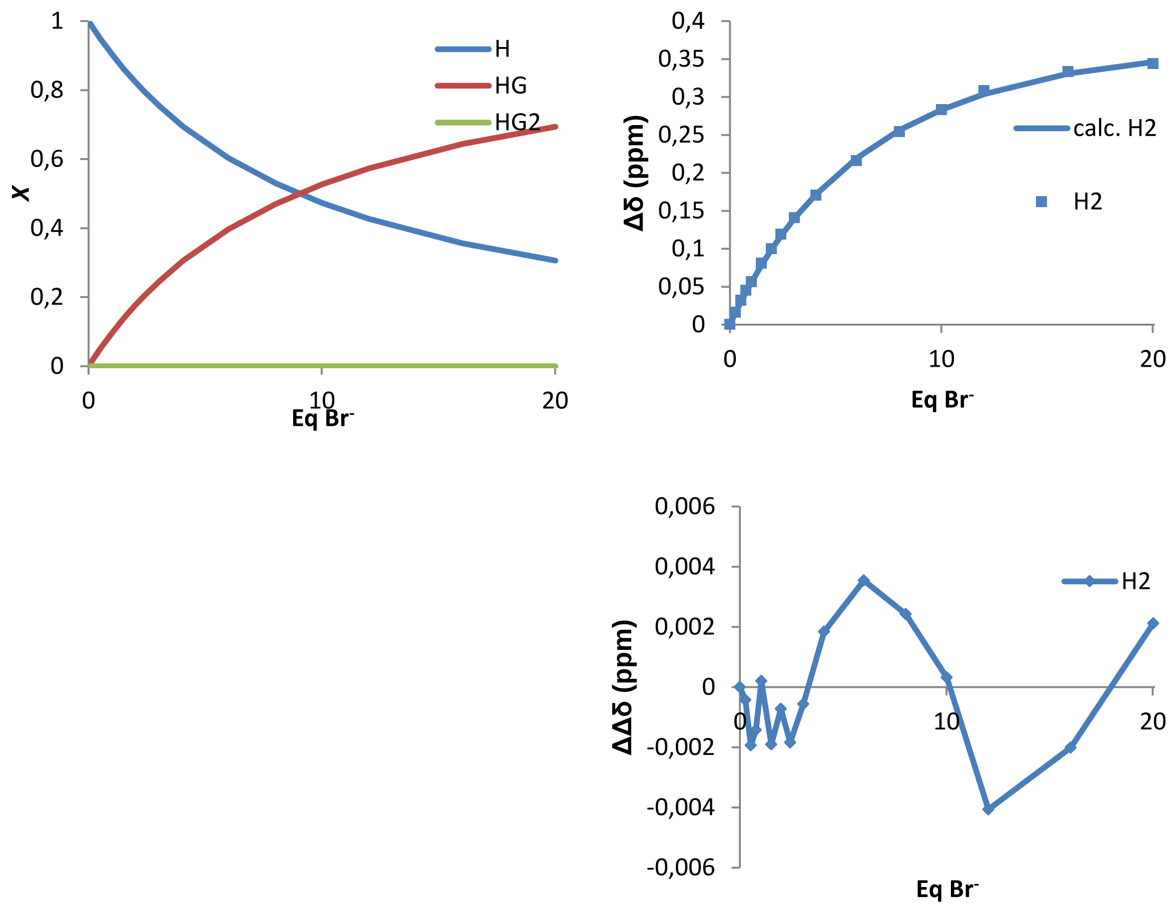

Figure S50. Non-linear least-squares fitting calculated without parameter restriction 1:2 (H:G) binding model of the chemical shift changes of H2 during titration experiments of **2**$(BF_4)_4$ with Br^-. The Figure on the left represents the speciation profiles.

b. benzoate titration

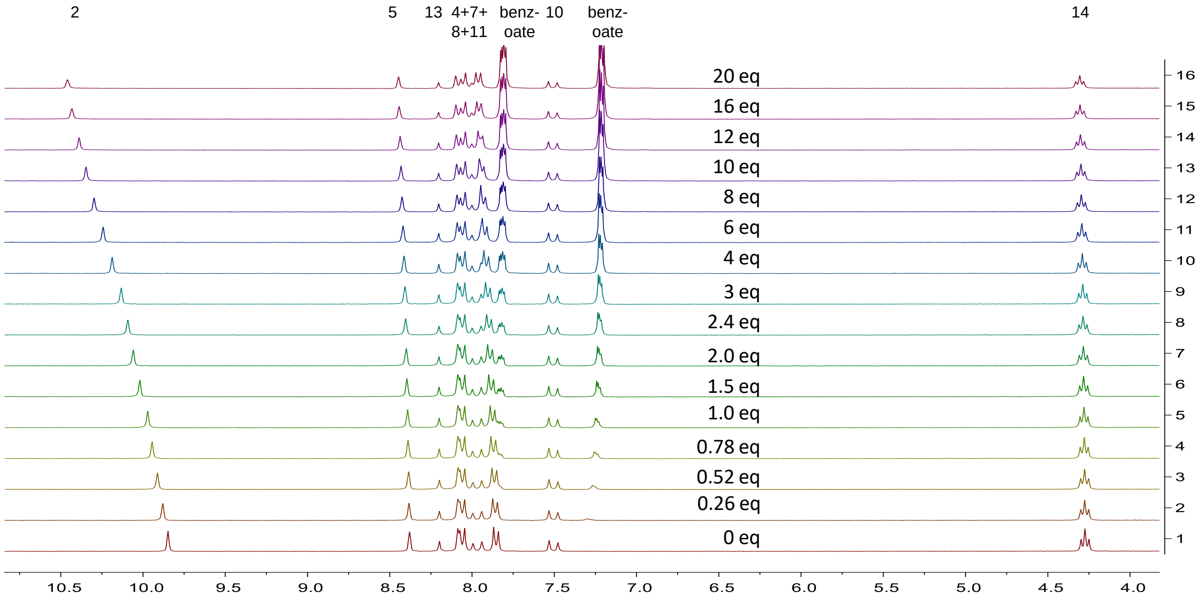

Figure S51. Partial ^{1}H-NMR (300 MHz) changes observed for the host H_4-**2**$(BF_4)_4$ in DMSO-d_6 during the addition of benzoate.

Table S11. Data values from the titration study of H_4-**2**$(BF_4)_4$ with benzoate

H_4-**2**$(BF_4)_4$ M / mol	NBu_4PhCOO M / mol	Eq NBu_4PhCOO	δ_{H2} [ppm]	δ_{H7} [ppm]	$\Delta\delta_{H2}$ [ppm]	$\Delta\delta_{H7}$ [ppm]
$2.02 \cdot 10^{-3}$	0.00	0.00	9.848	7.854	0.000	0.000
$2.02 \cdot 10^{-3}$	$5.33 \cdot 10^{-4}$	0.26	9.880	7.860	0.032	0.006
$2.02 \cdot 10^{-3}$	$1.06 \cdot 10^{-3}$	0.52	9.912	7.864	0.064	0.010
$2.02 \cdot 10^{-3}$	$1.57 \cdot 10^{-3}$	0.78	9.944	7.871	0.096	0.017
$2.02 \cdot 10^{-3}$	$2.07 \cdot 10^{-3}$	1.0	9.971	7.876	0.123	0.022
$2.02 \cdot 10^{-3}$	$3.05 \cdot 10^{-3}$	1.5	10.019	7.884	0.171	0.030
$2.02 \cdot 10^{-3}$	$3.99 \cdot 10^{-3}$	2.0	10.059	7.891	0.211	0.037
$2.02 \cdot 10^{-3}$	$4.90 \cdot 10^{-3}$	2.4	10.092	7.897	0.244	0.043
$2.02 \cdot 10^{-3}$	$6.20 \cdot 10^{-3}$	3.1	10.132	7.904	0.284	0.050
$2.02 \cdot 10^{-3}$	$8.22 \cdot 10^{-3}$	4.1	10.188	7.914	0.340	0.060
$2.02 \cdot 10^{-3}$	$1.21 \cdot 10^{-2}$	6.0	10.242	7.924	0.394	0.070
$2.02 \cdot 10^{-3}$	$1.62 \cdot 10^{-2}$	8.0	10.298	7.933	0.450	0.079
$2.02 \cdot 10^{-3}$	$2.02 \cdot 10^{-2}$	10.0	10.346	7.941	0.498	0.087
$2.02 \cdot 10^{-3}$	$2.43 \cdot 10^{-2}$	12.0	10.389	7.949	0.541	0.095
$2.02 \cdot 10^{-3}$	$3.23 \cdot 10^{-2}$	16.0	10.432	7.958	0.584	0.104
$2.02 \cdot 10^{-3}$	$4.04 \cdot 10^{-2}$	20.0	10.460	7.962	0.612	0.108

Plot with a non-cooperative 1:2 (H:G) binding model:

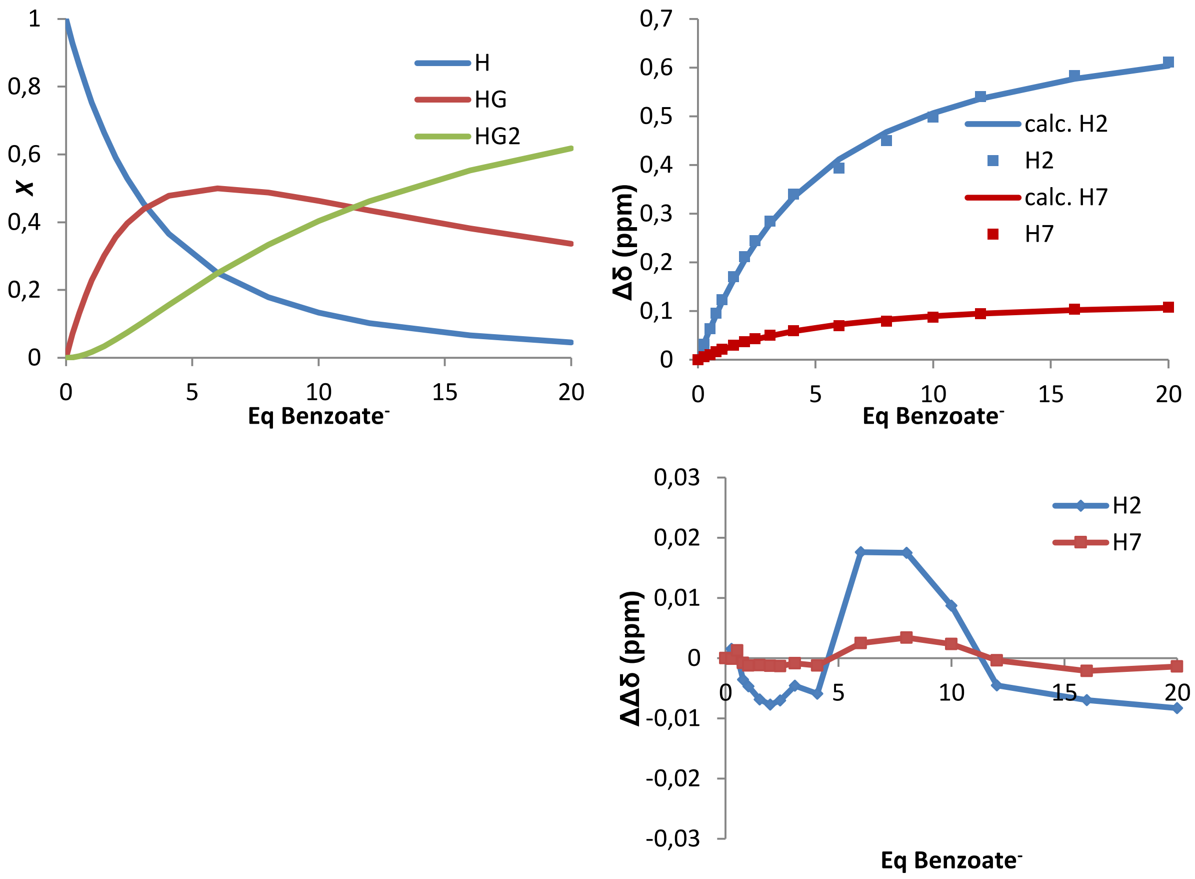

Figure S52. Non-linear least-squares fitting calculated nonlinear regression analysis and assuming a non-cooperative 1:2 (H:G) binding model of the chemical shift changes of H2 and H7 during titration experiments of H_4-**2**$(BF_4)_4$ with benzoate$^-$. The graph on the left represents the speciation profiles.

Plot without parameter restrictions:

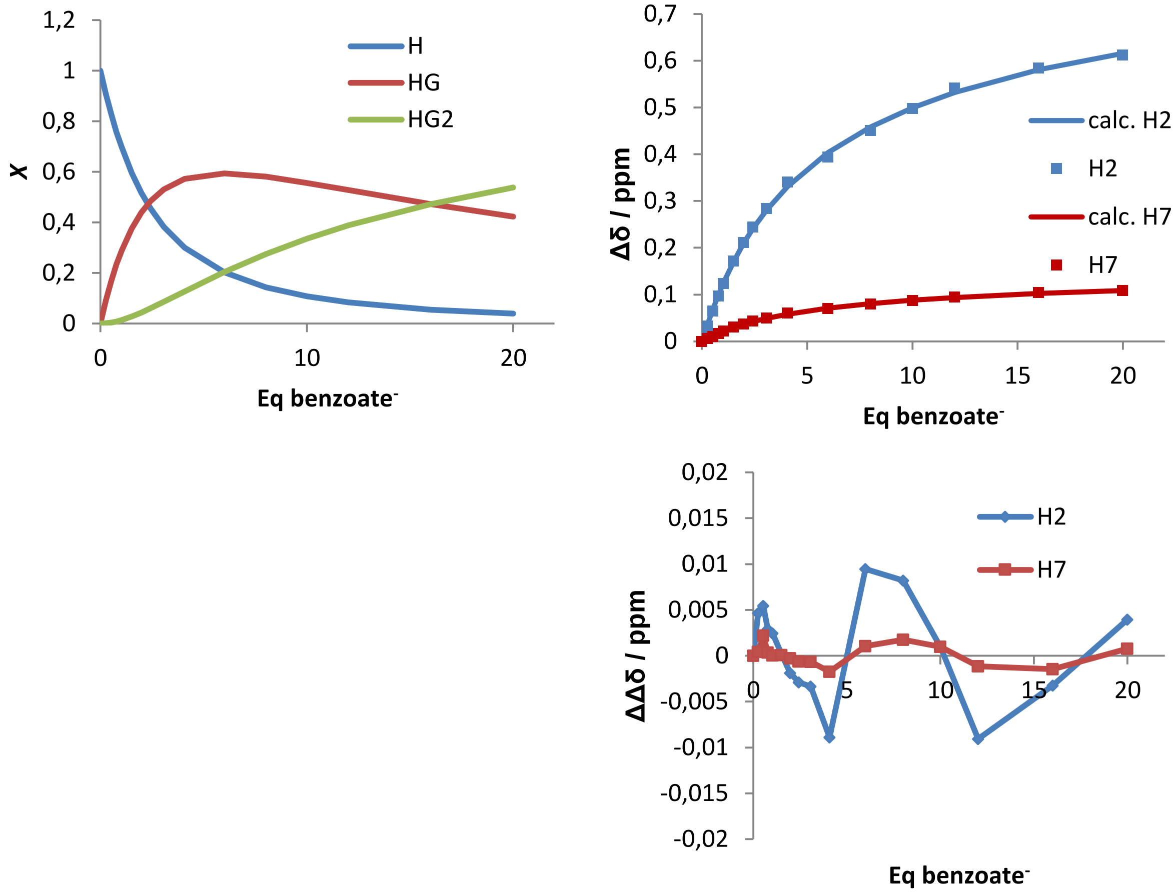

Figure 53. Non-linear least-squares fitting calculated without parameter restriction 1:2 (H:G) binding model of the chemical shift changes of H2 and H7 during titration experiments of H_4-**2**$(BF_4)_4$ with benzoate$^-$. The Figure on the left represents the speciation profiles.

c. Cl⁻ titration

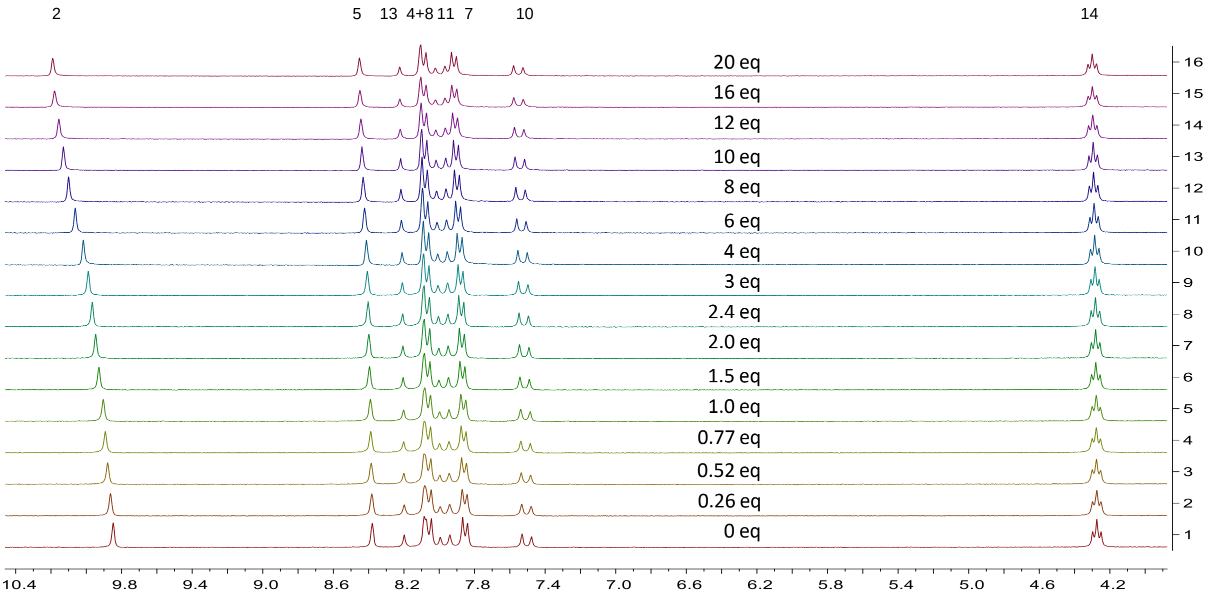

Figure S54. Partial ^{1}H-NMR (300 MHz) changes observed for the host H_4-**2**$(BF_4)_4$ in DMSO-d_6 during the addition Cl^-.

Table S12. Data values from the titration study of H_4-**2**$(BF_4)_4$ with Cl^-

H_4-**2**$(BF_4)_4$ M / mol	NBu_4Cl M / mol	Eq NBu_4Cl	δ_{H2} [ppm]	$\Delta\delta_{H2}$ [ppm]
$2.02\cdot10^{-3}$	0.00	0.00	9.847	0.000
$2.02\cdot10^{-3}$	$5.32\cdot10^{-4}$	0.26	9.863	0.016
$2.02\cdot10^{-3}$	$1.05\cdot10^{-3}$	0.52	9.879	0.032
$2.02\cdot10^{-3}$	$1.56\cdot10^{-3}$	0.78	9.892	0.045
$2.02\cdot10^{-3}$	$2.07\cdot10^{-3}$	1.0	9.903	0.056
$2.02\cdot10^{-3}$	$3.04\cdot10^{-3}$	1.5	9.928	0.081
$2.02\cdot10^{-3}$	$3.98\cdot10^{-3}$	2.0	9.947	0.100
$2.02\cdot10^{-3}$	$4.88\cdot10^{-3}$	2.4	9.966	0.119
$2.02\cdot10^{-3}$	$6.18\cdot10^{-3}$	3.1	9.988	0.141
$2.02\cdot10^{-3}$	$8.19\cdot10^{-3}$	4.1	10.017	0.170
$2.02\cdot10^{-3}$	$1.21\cdot10^{-2}$	6.0	10.063	0.216
$2.02\cdot10^{-3}$	$1.53\cdot10^{-2}$	7.6	10.101	0.254
$2.02\cdot10^{-3}$	$2.01\cdot10^{-2}$	10.0	10.130	0.283
$2.02\cdot10^{-3}$	$2.42\cdot10^{-2}$	12.0	10.155	0.308
$2.02\cdot10^{-3}$	$3.22\cdot10^{-2}$	15.9	10.180	0.333
$2.02\cdot10^{-3}$	$4.03\cdot10^{-2}$	19.9	10.191	0.344

Plot with a non-cooperative 1:2 (H:G) binding model:

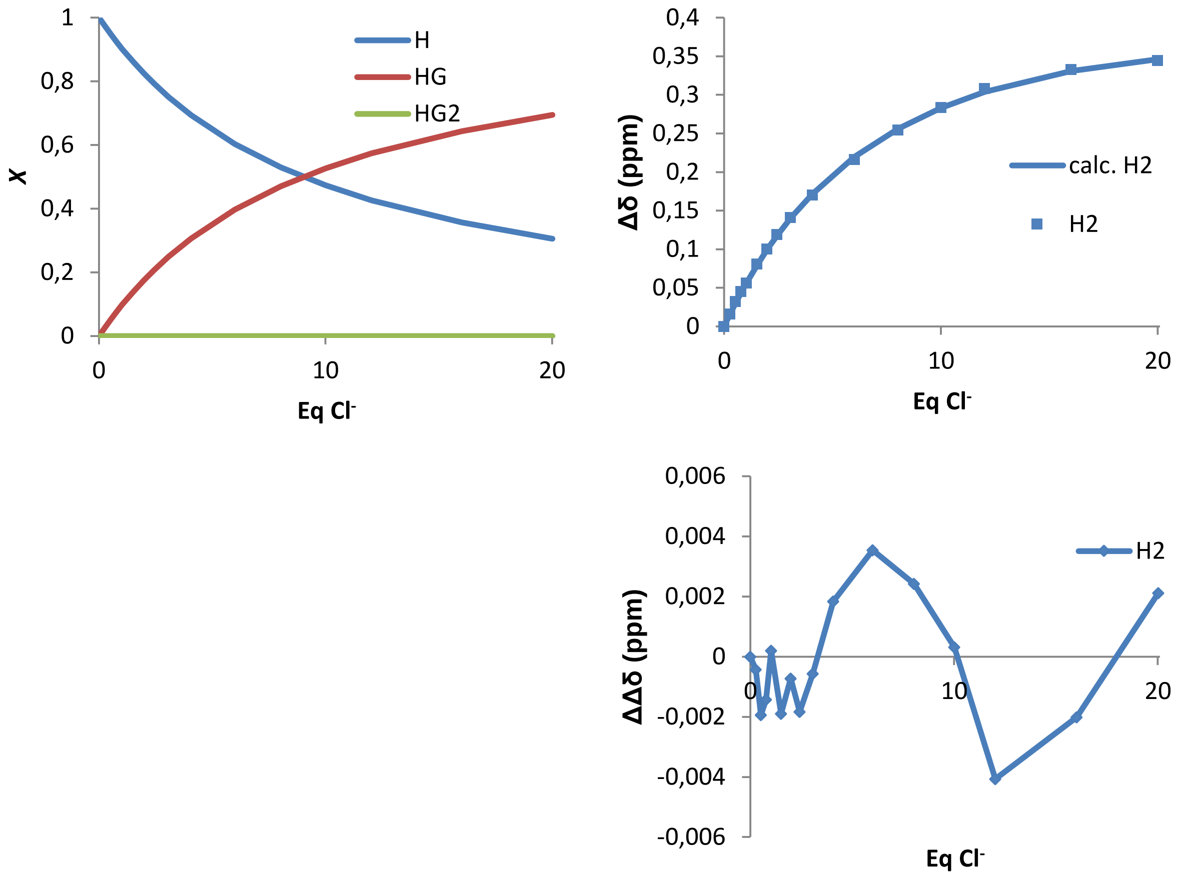

Figure S55. Non-linear least-squares fitting calculated nonlinear regression analysis and assuming a non-cooperative 1:2 (H:G) binding model of the chemical shift changes of H2 during titration experiments of H_4-**2**$(BF_4)_4$ with Cl^-. The graph on the left represents the speciation profiles.

Plot without parameter restrictions:

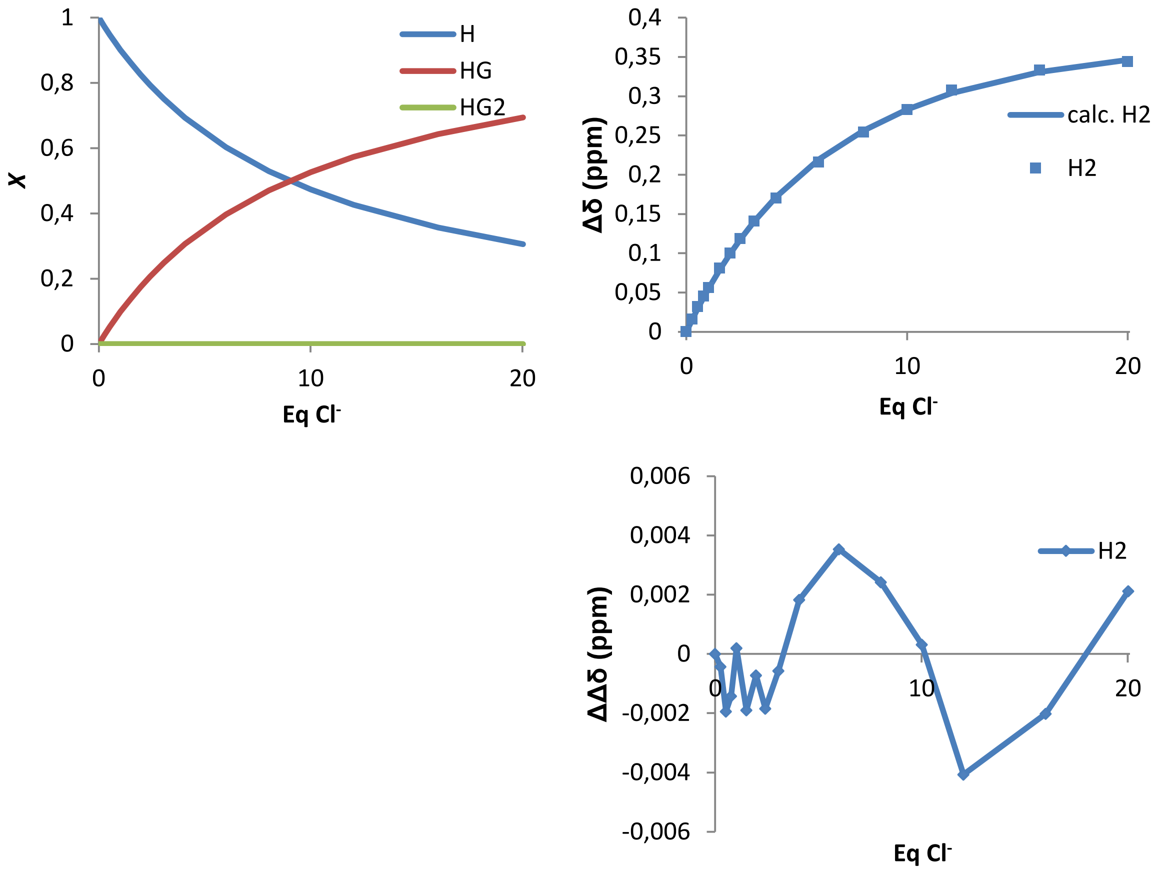

Figure S56. Non-linear least-squares fitting calculated without parameter restriction 1:2 (H:G) binding model of the chemical shift changes of H2 during titration experiments of H_4-**2**$(BF_4)_4$ with Cl^-. The Figure on the left represents the speciation profiles.

d. NO_3^- titration

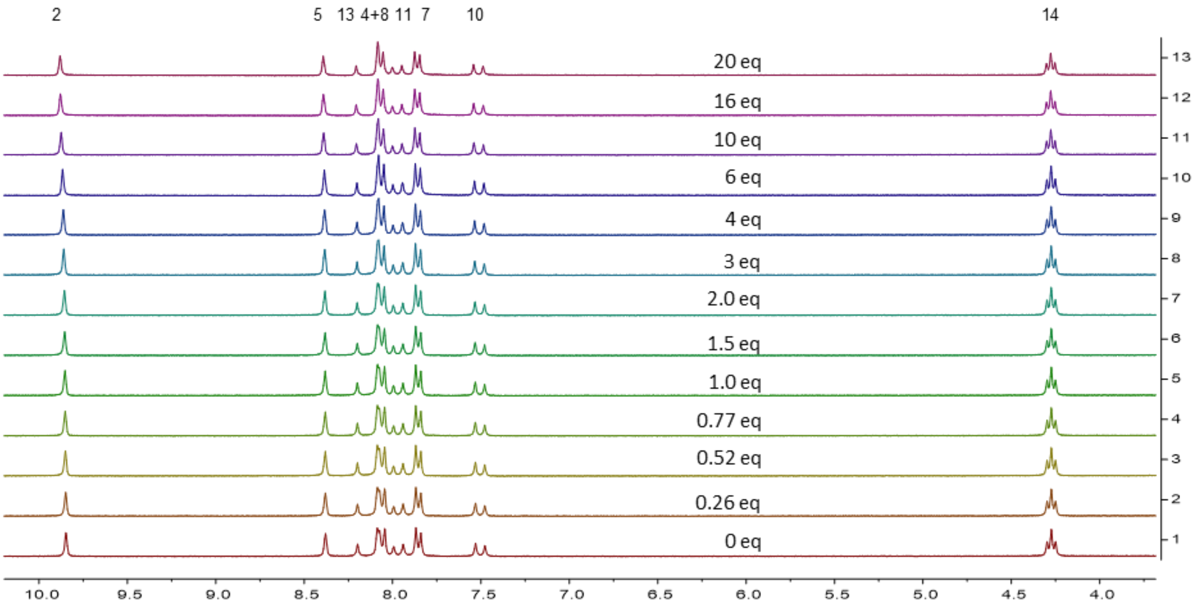

Figure S57. Partial ^{1}H-NMR (300 MHz) changes observed for the host H_4-**2**$(BF_4)_4$ in DMSO-d_6 during the addition NO_3^-.

Table S13. Data values from the titration study of H_4-**2**$(BF_4)_4$ with NO_3^-

H_4-**2**$(BF_4)_4$ M / mol	NBu_4NO_3 M / mol	Eq NBu_4NO_3	δ_{H2} [ppm]	$\Delta\delta_{H2}$ [ppm]
$2.02 \cdot 10^{-3}$	0.00	0.00	9.848	0
$2.02 \cdot 10^{-3}$	$5.31 \cdot 10^{-4}$	0.26	9.849	0.001
$2.02 \cdot 10^{-3}$	$1.05 \cdot 10^{-3}$	0.52	9.851	0.003
$2.02 \cdot 10^{-3}$	$1.56 \cdot 10^{-3}$	0.77	9.852	0.004
$2.02 \cdot 10^{-3}$	$2.06 \cdot 10^{-3}$	1.0	9.853	0.005
$2.02 \cdot 10^{-3}$	$3.03 \cdot 10^{-3}$	1.5	9.854	0.006
$2.02 \cdot 10^{-3}$	$3.97 \cdot 10^{-3}$	2.0	9.856	0.008
$2.02 \cdot 10^{-3}$	$6.17 \cdot 10^{-3}$	3.0	9.86	0.012
$2.02 \cdot 10^{-3}$	$8.18 \cdot 10^{-3}$	4.0	9.862	0.014
$2.02 \cdot 10^{-3}$	$1.21 \cdot 10^{-2}$	6.0	9.867	0.019
$2.02 \cdot 10^{-3}$	$2.01 \cdot 10^{-2}$	10.0	9.875	0.027
$2.02 \cdot 10^{-3}$	$3.21 \cdot 10^{-2}$	16.0	9.879	0.031
$2.02 \cdot 10^{-3}$	$4.02 \cdot 10^{-2}$	19.9	9.881	0.033

Plot with a non-cooperative 1:2 (H:G) binding model:

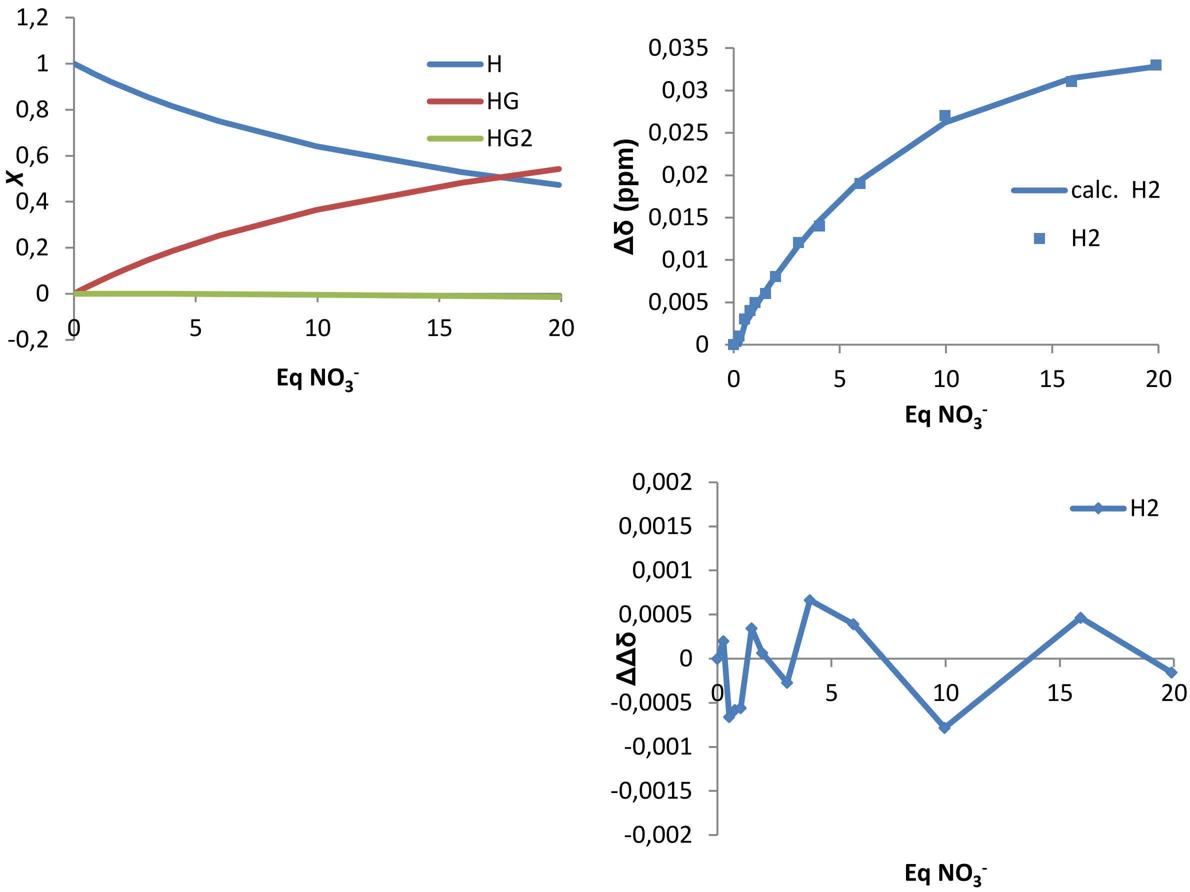

Figure S58. Non-linear least-squares fitting calculated nonlinear regression analysis and assuming a non-cooperative 1:2 (H:G) binding model of the chemical shift changes of H2 during titration experiments of H_4-**2**$(BF_4)_4$ with NO_3^-. The graph on the left represents the speciation profiles.

Plot without parameter restrictions:

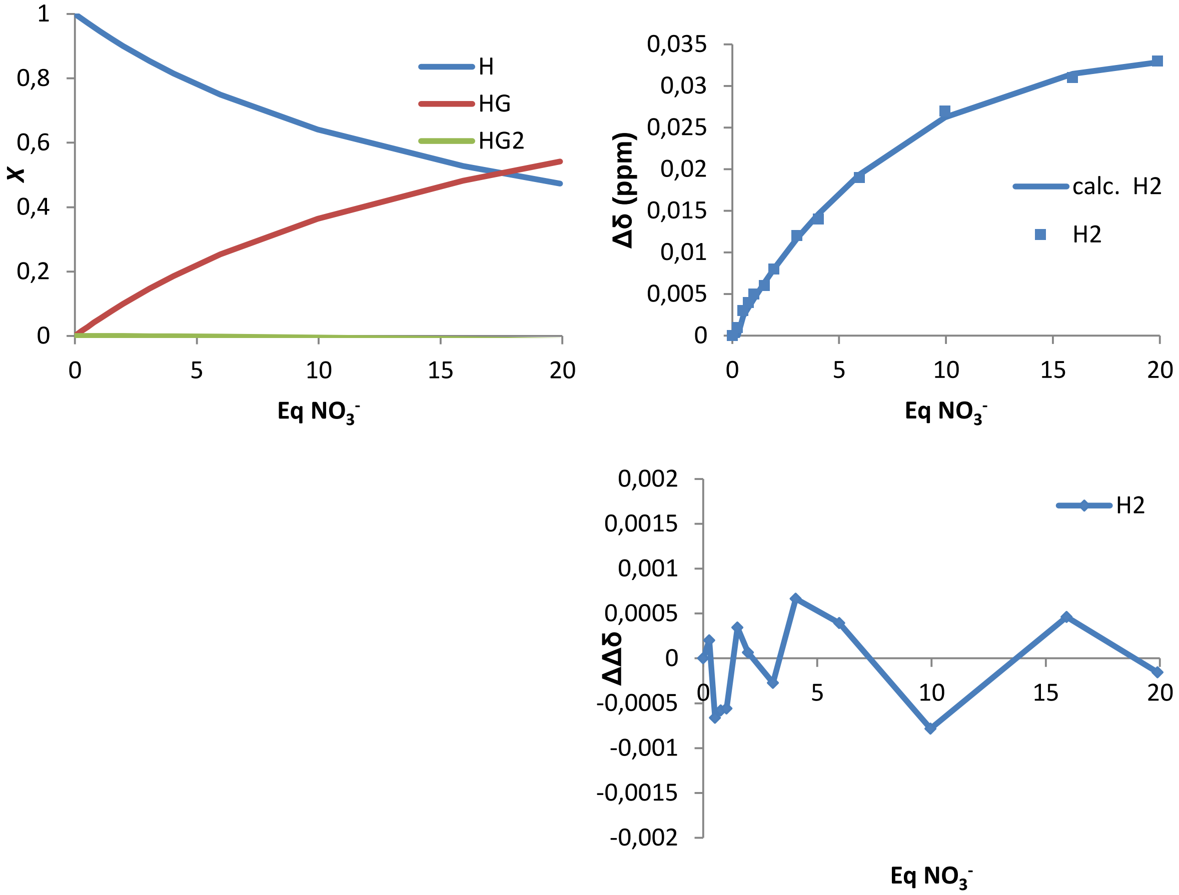

Figure S59. Non-linear least-squares fitting calculated without parameter restriction 1:2 (H:G) binding model of the chemical shift changes of H2 during titration experiments of H_4-**2**$(BF_4)_4$ with NO_3^-. The graph on the left represents the speciation profiles.

e. ibuprofen⁻ titration

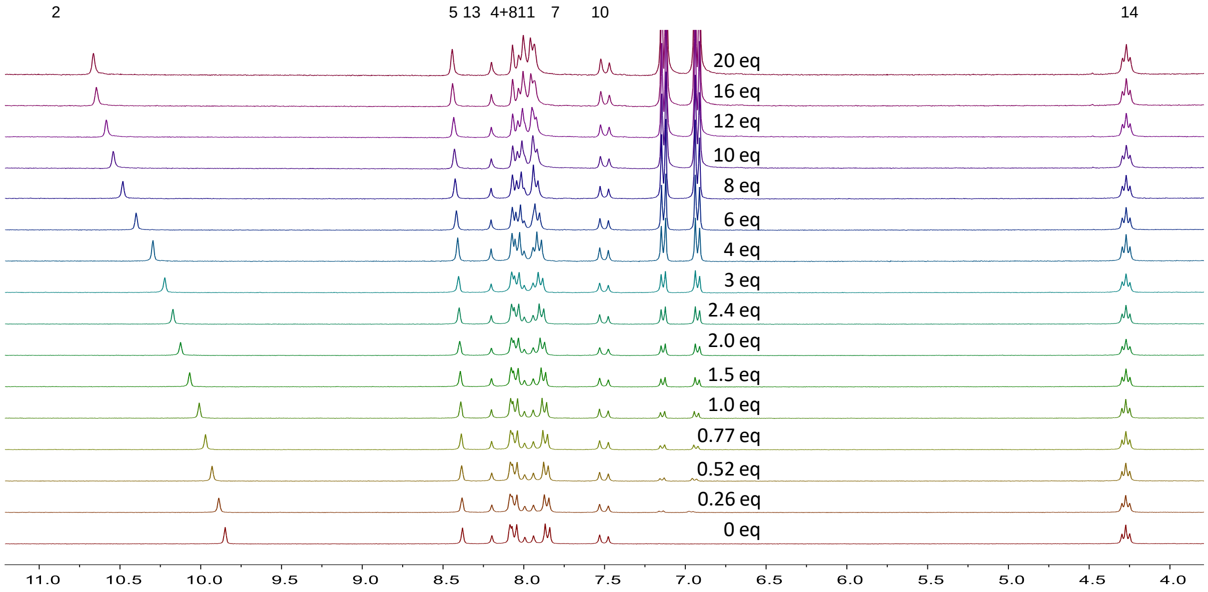

Figure S60. Partial ^{1}H-NMR (300 MHz) changes observed for the host H_4-**2**$(BF_4)_4$ in DMSO-d_6 during the addition ibuprofen⁻.

Table S14. Data values from the titration study of H_4-**2**$(BF_4)_4$ with ibuprofen⁻

H_4-**2**$(BF_4)_4$ M / mol	(NBu_4)(ibuprofen) M / mol	Eq NBu_4(ibuprofen)	δ_{H2} [ppm]	δ_{H5} [ppm]	δ_{H7} [ppm]	$\Delta\delta_{H5}$ [ppm]	$\Delta\delta_{H2}$ [ppm]	$\Delta\delta_{H7}$ [ppm]
$2.02 \cdot 10^{-3}$	0.00	0.00	9.849	8.379	7.854	0.000	0.000	0.000
$2.02 \cdot 10^{-3}$	$5.22 \cdot 10^{-4}$	0.26	9.888	8.382	7.859	0.003	0.039	0.005
$2.02 \cdot 10^{-3}$	$1.03 \cdot 10^{-3}$	0.52	9.930	8.385	7.863	0.006	0.081	0.009
$2.02 \cdot 10^{-3}$	$1.54 \cdot 10^{-3}$	0.76	9.970	8.387	7.868	0.008	0.121	0.014
$2.02 \cdot 10^{-3}$	$2.02 \cdot 10^{-3}$	1.0	10.009	8.390	7.873	0.011	0.160	0.019
$2.02 \cdot 10^{-3}$	$2.99 \cdot 10^{-3}$	1.5	10.068	8.393	7.879	0.014	0.219	0.025
$2.02 \cdot 10^{-3}$	$3.91 \cdot 10^{-3}$	2.0	10.125	8.397	7.885	0.018	0.276	0.031
$2.02 \cdot 10^{-3}$	$4.80 \cdot 10^{-3}$	2.4	10.172	8.400	7.891	0.021	0.323	0.037
$2.02 \cdot 10^{-3}$	$6.07 \cdot 10^{-3}$	3.0	10.222	8.404	7.897	0.025	0.373	0.043
$2.02 \cdot 10^{-3}$	$8.04 \cdot 10^{-3}$	4.0	10.296	8.409	7.905	0.030	0.447	0.051
$2.02 \cdot 10^{-3}$	$1.18 \cdot 10^{-2}$	5.9	10.400	8.417	7.917	0.038	0.551	0.063
$2.02 \cdot 10^{-3}$	$1.59 \cdot 10^{-2}$	7.9	10.482	8.425	7.927	0.046	0.633	0.073
$2.02 \cdot 10^{-3}$	$1.98 \cdot 10^{-2}$	9.8	10.542	8.429	7.931	0.050	0.693	0.077
$2.02 \cdot 10^{-3}$	$2.38 \cdot 10^{-2}$	11.8	10.585	8.434	7.937	0.055	0.736	0.083
$2.02 \cdot 10^{-3}$	$3.16 \cdot 10^{-2}$	15.6	10.646	8.441	7.945	0.062	0.797	0.091
$2.02 \cdot 10^{-3}$	$2.42 \cdot 10^{-2}$	19.6	10.664	8.443	7.947	0.064	0.815	0.093

Plot with a non-cooperative 1:2 (H:G) binding model

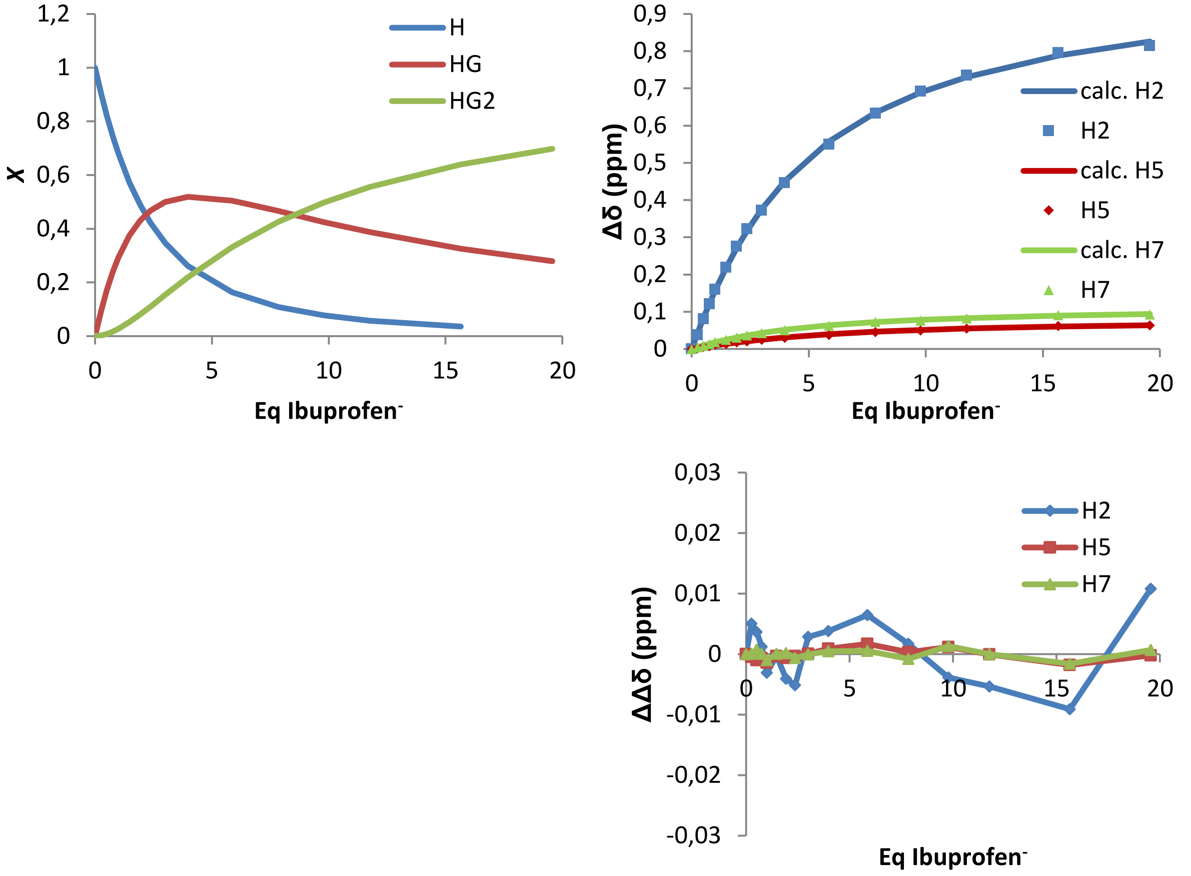

Figure S61. Non-linear least-squares fitting calculated without parameter restriction 1:2 (H:G) binding model of the chemical shift changes of H2, H5 and H7 during titration experiments of H_4-**2**$(BF_4)_4$ with ibuprofen$^-$. The graph on the left represents the speciation profiles.

Plot without parameter restrictions:

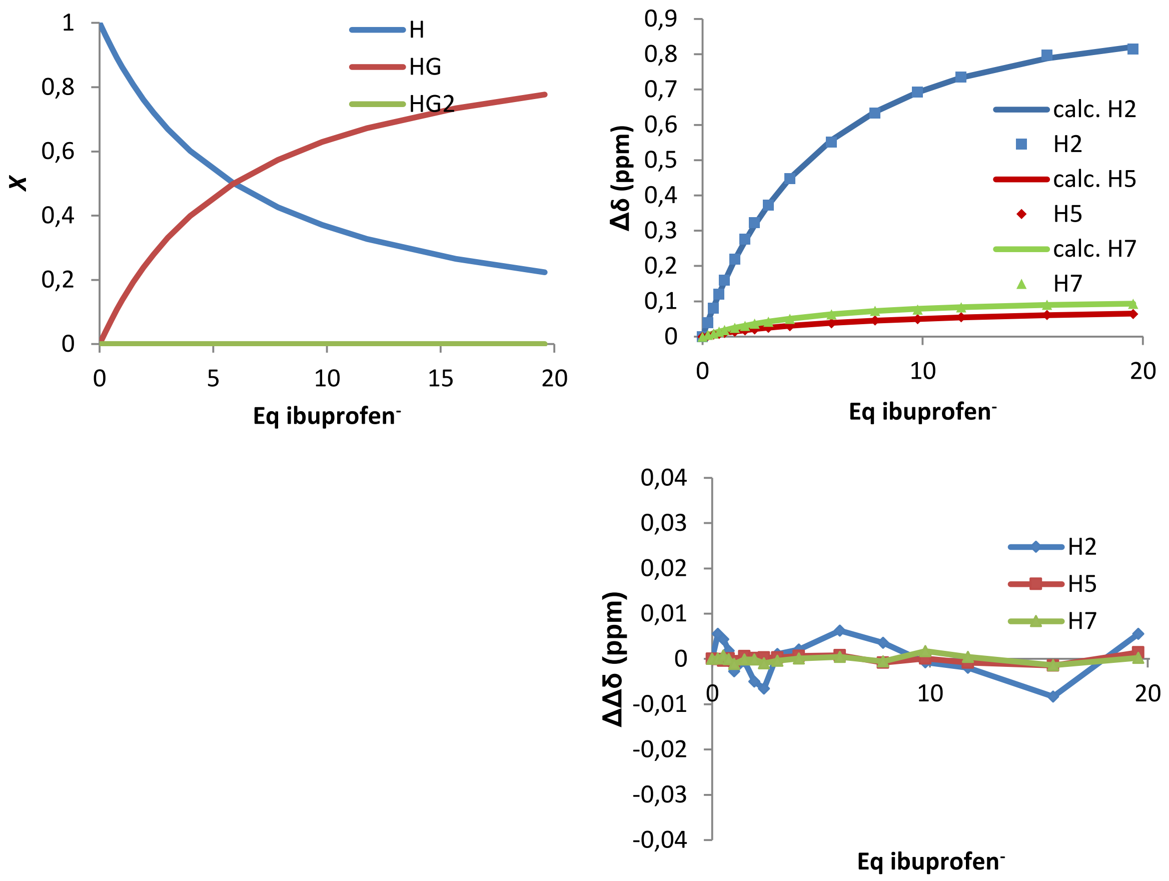

Figure 62. Non-linear least-squares fitting calculated without parameter restriction 1:2 (H:G) binding model of the chemical shift changes of H2, H5 and H7 during titration experiments of **2**$(BF_4)_4$ with ibuprofen$^-$. The Figure on the left represents the speciation profiles.

f. ATP⁻ titration

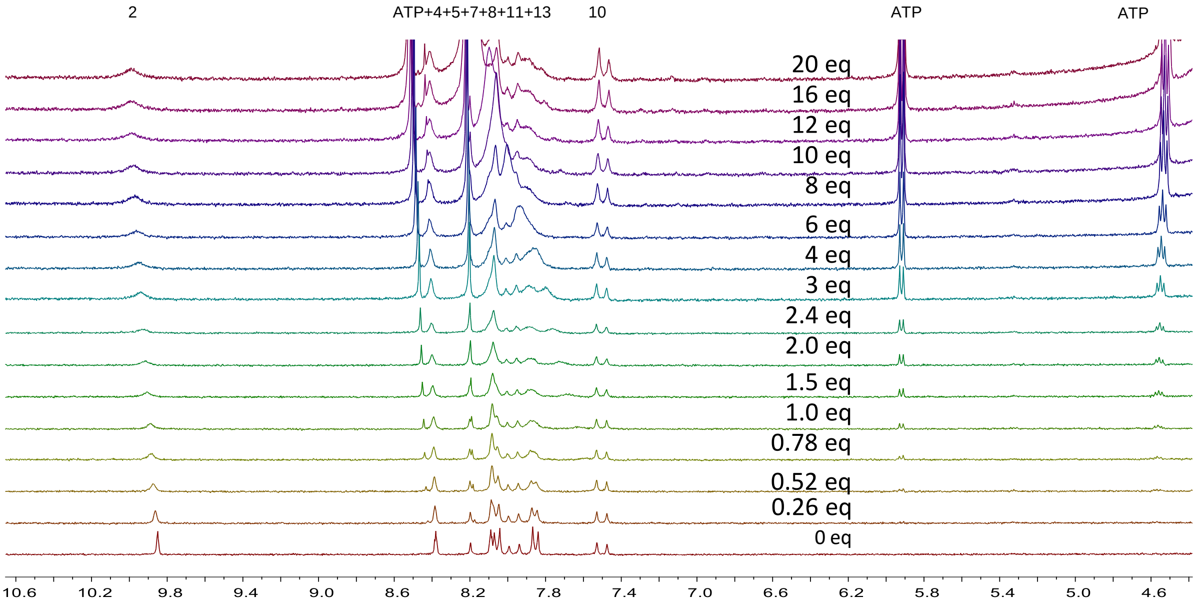

Figure S63. Partial ^{1}H-NMR (300 MHz) changes observed for the host H_4-**2**$(BF_4)_4$ in DMSO-*d*$_6$ during the addition ATP⁻.

Table S15. Data values from the titration study of H_4-**2**$(BF_4)_4$ with ATP⁻

H_4-**2**$(BF_4)_4$ M / mol	$(NBu_4)(ATP)$ M / mol	Eq $NBu_4(ATP)$	δ_{H2} [ppm]	$\Delta\delta_{H2}$ [ppm]
$5.02 \cdot 10^{-4}$	0.00	0.00	9.850	0.000
$5.02 \cdot 10^{-4}$	$5.33 \cdot 10^{-4}$	0.26	9.862	0.012
$5.02 \cdot 10^{-4}$	$1.05 \cdot 10^{-3}$	0.51	9.875	0.025
$5.02 \cdot 10^{-4}$	$1.57 \cdot 10^{-3}$	0.76	9.885	0.035
$5.02 \cdot 10^{-4}$	$2.07 \cdot 10^{-3}$	1.0	9.892	0.042
$5.02 \cdot 10^{-4}$	$3.04 \cdot 10^{-3}$	1.5	9.907	0.057
$5.02 \cdot 10^{-4}$	$3.98 \cdot 10^{-3}$	2.0	9.917	0.067
$5.02 \cdot 10^{-4}$	$4.89 \cdot 10^{-3}$	2.4	9.926	0.076
$5.02 \cdot 10^{-4}$	$6.19 \cdot 10^{-3}$	3.0	9.939	0.089
$5.02 \cdot 10^{-4}$	$8.21 \cdot 10^{-3}$	4.0	9.948	0.098
$5.02 \cdot 10^{-4}$	$1.21 \cdot 10^{-2}$	5.8	9.964	0.114
$5.02 \cdot 10^{-4}$	$1.62 \cdot 10^{-2}$	7.8	9.971	0.121
$5.02 \cdot 10^{-4}$	$2.02 \cdot 10^{-2}$	9.7	9.979	0.129
$5.02 \cdot 10^{-4}$	$2.42 \cdot 10^{-2}$	11.7	9.985	0.135
$5.02 \cdot 10^{-4}$	$3.23 \cdot 10^{-2}$	15.6	9.992	0.142
$5.02 \cdot 10^{-4}$	$4.03 \cdot 10^{-2}$	19.5	9.992	0.142

Plot with a non-cooperative 1:2 (H:G) binding model

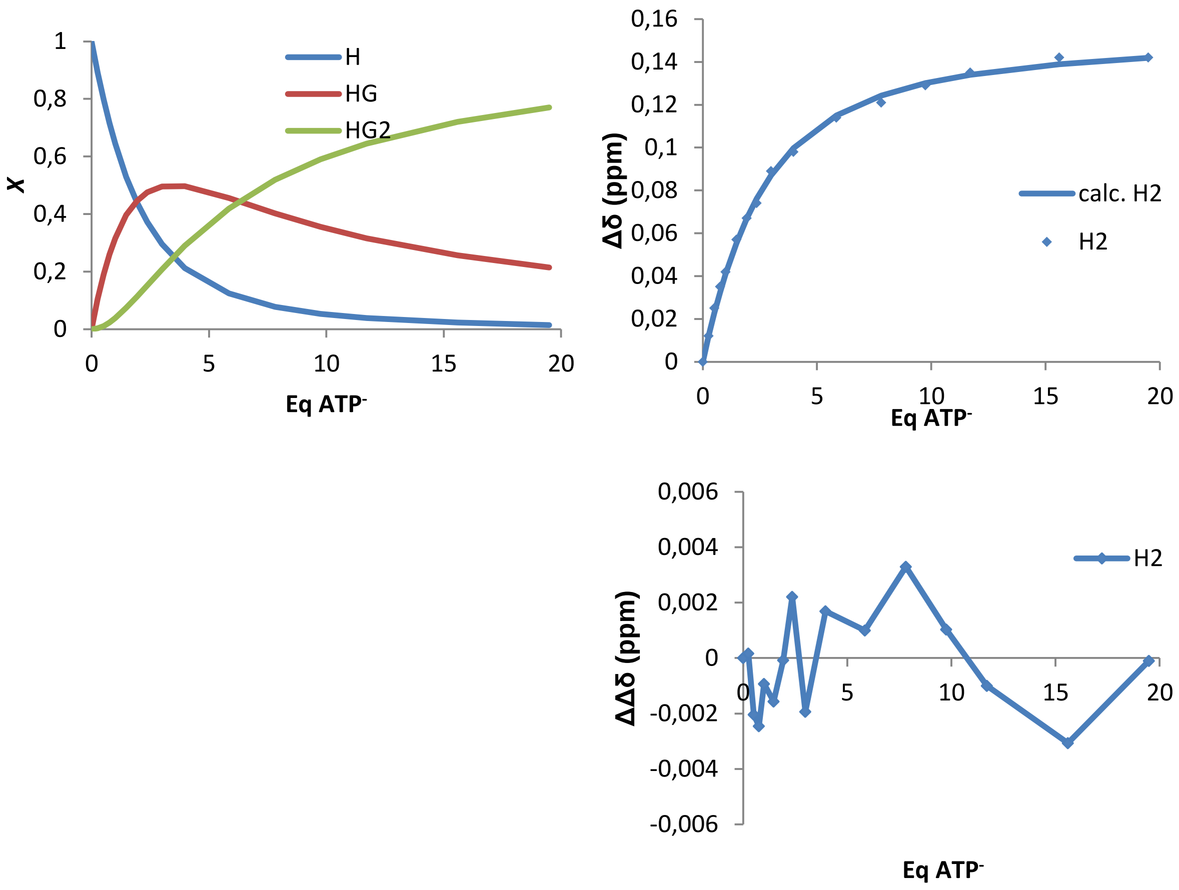

Figure S64. Non-linear least-squares fitting calculated nonlinear regression analysis and assuming a non-cooperative 1:2 (H:G) binding model of the chemical shift changes of H2 during titration experiments of H_4-**2**$(BF_4)_4$ with ATP^{-}. The graph on the left represents the speciation profiles.

Plot without parameter restrictions

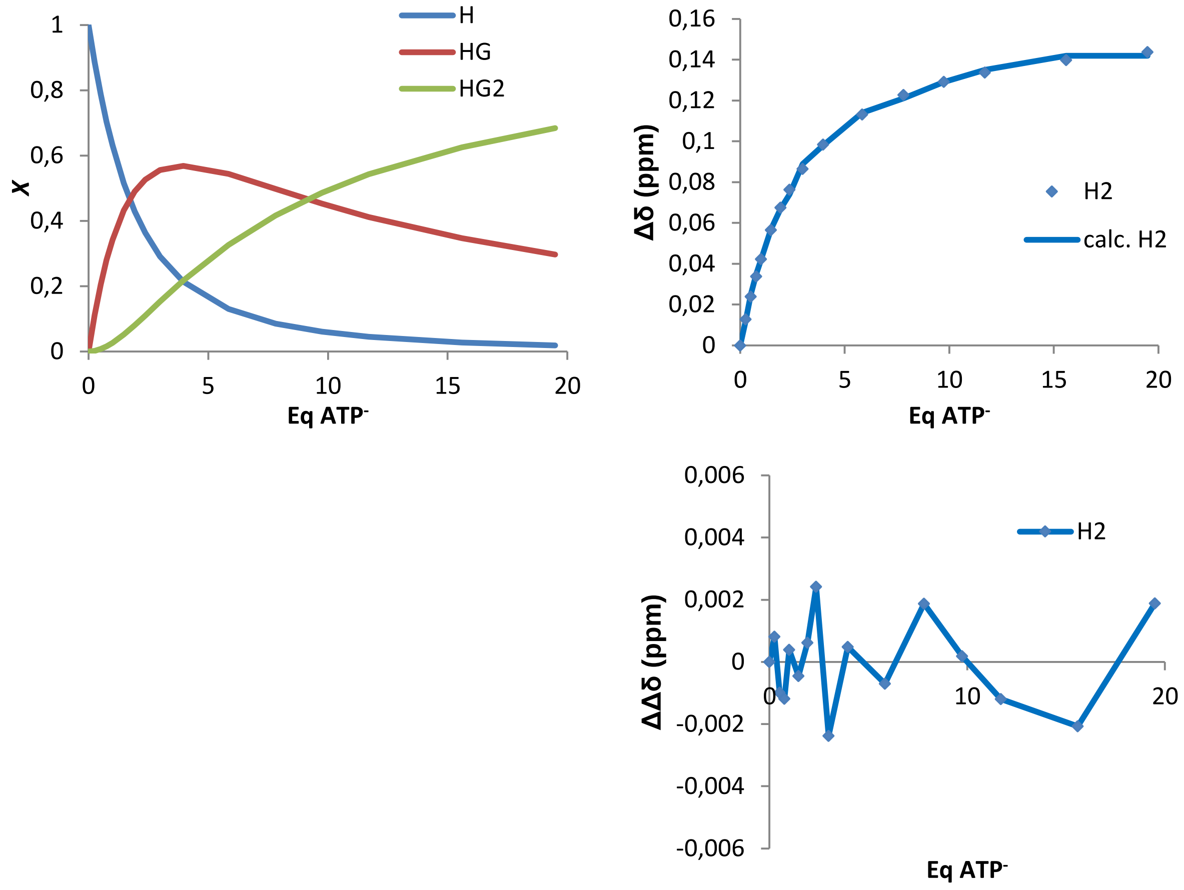

Figure S65. Non-linear least-squares fitting calculated without parameter restriction 1:2 (H:G) binding model of the chemical shift changes of H2 during titration experiments of **2**$(BF_4)_4$ with ATP^-. The Figure on the left represents the speciation profiles.

5.3. Job Plots

The host-guest stoichiometry of compounds H_4-**2**$(BF_4)_4$ and H_8-**5**$(BF_4)_8$ with different anions (Cl^-, Br^-, NO_3^-, benzoate$^-$, ibuprofen$^-$, ATP^-) was tested by ^{1}H NMR spectroscopy (in DMSO-*d*$_6$). Tetrabutylammonium salts were selected as the anion sources. For the Job plots two different stock solutions were prepared; solution A: 2 mM of the host for Cl^-, Br^-, NO_3^-, benzoate$^-$, ibuprofen$^-$ or 0.5 mM of the host for ATP^-; solution B: 2 mM of the host and 53.6 mM of the guest for Cl^-, Br^-, NO_3^-, benzoate$^-$, ibuprofen$^-$ or 0.5 mM of the host and 13.4 mM of ATP^-. The two solutions were combined to give a series of samples with an identical concentration of the host but containing different mole fractions of the two components.

a. H_4-**2**$(BF_4)_4$ with NBu_4ATP

Table S16. Data values of the Job Plot of H_4-**2**$(BF_4)_4$ with $(ATP)^-$

X[H]	δ (ppm)	Δδ (ppm)	X[H]*Δδ (ppm)
1	9.85	0	0
0.80	9.862	0.012	0.0095
0.66	9.875	0.025	0.0166
0.57	9.885	0.035	0.0199
0.50	9.892	0.042	0.0210
0.40	9.907	0.057	0.0231
0.34	9.917	0.067	0.0229
0.30	9.926	0.076	0.0226
0.25	9.939	0.089	0.0223
0.20	9.948	0.098	0.0197
0.15	9.964	0.114	0.0167
0.11	9.971	0.121	0.0137
0.09	9.979	0.129	0.0120
0.08	9.985	0.135	0.0106
0.06	9.992	0.142	0.0086
0.05	9.992	0.142	0.0069

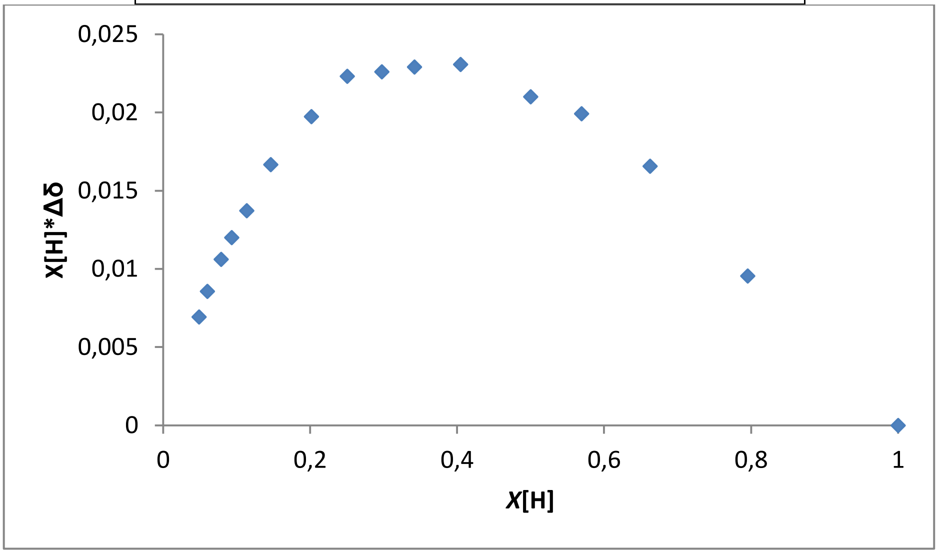

Figure S66. Job plot in DMSO-*d*$_6$ for the interaction of H_4-**2**$(BF_4)_4$ and ATP^- $[H]=5\times10^{-4}$ M).

b. H_4-**2**$(BF_4)_4$ with NBu_4Ibuprofen

Table S17. Data values of the Job plot of H_4-**2**$(BF_4)_4$ with ibuprofen$^-$

X[H]	δ (ppm)	Δδ (ppm)	X[H]*Δδ (ppm)
1.00	9.849	0	0.0000
0.79	9.888	0.039	0.0310
0.66	9.93	0.081	0.0536
0.57	9.97	0.121	0.0688
0.50	10.009	0.16	0.0799
0.40	10.068	0.219	0.0884
0.34	10.125	0.276	0.0941
0.30	10.172	0.323	0.0958
0.25	10.222	0.373	0.0932
0.20	10.296	0.447	0.0898
0.15	10.4	0.551	0.0803
0.11	10.482	0.633	0.0716
0.09	10.542	0.693	0.0643
0.08	10.585	0.736	0.0577
0.06	10.646	0.797	0.0479
0.05	10.664	0.815	0.0396

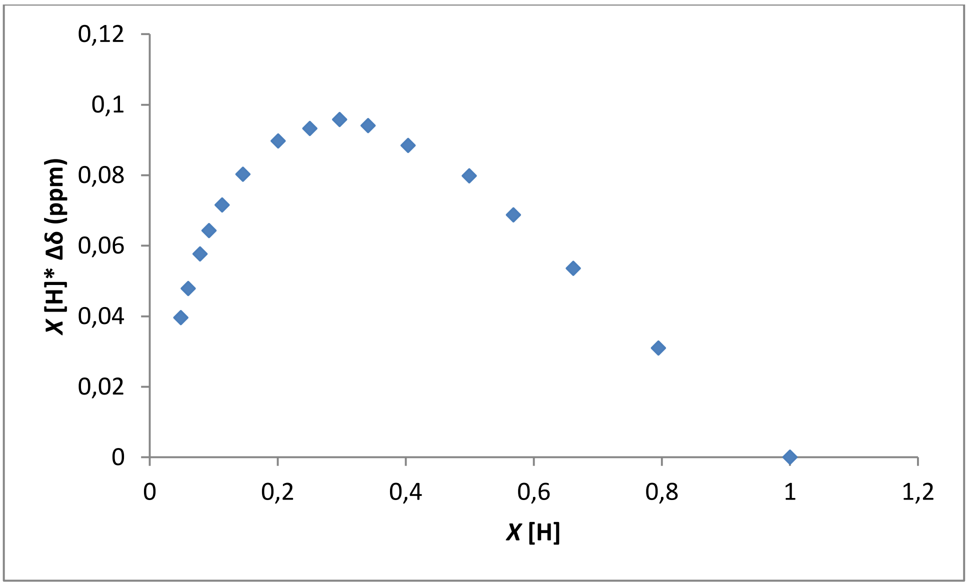

Figure S67. Job plot in DMSO-d_6 for the interaction of H_4-**2**$(BF_4)_4$ and ibuprofen$^-$ ($[H]=2\times10^{-3}$ M).

c. H_4-**2**$(BF_4)_4$ with NBu_4NO_3

Table S18. Data values of the Job plot of H_4-**2**$(BF_4)_4$ with NO_3^-

X[H]	δ (ppm)	Δδ (ppm)	X[H]*Δδ (ppm)
1.00	9.848	0	0
0.79	9.849	0.001	0.0008
0.66	9.851	0.003	0.002
0.56	9.852	0.004	0.0023
0.49	9.853	0.005	0.0025
0.40	9.854	0.006	0.0024
0.33	9.856	0.008	0.0027
0.25	9.86	0.012	0.003
0.20	9.862	0.014	0.0028
0.14	9.867	0.019	0.0027
0.09	9.875	0.027	0.0025
0.06	9.879	0.031	0.0018
0.05	9.881	0.033	0.0016

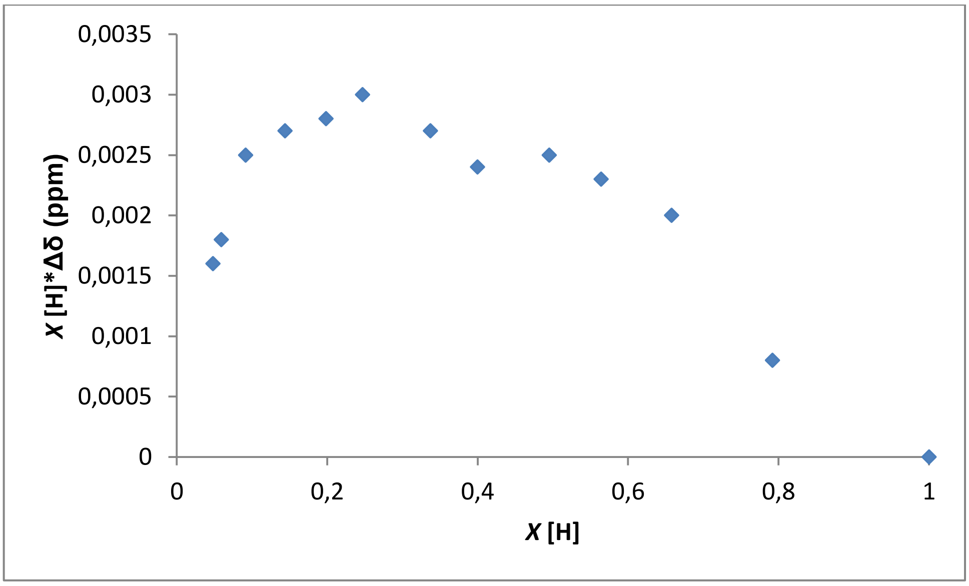

Figure S68. Job plot in DMSO-d_6 for the interaction of H_4-**2**$(BF_4)_4$ and NO_3^- ([H]=2×10^{-3}M).

d. H_4-**2**(BF_4)$_4$ with NBu_4Cl

Table S19. Data values of the Job plot of H_4-**2**(BF_4)$_4$ with Cl^-.

X[H]	δ (ppm)	Δδ (ppm)	X[H]*Δδ (ppm)
1.00	9.847	0.000	0.0000
0.79	9.863	0.016	0.0127
0.66	9.879	0.032	0.0210
0.56	9.892	0.045	0.0253
0.49	9.903	0.056	0.0276
0.40	9.928	0.081	0.0323
0.34	9.947	0.100	0.0336
0.29	9.966	0.119	0.0347
0.25	9.988	0.141	0.0346
0.20	10.017	0.170	0.0335
0.14	10.063	0.216	0.0308
0.11	10.101	0.254	0.0281
0.09	10.130	0.283	0.0257
0.08	10.155	0.308	0.0236
0.06	10.180	0.333	0.0196
0.05	10.191	0.344	0.0164

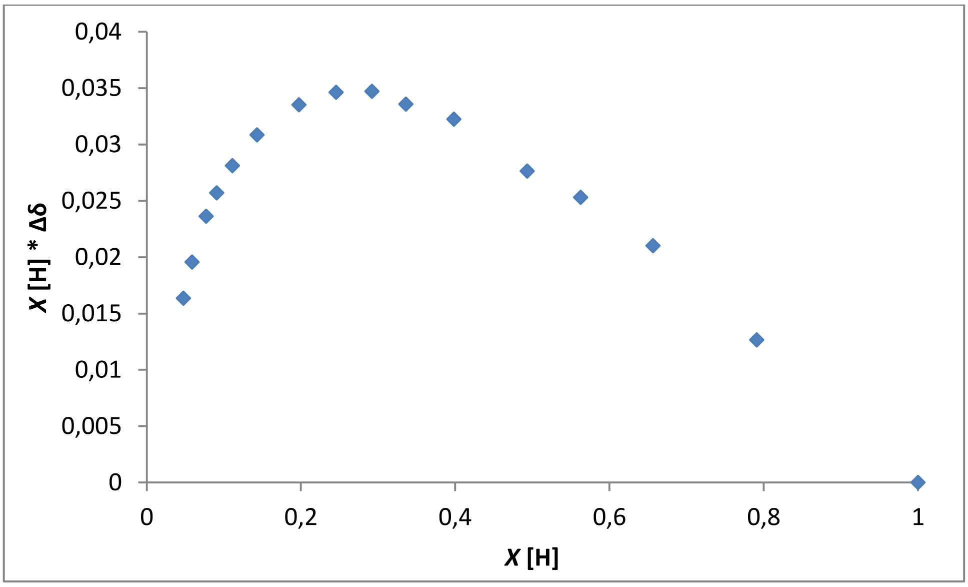

Figure S69. Job plot in DMSO-d_6 for the interaction of H_4-**2**(BF_4)$_4$ and Cl^- ([H]=2×10^{-3}M).

e. H_4-**2**$(BF_4)_4$ with NBu_4benzoate

Table S20. Data values of the Job plot of H_4-**2**$(BF_4)_4$ with benzoate

X[H]	δ (ppm)	Δδ (ppm)	X[H]*Δδ (ppm)
1.00	9.848	0.000	0.0000
0.79	9.880	0.032	0.0253
0.66	9.912	0.064	0.0420
0.56	9.944	0.096	0.0540
0.49	9.971	0.123	0.0607
0.40	10.019	0.171	0.0681
0.34	10.059	0.211	0.0709
0.29	10.092	0.244	0.0713
0.25	10.132	0.284	0.0698
0.20	10.188	0.340	0.0671
0.14	10.242	0.394	0.0563
0.11	10.298	0.450	0.0499
0.09	10.346	0.498	0.0453
0.08	10.389	0.541	0.0416
0.06	10.432	0.584	0.0344
0.05	10.460	0.612	0.0291

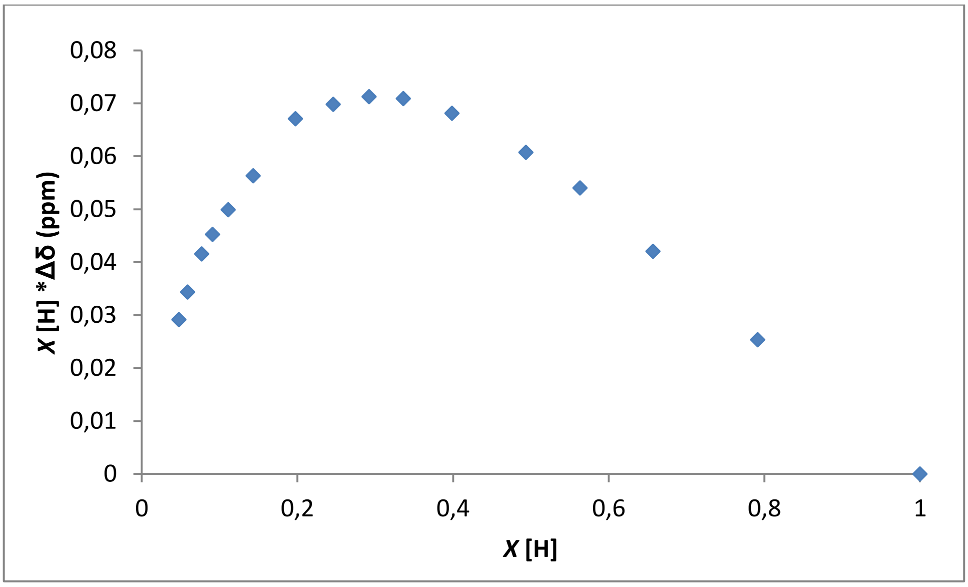

Figure S70. Job plot in DMSO-d_6 for the interaction of H_4-**2**$(BF_4)_4$ and benzoate ([H]=2×10^{-3}M).

f. H_4-2$(BF_4)_4$ with NBu_4Br

Table S21. Data values of the Job plot of H_4-**2**$(BF_4)_4$ with Br^-

X[H]	δ (ppm)	Δδ (ppm)	X[H]*Δδ (ppm)
1.00	9.846	0.000	0.0000
0.79	9.852	0.006	0.0048
0.66	9.857	0.011	0.0072
0.56	9.863	0.017	0.0096
0.50	9.867	0.021	0.0104
0.40	9.875	0.029	0.0116
0.34	9.882	0.036	0.0122
0.29	9.889	0.043	0.0126
0.25	9.897	0.051	0.0126
0.20	9.907	0.061	0.0121
0.14	9.927	0.081	0.0116
0.11	9.945	0.099	0.0110
0.09	9.957	0.111	0.0102
0.08	9.967	0.121	0.0094
0.06	9.979	0.133	0.0079
0.05	9.985	0.139	0.0067

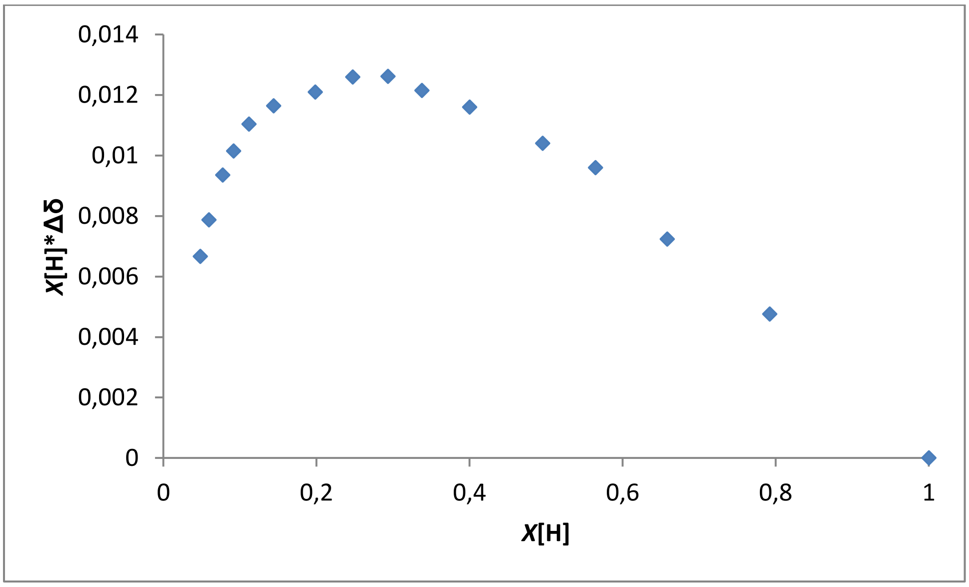

Figure S71. Job plot in DMSO-d_6 for the interaction of H_4-**2**$(BF_4)_4$ and Br^- ([H]=2×10^{-3}M).

g. H_8-**5**$(BF_4)_8$ with NBu_4ATP

Table S22. Data values of the Job plot of H_8-**5**$(BF_4)_8$ with ATP^-

X[H]	δ (ppm)	Δδ (ppm)	X[H]*Δδ (ppm)
1.00	9.714	0.000	0.0000
0.79	9.715	0.001	0.0008
0.65	9.721	0.007	0.0046
0.56	9.735	0.021	0.0118
0.49	9.747	0.033	0.0162
0.40	9.786	0.072	0.0286
0.33	9.821	0.107	0.0358
0.29	9.848	0.134	0.0389
0.24	9.882	0.168	0.0410
0.20	9.922	0.208	0.0408
0.14	9.965	0.251	0.0356
0.11	9.979	0.265	0.0292
0.09	9.991	0.277	0.0250
0.08	9.989	0.275	0.0210
0.06	9.997	0.283	0.0165
0.05	9.995	0.281	0.0133

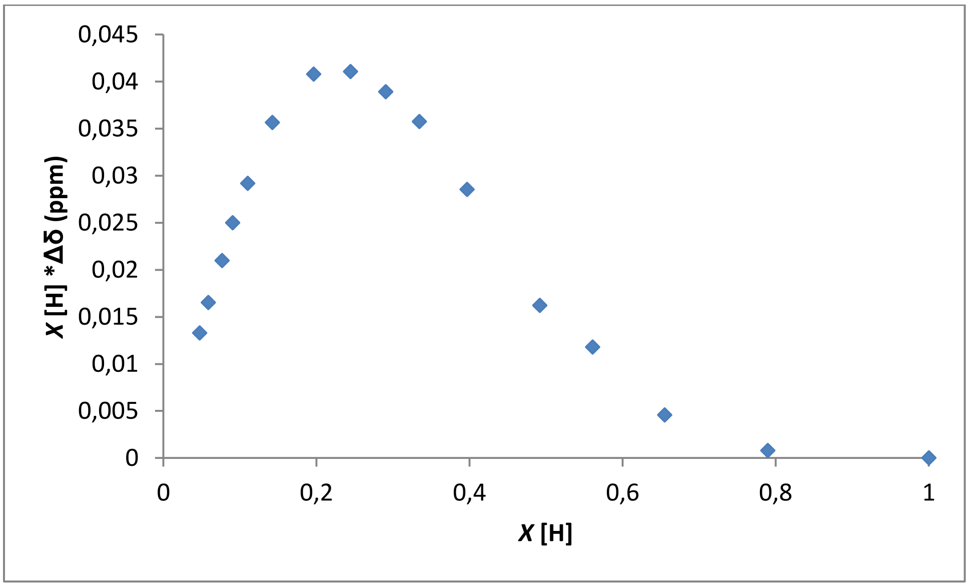

Figure S72. Job plot in DMSO-d_6 for the interaction of H_8-**5**$(BF_4)_8$ and ATP^- ([H]=5×10^{-4}M).

h. H_8-**5**$(BF_4)_8$ with NBu_4ibuprofen

Table S23. Data values of the Job-Plot of **5**$(BF_4)_8$ with ibuprofen)$^-$

X[H]	δ (ppm)	Δδ (ppm)	X[H]*Δδ (ppm)
1.00	9.708	0.000	0.0000
0.79	9.709	0.001	0.0008
0.66	9.722	0.014	0.0092
0.56	9.748	0.040	0.0225
0.49	9.778	0.070	0.0344
0.40	9.850	0.142	0.0564
0.33	9.916	0.208	0.0696
0.29	9.993	0.285	0.0829
0.24	10.084	0.376	0.0920
0.20	10.208	0.500	0.0982
0.14	10.382	0.674	0.0958
0.11	10.496	0.788	0.0869
0.09	10.568	0.860	0.0777
0.08	10.617	0.909	0.0694
0.06	10.671	0.963	0.0563
0.05	10.686	0.978	0.0463

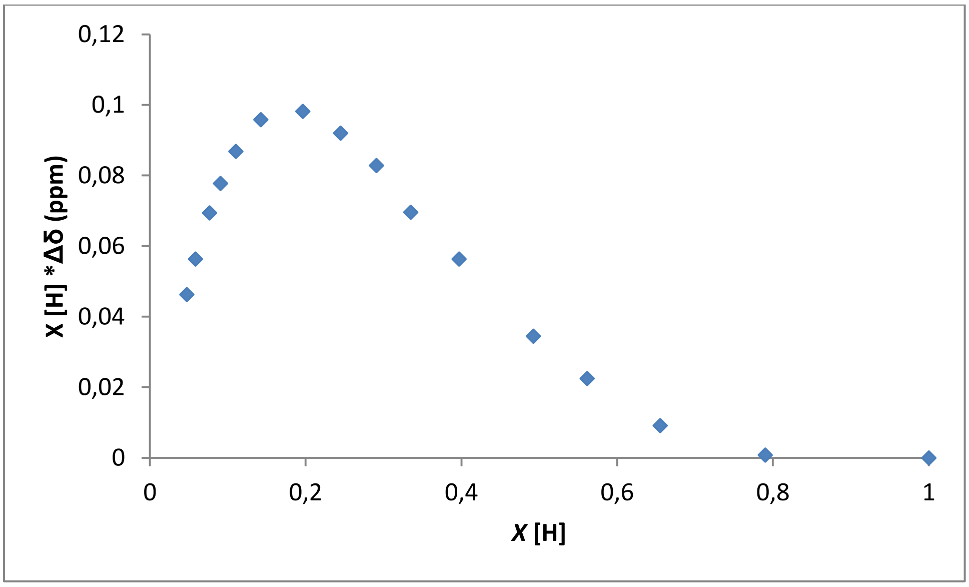

Figure S73. Job plot in DMSO-d_6 for the interaction of H_8-**5**$(BF_4)_8$ and ibuprofen$^-$ ([H]=2×10^{-3}M).

i. H_8-**5**$(BF_4)_8$ with NBu_4benzenesulfonate

Table S24. Data values of the Job plot of H8-**5**$(BF_4)_8$ with benzenesulfonate

X[H]	δ (ppm)	Δδ (ppm)	X[H]*Δδ (ppm)
1.00	9.706	0.000	0.0000
0.79	9.710	0.004	0.0032
0.66	9.713	0.007	0.0046
0.56	9.715	0.009	0.0051
0.49	9.718	0.012	0.0059
0.40	9.723	0.017	0.0068
0.34	9.728	0.022	0.0074
0.29	9.732	0.026	0.0076
0.25	9.738	0.032	0.0079
0.20	9.748	0.042	0.0083
0.14	9.763	0.057	0.0081
0.11	9.777	0.071	0.0079
0.09	9.787	0.081	0.0074
0.08	9.795	0.089	0.0068
0.06	9.805	0.099	0.0058
0.05	9.807	0.101	0.0048

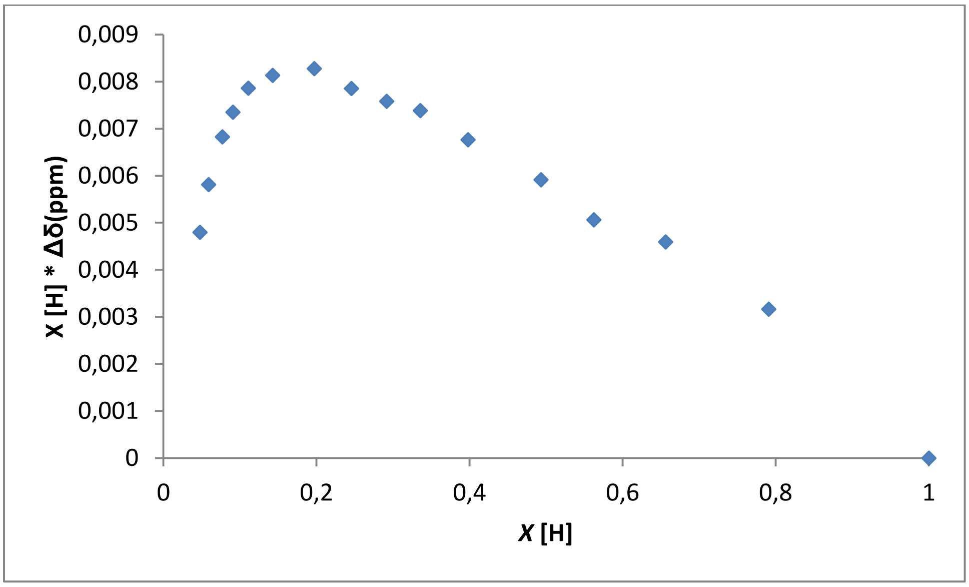

Figure S74. Job plot in DMSO-d_6 for the interaction of H_8-**5**$(BF_4)_8$ and benzenesulfonate ($[H]=2\times10^{-3}$M).

j. H_8-**5**$(BF_4)_8$ with NBu_4Cl

Table S25. Data values of the Job plot of H_8-**5**$(BF_4)_8$ with Cl^-

X[H]	δ (ppm)	Δδ (ppm)	X[H]*Δδ (ppm)
1.00	9.705	0.000	0.0000
0.79	9.708	0.003	0.0024
0.66	9.717	0.012	0.0079
0.56	9.730	0.025	0.0141
0.49	9.745	0.040	0.0197
0.40	9.777	0.072	0.0287
0.34	9.810	0.105	0.0353
0.29	9.843	0.138	0.0403
0.25	9.887	0.182	0.0447
0.20	9.947	0.242	0.0477
0.17	9.996	0.291	0.0486
0.14	10.038	0.333	0.0475
0.12	10.081	0.376	0.0466
0.11	10.110	0.405	0.0449
0.10	10.135	0.430	0.0429
0.09	10.165	0.460	0.0410
0.08	10.180	0.475	0.0393
0.08	10.197	0.492	0.0375
0.07	10.211	0.506	0.0361
0.07	10.223	0.518	0.0344
0.06	10.236	0.531	0.0329
0.06	10.244	0.539	0.0316
0.05	10.256	0.551	0.0290
0.05	10.267	0.562	0.0267

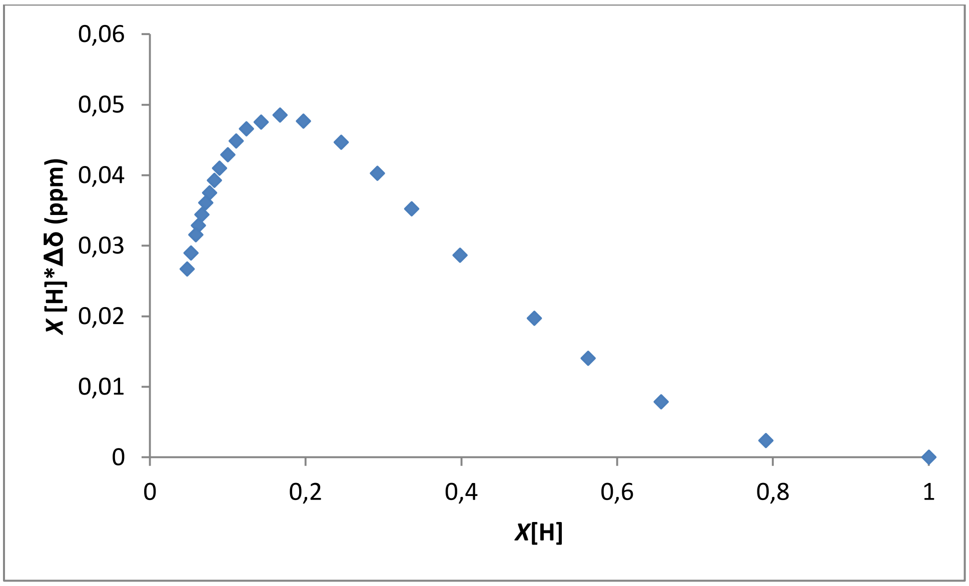

Figure S75. Job plot in DMSO-d_6 for the interaction of H_8-**5**$(BF_4)_8$ and Cl^- ([H]=2×10^{-3}M).

k. H_8-**5**$(BF_4)_8$ with NBu_4benzoate

Table S26. Data values of the Job plot of H_8-**5**$(BF_4)_8$ with benzoate

X[H]	δ (ppm)	Δδ (ppm)	X[H]*Δδ (ppm)
1.00	9.706	0.000	0.0000
0.79	9.709	0.003	0.0024
0.66	9.724	0.018	0.0118
0.56	9.751	0.045	0.0253
0.49	9.782	0.076	0.0375
0.40	9.847	0.141	0.0561
0.34	9.914	0.208	0.0698
0.29	9.969	0.263	0.0767
0.25	10.050	0.344	0.0845
0.20	10.157	0.451	0.0889
0.14	10.299	0.593	0.0847
0.11	10.399	0.693	0.0767
0.09	10.464	0.758	0.0688
0.08	10.512	0.806	0.0618
0.06	10.550	0.844	0.0496
0.05	10.568	0.862	0.0410

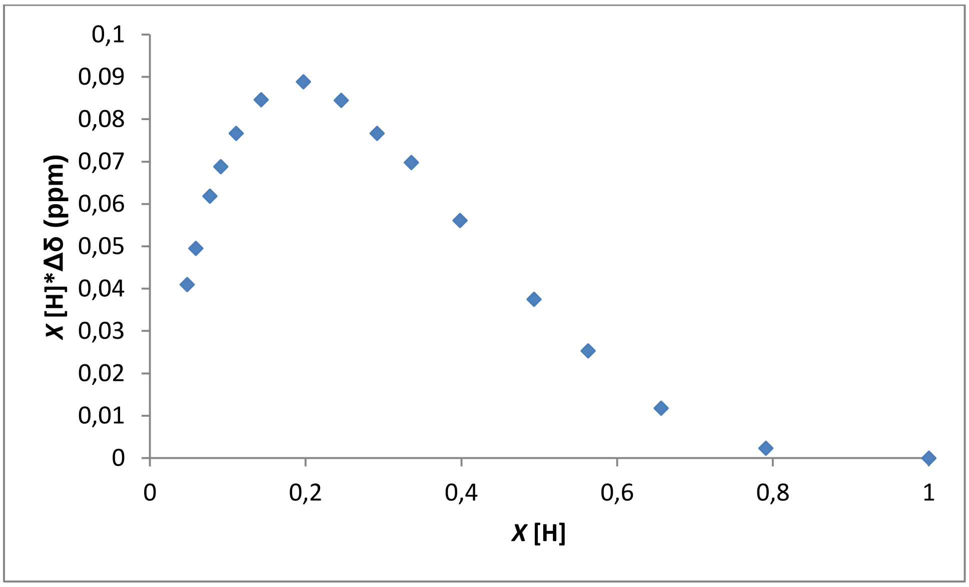

Figure S76. Job plot in DMSO-d_6 for the interaction of H_8-**5**$(BF_4)_8$ and benzoate ($[H]=2\times10^{-3}$M).

l. H_8-**5**$(BF_4)_8$ with NBu_4Br

Table S27. Data values of the Job plot of H_8-**5**$(BF_4)_8$ with Br^-

X[H]	δ (ppm)	Δδ (ppm)	X[H]*Δδ (ppm)
1.00	9.707	0.000	0.0000
0.79	9.720	0.013	0.0103
0.66	9.739	0.032	0.0210
0.56	9.753	0.046	0.0259
0.49	9.767	0.060	0.0296
0.40	9.790	0.083	0.0330
0.34	9.811	0.104	0.0349
0.29	9.828	0.121	0.0353
0.25	9.851	0.144	0.0353
0.20	9.880	0.173	0.0341
0.15	9.915	0.208	0.0311
0.11	9.953	0.246	0.0272
0.09	9.975	0.268	0.0243
0.08	9.992	0.285	0.0219
0.06	10.011	0.304	0.0178
0.05	10.017	0.310	0.0147

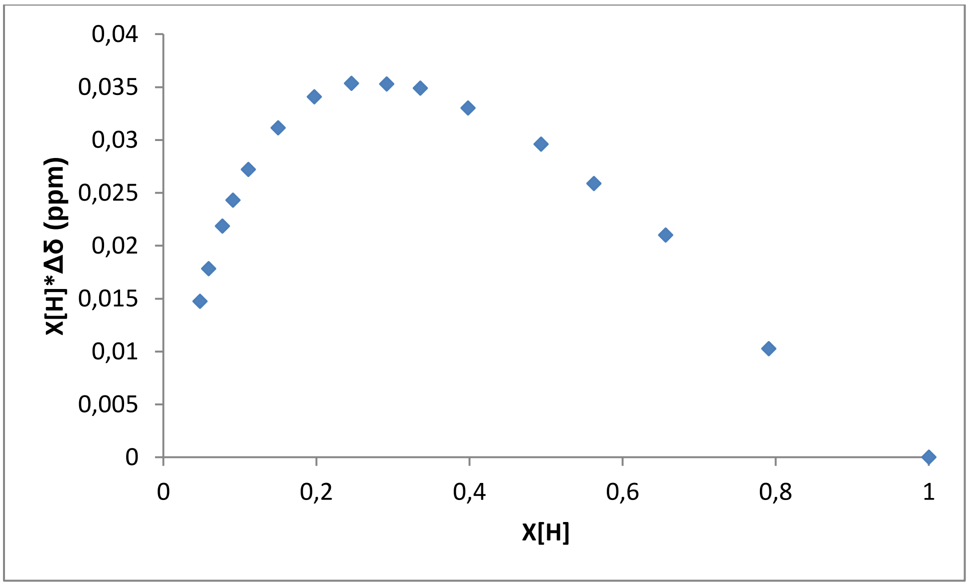

Figure S77. Job plot in DMSO-d_6 for the interaction of H_8-**5**$(BF_4)_8$ and Br^- ([H]=2×10^{-3}M).

6. DOSY experiments

The experiments were carried out in DMSO-d_6, at constant concentrations of 5 mM.

Table S28. Diffusion constants of the salts and silver complexes

Compound	G (m^2/s)
H_4-**2**$(Br)_4$	$4.94 \cdot 10^{-10}$
complex [**3**]$(BF_4)_4$	$5.36 \cdot 10^{-10}$
complex [**4**]$(BF_4)_4$	$7.17 \cdot 10^{-10}$
H_8-**5**$(BF_4)_8$	$2.71 \cdot 10^{-10}$

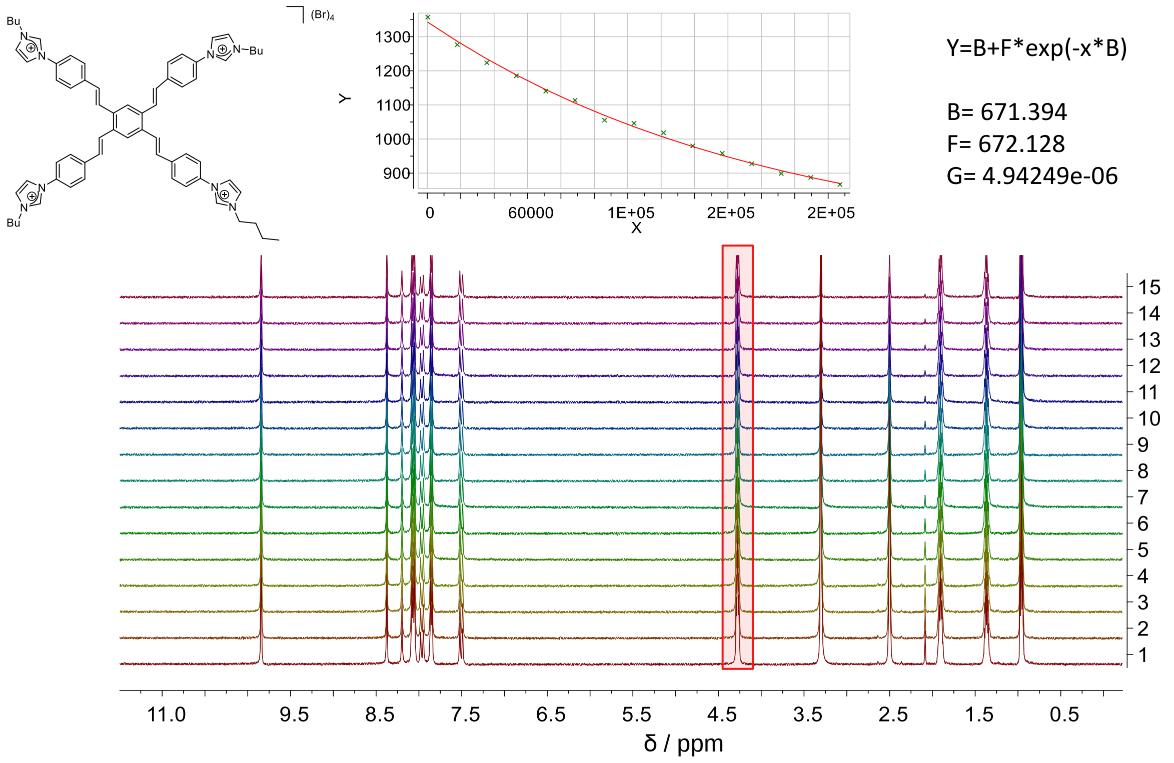

Figure S78. DOSY spectra of H_4-**2**$(Br)_4$ in DMSO-d_6 (c = 5×10^{-3}M).

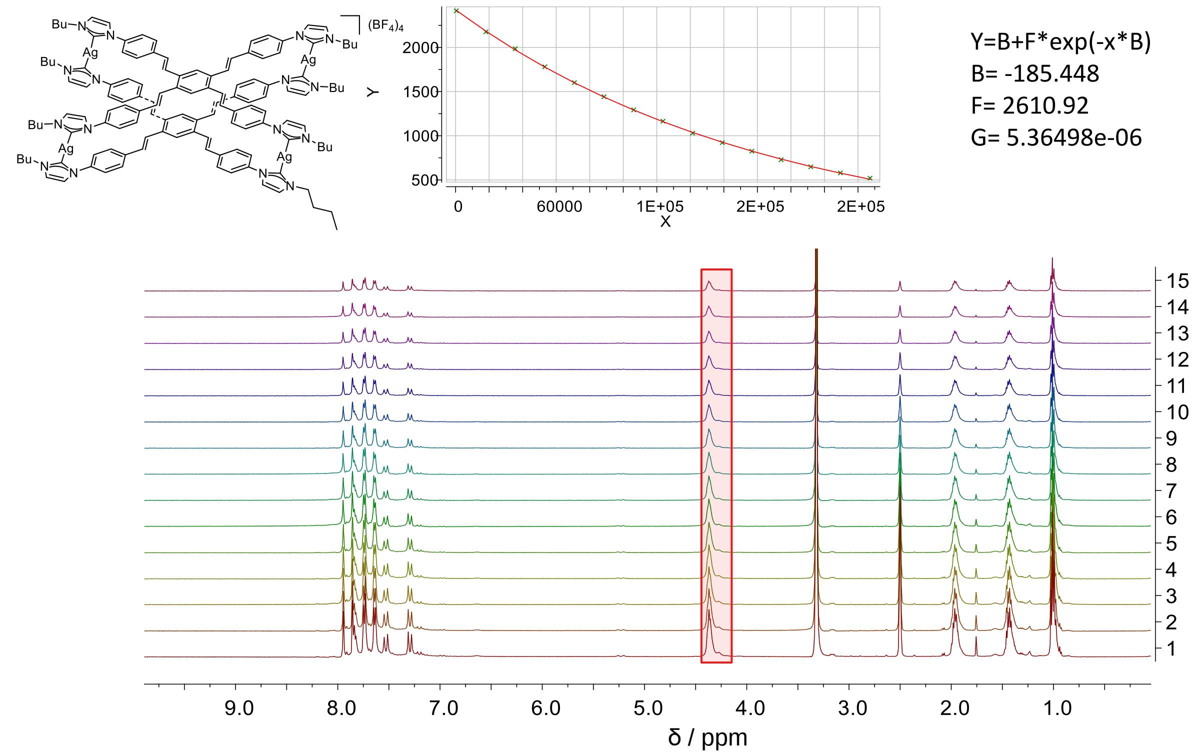

Figure S79. DOSY spectra of [**3**]$(BF_4)_4$ in DMSO-d_6 (c = 5×10^{-3}M).

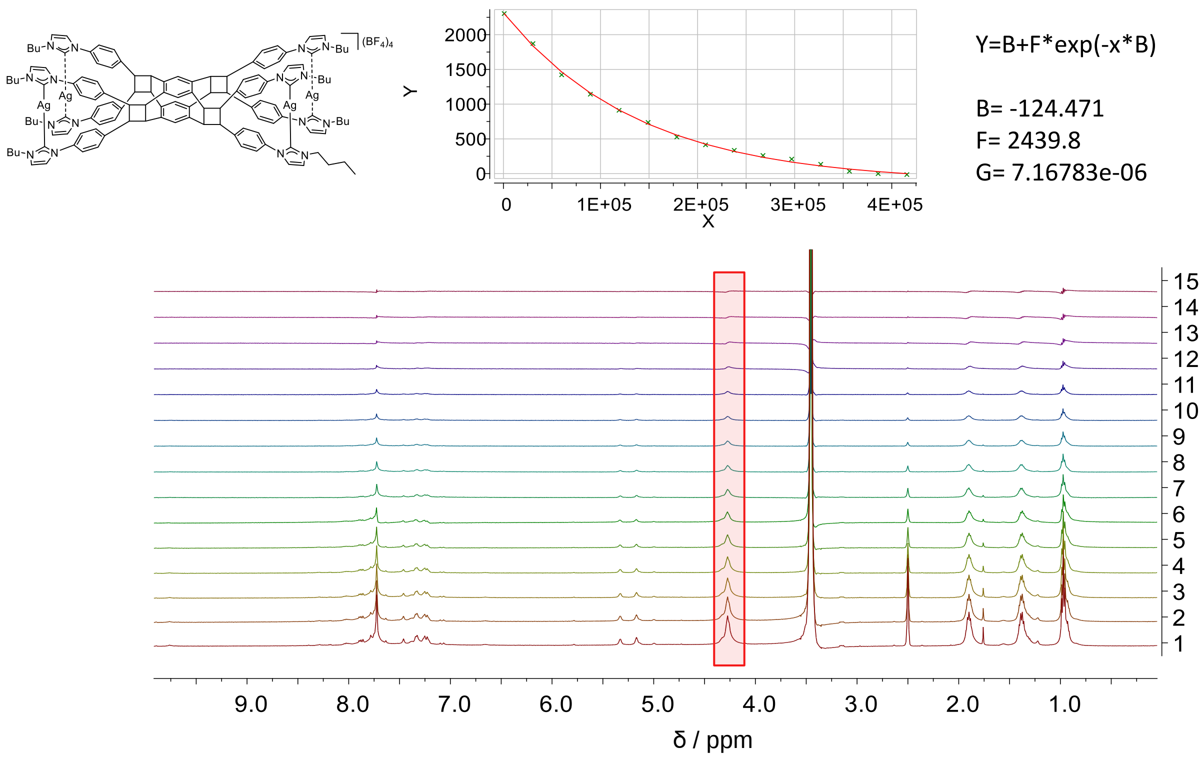

Figure S80. DOSY spectra of [**4**]$(BF_4)_4$ in DMSO-d_6 (c = 5×10^{-3}M).

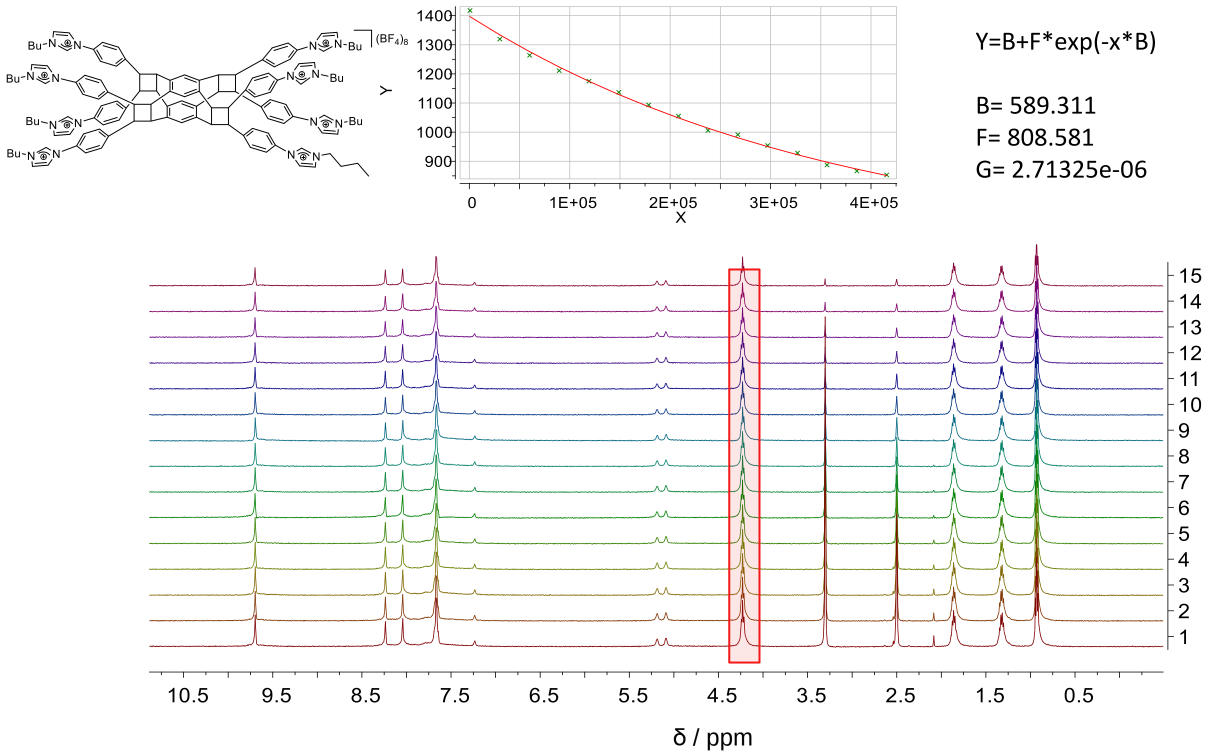

Figure S81. DOSY spectra of H_8-**5**$(BF_4)_8$ in DMSO-d_6 (c = 5×10^{-3}M).

7. References

[1] N. E. Wong, P. Ramaswamy, A. S. Lee, B. S. Gelfand, K. J. Bladek, J. M. Taylor, D. M. Spasyuk, G. K. H. Shimizu, *J. Am. Chem. Soc.* **2017**, *139*, 14676–4683.

[2] Z. Zheng, Z. Yu, M. Yang, F. Jin, Q. Zhang, H. Zhou, J. Wu, Y. Tian, *J. Org. Chem.* **2013**, *78*, 3222–3234.

[3] O. V. Dolomanov, L. J. Bourhis, R. J. Gildea, J. A. K. Howard, H. Puschmann, *OLEX2, J. Appl. Crystallogr.* **2009**, *42*, 339–341.

[4] G. M. Sheldrick, SHELXT – Integrated space-group and crystal-structure determination. *Acta Crystallogr., Sect. A: Found. Crystallogr.* **2015**, *71*, 3–8.

[5] G. M. Sheldrick, Crystal structure refinement with SHELXL. *Acta Crystallogr., Sect. C: Struct. Chem.* **2015**, *71*, 3–8.

[6] a) A. J. Lowe, F. M. Pfeffer, P. Thordarson, *Supramol. Chem*. **2012**, *24*, 585–594; b) P. Thordarson, *Chem. Soc. Rev.* **2011**, *40*, 1305–1323.

[7] L. K. S. von Krbek, C. A. Schalley, P. Thordarson, *Chem. Soc. Rev.* **2017**, *46*, 2622–2637.
